# Poisoning cases in the German crime series *Tatort* (crime scene) from 1974 to 2022

**DOI:** 10.1007/s00210-022-02281-9

**Published:** 2022-08-16

**Authors:** Rachel Ellerbeck, Roland Seifert

**Affiliations:** Institute of Pharmacology, Hannover Medical School, Carl-Neuberg-Str. 1, 30625 Hanover, Germany

**Keywords:** *Tatort* (crime scene), Poisoning, Drugs, Fiction–reality comparison, Public awareness

## Abstract

Poisoning occurs frequently in TV crime series but, to the best of our knowledge, has not yet been analyzed scientifically. This study examines the plausibility of poisoning cases in Germany’s most popular crime series, *Tatort* (crime scene), from 1974 to 2022. In the TV series, the increasing rate of poisoning in Germany as well as the increasing variety of substances leading to poisoning over the years are depicted. Largely in line with reality, similar substance categories and routes of administration are presented. However, poisoning outcomes in *Tatort* differ from reality: over 50% of the victims die in *Tatort*, whereas in reality, more than 80% survive. In > 95% of the episodes, the mechanism of action of a poison is not explained, omitting an important opportunity for raising public awareness. The TV series also deviates from reality in terms of the etiology of poisonings: External poison delivery is largely overrepresented, while the high rate of accidental poisonings in real life is underrepresented. Almost no accidental poisonings occur in *Tatort*, although this is the most frequent type of poisoning in real life. In *Tatort*, men are overrepresented as offenders and victims of poisoning compared to reality. Thus, the crime series does not convey the message that anyone can be a potential victim of poisoning and that particularly vulnerable groups need proper education and the best possible protection. This paper discusses the conflict between detailed, plausible episodes with cases of poisoning and the potential for imitation that they may cause.

**Supplementary Information:**

The online version contains supplementary material available at 10.1007/s00210-022-02281-9.

## Introduction

The crime series *Tatort* is Germany’s most popular crime series, with an average of 10 million linear viewers. It is a joint production by the German broadcaster ARD, the Austrian TV station, and Swiss Radio and Television. The first episode was broadcasted on West-German television back in 1970. Thus, with > 1.200 episodes broadcasted, the series is the longest-running German crime series. Through marketing in 50 countries, the series also gained international significance (https://de.wikipedia.org/w/index.php?title=Tatort_(television series)&oldid = 221,349,217, accessed 21 March 2022).

Although poisoning is frequently depicted in TV crime series, this topic, to the best of our knowledge, has not yet been investigated scientifically. This motivated us to investigate *Tatort* regarding cases of poisoning. We intended to uncover errors in the presentation of poisonings and to compare these data with reality. With poisoning figures increasing (Giftinformationszentrum (GIZ) Mainz, Germany 2022), the need for public awareness about potential hazards and prevention of poisoning is becoming greater. Since this affects all population groups, an easily accessible educational medium is needed. As such, a television series with a wide reach and a high number of viewers is a good tool to raise public awareness.

## Materials and methods

### Analysis of the individual *Tatort* episodes

**Fig. 1:**
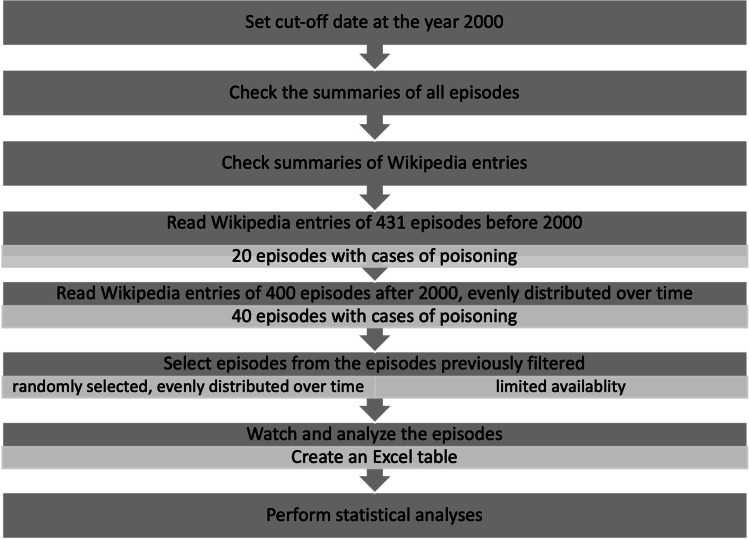
Statistical procedure for analyzing individual *Tatort* episodes, shown in a flowchart

Figure 1 shows a flowchart of episode selection and analysis. A cutoff was set in the year 2000. All episodes broadcast before 2000 are considered “old,” and all episodes broadcast after 2000 are considered “new.” Due to the particularly limited availability of old episodes, the cutoff is not set in the middle between 1974 and 2022 but shifted somewhat further up.

To identify *Tatort* episodes with poisonings, it was first necessary to obtain summaries of the individual episodes. The Wikipedia entries for each episode (https://de.wikipedia.org/w/index.php?title=Wikipedia&oldid=221288867, last accessed on 25 March 2022) were analyzed. All the summaries of the 431 old episodes were read, and the episodes with poisonings were filtered. Of the 758 new episodes, 400 summaries were read. The content notes of 400 new episodes were read since about the same number of content notes should be analyzed as the old episodes, but at the same time, it was expected that more poisoning cases would be found among the new episodes. Since there was already enough material among the 400 summaries, it was not necessary to read more episode summaries. Episodes were selected randomly but evenly distributed between 2000 and 2022. Twenty old and 40 new episodes with poisoning scenes were filtered and subsequently re-selected at random. Bias was avoided due to the double randomized selection.

The selected episodes were then viewed multiple times and at different speeds, and their plausibility and comprehensiveness were rated. For this purpose, a comprehensive table (Suppl. Table 1) with a rating system for plausibility and comprehensiveness (school grading system: 1 best result; 6 worst results) was created. After rating, all episodes with P: 1–3 (plausibility) and D: 1–3 (detailedness) were filtered out as the best episodes, and those with P: 4–6 and D: 4–6 as the worst episodes. A total of 45 episodes were analyzed (13 old episodes before 2000 and 32 new episodes after 2000).

### Statistical analyses of all episodes

With the help of the Suppl. Table 1, further analyses were carried out. First, all substances involved in poisonings were assigned to 13 substance categories. A comparison of the substance categories in old and new episodes was made. Table 1 was created to list substances involved in poisoning for each episode, their mechanisms of action, and their symptoms. In addition, the table shows whether symptoms, effects, and substance explanations were mentioned in the episodes.

**Table 1:** Analysis of active ingredients, mechanism of action, poisoning outcome, and mention in the crime scene

Episode	Active ingredient/active ingredient group	Mechanism of action	References	Poisoning outcome	Information provided
1187	**Frog’s toxin:** alkaloid toxins (1); main alkaloid = pumiliotoxin (PTX) (1)	**Pumiliotoxin:** affects calcium channels (1), increases sodium influx in cerebral synaptoneurosomes (2); cardiotonic, myotonic (2)	**(1)** Correa et al. (2021); **(2)** Gusovsky et al. (1988); **(3)** Loose (2022)	Survival	Mechanism of action: no; toxin name: no; symptoms: yes
1186	**Scopolamine:** natural alkaloid of solanaceous plants (1)	**Scopolamine:** nonselective muscarinic antagonism (2); central sedative, antiemetic, and amnestic effects (2)	**(1)** Aktories et al. (2013a, p. 132); **(2)** Renner et al. (2005); **(3)** Meletzky (2022)	Survival	Mechanism of action: no; symptoms: yes
1174	**NanoBots:** production of artificial proteins, nanorobots under development (1)	No mechanism because fictitious	**(1)** Lehmann (2021); **(2)** Marka (2021)	Death	Fictitious effect: yes; symptoms: yes
1114	**Phorbol:** from spurge family (Euphorbiaceae) (1)	**Phorbol:** skin irritation (local), toxic, and cocarcinogenic (1); fatal necrotizing/hemorrhagic gastroenteritis, renal injury (1)	**(1)** Aktories et al. (2013h, p. 1080); **(2)** Henning (2019)	Death	Mechanism of action: no; symptoms: yes
1107	**Potassium cyanide (KCN):** cyanide (2); **cannabis:** active ingredient tetrahydrocannabinol (1); **ecstasy: 3,4-methylenedioxy-methamphetamine (MDMA):** abuse popular in the 1980s (3)	**KCN:** complex formation—> blocking of cytochrome oxidases (2); interruption of the respiratory chain (2); **cannabis:** analgesic, muscle relaxant, antiemetic (1); **MDMA:** inhibition of dopamine and norepinephrine reuptake (4)	**(1)** Hardman et al. (2001d, p. 637); **(2)** Hardman et el. (2001e, p. 1893); **(3)** Hardman et al. (2001d, p. 6390; **(4)** Dekant and Vamvakas (2010a, pp. 236ff); **(5)** Chahoud (2019)	**KCN:** death; **cannabis:** mentioned only in passing; **MDMA:** mentioned only in passing	Mechanism of action of all three substances: no; symptoms: yes
1096	**Fentanyl patches:** Increasing problem: dependence (1); **Cannabis:** active ingredient tetrahydrocannabinol (2)	**Fentanyl patch:** full MOR agonist (1); inhibition of excitatory neurotransmitter release (1); **cannabis:** cannabinoid receptors: analgesic, muscle relaxant, antiemetic (2)	**(1)** Seifert (2019e, f, pp. 121, 129); **(2)** Hardman et al. (2001d, p. 637); **(3)** Kleinert (2019)	**Fentanyl patch:** death; **cannabis:** mentioned only in passing	Mechanism of action of both substances: no; symptoms: no
1085	**Dibenzodioxin:** most potent representative: 2,3,7,8-tetrachlorodibenzodioxin (TCDD) (1); **Yukon powder:** fictitious	**Dibenzodioxin:** tumor-promoting toxic effect through Ah-receptor: regulation protein expression for carcinogenic hydrocarbons (1); **Yukon powder:** no mechanism because fictitious	**(1)** Aktories et al. (2013d, g, pp. 1005f, 1055f); **(2)** Marka (2019)	**Dibenzo-dioxin:** abortion, maternal survival; **Yukon powder:** death	Mechanism of action: no; symptoms: yes
1058	**Vacor:** rodenticide (1); causes insulin-dependent diabetes mellitus (1)	**Vacor:** suppression of insulin release independent of cAMP and C-kinase (2)	**(1)** Esposti et al. (1996); **(2)** Taniguchi et al. (1989); **(3)** Dag (2018)	Death	Mechanism of action: no; Symptoms: yes
1051	**Cholinesterase inhibitors:** acetylcholinesterase inhibitors (2); **glutamate antagonists:** NMDAR antagonist (2); **antidepressants:** treatment of depression (2); **citalopram:** SSRI (2); **antipsychotics:** drugs to “eliminate or attenuate psychopathological syndromes and mental illness” (1)	**Cholinesterase inhibitors:** acetylcholine’s residence time is extended (2); **glutamate antagonists:** decreased glutamate sensitivity (2); **antidepressants:** normalize the neurotransmitter deficit (2); **citalopram:** block serotonin reuptake (2)	(**1**) Aktories et al. (2013b, p. 294); (**2**) Seifert (2019b, l, m, pp. 78, 332, 358); (**3**) Koch (2018)	Survival	Mechanism of action: no; symptoms: yes
1046	**Rabies virus:** family: Rhabdoviridae, genus: Lyssavirus (1); **turpentine:** fluid derived from pine and larch resin (3)	**Rabies virus:** local replication (2); migration to CNS (2); replication (2); **turpentine:** contact dermatitis—> irritant effect (4); i.v.: bloody sputum, pneumonia (5); overdose: death (5)	**(1)** Series editing “RKI-Ratgeber” (2020); **(2)** Brunker and Mollentze (2018); **(3)** Bayerische Landesanstalt (2012); **(4)** Booken et al. (2006); **(5)** DIE WELT (2012); **(6)** Zahavi (2018)	**Rabies virus:** death; **turpentine:** death	Mechanism of action: no; symptoms: yes
1038	Substance with no name	No mechanism because no substance is specified	No literature because no substance is specified; **(1)** Baxmeyer (2017)	Death	Mechanism of action: no; symptoms: no
1037	**Benzene:** solvent (4); **toluene:** solvent (4); **radioactive metals:** wastes from industry, diagnostics, therapeutics (1); **mercury:** metallic and organic (1); **cyanobacteria/blue-green algae:** in fresh and brackish water (2); endo-, cyto-, neuro-, and hepatotoxins (2)	**Benzene:** damage to the hematopoietic system (4); carcinogenic (4); **toluene:** “CNS depressant” (4); **radioactive metals:** radiation emission, chemical toxicity (1); **mercury:** reaction with free SH groups of proteins (1); **cyanobacteria:** mechanism still unclear (3)	**(1)** Aktories et al. (2013e, f, pp. 1023f, 1029); **(2)** Thebault et al. (1995); **(3)** van Riel et al. (2007); **(4)** Hardman et al. (2001e, p. 1893); **(5)** Bernardi (2017)	Survival	Mechanism of action: yes; symptoms: yes
1018	**Carbon monoxide (CO):** gas (1); source: combustion processes (transport, industry, engines) (1)	**CO:** complex formation with hemoglobin (1); blocks oxygen binding site (1)	**(1)** Hardman et al. (2001e, pp. 1880ff); **(2)** Imboden (2017)	Death	Mechanism of action: no; symptoms: no
1012	**Poppy seeds (*Papaver somniferum*):** seeds of the opium poppy (3); few cases of poppy seed allergy known (3)	**Poppy seeds:** type 1 and type 3 immunological reactions and non-immunological reactions (1); mast cell activation and histamine release (2)	**(1)** Thiel (1991); **(2)** Seifert (2019a, p. 52); **(3)** Senti et al. (2000); **(4)** Ranisch (2017)	Death	Mechanism of action: no; symptoms: yes
1010	**Ricin:** from *Ricinus communis* (Euphorbiaceae) (1); toxin = lectin (1); in seeds (1); acid-stable (1)	**Ricin:** blockade of protein biosynthesis (1); necroses of the gastrointestinal tract, liver, kidney, and spleen (1)	**(1)** Aktories et al. (2013h, pp. 1075f); **(2)** Marka (2017)	Survival	Mechanism of action: no; symptoms: yes
1009	**Ecstasy: 3,4- methylenedioxy-methamphetamine (MDMA):** abuse popular in the 1980s (1); **benzodiazepines:** frequent clinical application (2); drug group (2); **barbiturates:** sedatives, hypnotics, and injection narcotics (2); **analgesics:** drugs for the treatment of pain (2); opioid analgesics and non-opioid analgesics (2)	**MDMA:** inhibition of dopamine and norepinephrine reuptake (1); **benzodiazepines:** enhancement of GABAergic inhibition (2); anxiolytic, sedative-hypnotic, muscle relaxant, and antiepileptic (2); **barbiturates:** enhanced inhibition of excitatory neurotransmission, enhancement of GABAergic inhibitory transmission (2); **analgesics:** agonism and/or antagonism at pain receptors (2)	**(1)** Dekant and Vamvakas (2010a, pp. 236ff); **(2)** Seifert (2019d, j, k, pp. 118ff, 310ff, 328); **(3)** Spirandelli (2017)	Death	Mechanism of action: no; symptoms: yes
995	**Knockout drops:** combination of different substances (1); often sleeping pills or tranquilizers or party drugs (3); abuse for sexual offenses or robberies (1)	**Knockout drops:** increase acetylcholine, dopamine, and opioid peptides (4); impaired perception, consciousness, or amnesia (2)	**(1)** FNR-KO-Tropfen-aerzteinformation.pdf (w.d.); **(2)** Verba (2007); **(3)** Wikipedia authors (2022); **(4)** Stein (2003); **(5)** Zahavi (2016)	Survival	Mechanism of action: no; symptoms: yes
994	**Botulinum toxin:** toxin from the bacterium *Clostridium botulinum* (1); **Rabies virus:** family: Rhabdoviridae, genus: Lyssavirus (2); **exogenous insulins:** treatment of type 1 diabetes, later type 2 diabetes (5); increase of diabetic drugs and insulin therapy—an increase of homicides and suicides (6)	**Botulinum toxin:** inhibition of acetylcholine release at presynaptic cells (1); **rabies virus:** local replication (3); migration to CNS (3); replication (3); **exogenous insulins:** Insulin analogs (5); intoxication: hypoglycemia (5); irreparable brain damage, circulatory arrest (4)	**(1)** Dekant and Vamvakas (2010c, pp. 253f); **(2)** Series editing “RKI-Ratgeber” (2020); **(3)** Brunker and Mollentze (2018); **(4)** Datenblatt: Vergiftung-Antidiabetika (Insulin) (2021); **(5)** Seifert (2019h, i, pp. 244, 246f); **(6)** Bottinelli et al. (2020); **(7)** Jessen (2016)	Survival	Mechanism of action: no; symptoms: yes
956	**Knockout drops:** combination of different substances (3); often sleeping pills or tranquilizers or party drugs (5); abuse for sexual offenses or robberies (3); **GHB:** similar mechanism of action as neurotransmitter GABA (7); “liquid ecstasy” (6); **alcohol:** ethyl alcohol, ethanol, EtOH (1); narcotic drug (1)	**Knockout drops/GHB:** increase acetylcholine, dopamine, and opioid peptides (7); impaired perception, consciousness, or amnesia (4); **alcohol:** neurotoxic, hepato-, pancreatico-, and cardiotoxic (2)	**(1)** Hardman et al. (2001c, pp. 429ff); **(2)** Bützer (2016); **(3)** FNR-KO-Tropfen-Aerzteinformation.pdf (w.d.); (**4)** Verba (2007); **(5)** Wikipedia authors (2022); **(6)** Trendelenburg and Ströhle (2005); **(7)** Stein (2003); **(8)** Kren (2015)	**Knockout drops/GHB:** death and survival; **alcohol:** survival	Mechanism of action: no; symptoms: yes
917	**Exogenous insulins:** treatment of type 1 diabetes, for the advanced type 2 diabetes (2); increase of diabetic drugs and insulin therapy—> increase of homicides and suicides (3); **knock-out drops:** combination of different substances (5); often sleeping or sedative or party drugs (7); abuse for sexual offenses or robberies (5); **GHB:** similar mechanism of action as neurotransmitter GABA (9); “liquid ecstasy” (8); **amphetamines:** synthetic drugs (10); “speed” (10); **modafinil:** psychostimulant drug (11) **propranolol:** beta-blocker (12); **midazolam:** benzodiazepine (2); **cytarix:** fictitious	**Exogenous insulins:** Insulin analogs (2); intoxication: hypoglycemia (2); irreparable brain damage, circulatory arrest (1); **knockout drops/GHB:** increase of acetylcholine, dopamine, and opioid peptides (9); impairment of perception, consciousness or amnesia (6); **amphetamines:** release of norepinephrine and dopamine (10); centrally stimulating, euphoric (10); **modafinil:** sympathomimetic (11); inhibition of dopamine reuptake (11); **propranolol:** inhibition of epinephrine and norepinephrine effects, blocking of ß-receptors (12); **midazolam:** allosteric GABA_A_-receptor modulation (2)	**(1)** Datenblatt: Vergiftung-Antidiabetika (Insulin) (2021); **(2)** Seifert (2019h, i, j, pp. 244, 246f, 312); **(3)** Bottinelli et al. (2020); **(4)** FNR-KO-Tropfen-Aerzteinformation.pdf (w.d.); (**5)** Verba (2007); **(6)** Wikipedia authors (2022); **(7)** Trendelenburg and Ströhle (2005); **(8)** Stein (2003); **(9)** Dekant and Vamvakas **(**2010a, pp. 235f); **(10)** Walliczek-Dworschak (2019); **(11)** Hardman et al. (2001a, p. 249); **(12)** Jauch (2014)	**Exogenous insulins:** death; **knockout drops/GHB:** death; **amphetamine, modafinil, propranolol, midazolam:** mentioned only in passing	Mechanism of action: no; symptoms: yes
905	**Barbiturates:** sedatives, hypnotics, and injectable narcotics (1); **carbon monoxide (CO):** gas (2); source: combustion processes (transport, industry, engines) (2)	**Barbiturates:** enhanced inhibition of excitatory neurotransmission, enhancement of GABAergic inhibitory transmission (1); **CO:** forms a complex with hemoglobin (2); blocks oxygen binding site (2)	**(1)** Seifert (2019j, k, pp. 310, 328); **(2)** Hardman et al. (2001e, pp. 1880ff); **(3)** Fischer (2014)	**Barbiturates:** death; **CO:** death, survival	Mechanism of action: no; symptoms: yes
765	**Zytrex 3:** fictitious	No mechanism because fictitious	No literature because fictitious; **(1)** Baxmeyer (2010)	Death	Mechanism of action: no; symptoms: yes
750	**Barbiturates:** sedatives, hypnotics, and injectable narcotics (1); **benzodiazepines:** frequently used drug group (1**)**; **methaqualone (2-methyl-3-o-tolyl-4 (3H)-quinazolinone):** sedative and hypnotic (2); **diphenhydramine:** first-generation H1 receptor antagonist (1); **etodoxizine**: fictitious	**Barbiturates:** enhanced inhibition of excitatory neurotransmission, potentiation of GABAergic inhibitory transmission (1); **benzodiazepines:** potentiation of GABAergic inhibition (1); anxiolytic, sedative-hypnotic, muscle relaxant, and antiepileptic (1); **methaqualone:** positive allosteric GABA_A_ receptor modulator (2); **diphenhydramine:** sedation, antiallergic, itch relief (1)	**(1)** Seifert (2019c, j, k, pp. 95, 310ff, 328); **(2)** Ionescu-Pioggia et al. (1988); **(3)** Moore (2009)	Death	Mechanism of action: no; symptoms: yes
708	**Carbon monoxide (CO):** gas (1); source: combustion processes (transport, industry, engines) (1)	**CO:** forms a complex with hemoglobin (1); blocks oxygen binding site (1)	**(1)** Hardman et al. (2001e, pp. 1880ff); **(2)** Pfeiffer (2008)	Death	Mechanism of action: no; symptoms: yes
699	**Barbiturates:** sedatives, hypnotics, and injectable narcotics (1); **Thelotal:** fictitious barbiturate	**Barbiturates:** enhanced inhibition of excitatory neurotransmission, enhancement of GABAergic inhibitory transmission (1)	**(1)** Seifert (2019j, k, pp. 310ff, 328); **(2)** Stelzer (2008)	Death	Mechanism of action: no; Symptoms: no
637	**Aconitine:** from *Aconitum napellus* (aconite) (1); in tuber and seed (2)	**Aconitine:** direct activation and impairing closing of voltage-gated sodium channels (3)	**(1)** Dekant and Vamvakas (2010b, pp. 247f); **(2)** Aktories et al. (2013h, p. 1070); **(3)** Chan (2009); **(4)** Garde (2006)	Death	Mechanism of action: no; symptoms: yes
545	**Trimethanoctulol phenyl carbon hydride:** fictitious	No mechanism because fictitious	No literature because fictitious; **(1)** Jauch (2003)	Death	Mechanism of action: no; symptoms: yes
542	**Synthetic poisons:** non-natural occurrence, artificial production (1)	No mechanism because no exact substance is mentioned	**(1)** Auwärter et al. (2012); **(2)** Emmerich (2003)	Death	Mechanism of action: no; symptoms: yes
505	**Digitalis:** family Plantaginaceae (1); active ingredients: digitoxin, digoxin (1); **exhaust gases:** often carbon monoxide (CO), carbon dioxide (CO_2_), and/or nitrogen oxides (NO_*x*_) (2)	**Digitalis:** inhibition of Na^+^/K^+^-ATPase (3); increase in intracellular calcium concentration (3); positive inotropic and negative dromotropic (3)	**(1)** Aktories et al. (2013c, p. 414); **(2)** Ziegler et al. (2014); **(3)** Seifert (2019g, p. 212); **(4)** Agthe (2002)	Death	Mechanism of action: no; symptoms: yes
458	**Botulinum toxin:** toxin from the bacterium *Clostridium botulinum* (1)	**Botulinum toxin:** presynaptic inhibition of acetylcholine release (1)	**(1)** Dekant and Vamvakas (2010c, pp. 253f); **(2)** Heidelbach (2000b)	Death	Mechanism of action: no; symptoms: yes
447	**Strychnine:** from seeds of *Strychnos nux-vomica* (poison-nut tree) (1)	**Strychnine:** blocking of glycine receptors (1)	**(1)** Hardman et al. (2001e, p. 1894); **(2)** Fischer (2000)	Death	Mechanism of action: no; symptoms: yes
437	**Potassium cyanide (KCN):** cyanide (1)	**KCN:** complex formation—> blocking of cytochrome oxidases (1); interruption of the respiratory chain (1)	**(1)** Hardman et al. (2001e, p. 1893); **(2)** Heidelbach (2000a)	Death	Mechanism of action: yes; symptoms: yes
397	**Carbon monoxide (CO):** gas (1); source: combustion processes (transport, industry, engines) (1)	**CO:** complex formation with hemoglobin (1); blocks oxygen binding site (1)	**(1)** Hardman et al. (2001e, pp. 1880ff); **(2)** Freundner (1998)	Death	Mechanism of action: no; symptoms: yes
366	**Cyan nitrate:** fictitious	No mechanism because fictitious	No literature because fictitious; **(1)** Vogel (1997)	Death and survival	Mechanism of action: no; Symptoms: yes
364	**Kilat:** fictitious; **Pervitin:** psychoanaleptic compound (1); similar to ephedrine (1); **Valocordin:** INN: doxylamine or diazepam (2,3); **Tilur:** INN: acemetacin (4); COX inhibitor (5)	**Pervitin:** affects mental and physical behavior (1); **Valcordin:** doxylamine: first-generation H_1_ receptor antagonist (6); CNS depressant effect (6); diazepam: anxiolytic, sedative-hypnotic, muscle relaxant, and antiepileptic (7); **Tilur:** inhibition of prostaglandin formation (5)	**(1)** Bonhoff (2013); **(2)** Gmbh (2022); (**3)** Editorial Gelbe Liste Pharmindex (2022); (**4)** Gmbh (w.d.); **(5)** Editorial Gelbe Liste Pharmindex (2016); **(6)** Kahle (2020); **(7)** Seifert (2019j, p. 312); **(8)** Panzer (1997)	Kilat: death; Pervitin, Valcordin, Tilur: mentioned only in passing	Mechanism of action: yes; symptoms: no
342	**Asbestos:** fibrous, naturally occurring minerals (2); **dibenzodioxin:** most potent representative: 2,3,7,8-tetrachlorodibenzodioxin (TCDD) (3)	**Asbestos:** mechanical irritation, damage to bronchial system and lungs (1); phagocytosis, apoptosis, or necrosis (1); carcinogenic (1); **dibenzodioxin:** tumor-promoting, toxic effect mediated by Ah receptor: regulation of protein expression for carcinogenic hydrocarbons (3)	**(1)** Marczynyki et al. (1999); **(2)** Seidel et al. (2007); **(3)** Aktories et al. (2013d, g, pp. 1005f, 1055f); **(4)** Schlotterbeck (1996)	Death and survival	Mechanism of action: no; symptoms: yes
302	**Laposan 31:** fictitious	No mechanism because fictitious	No literature because fictitious; **(1)** Haffter (1995)	Death	Mechanism of action: no; symptoms: yes
278	**Heroin:** also called diacetylmorphine (1); rapid development of tolerance (1)	**Heroin:** strong agonist at opioid receptors (1)	**(1)** Dekant, Vamvakas (2010a, p. 242); **(2)** Bannert (1993)	Death and survival	Mechanism of action: no; Symptoms: yes
201	**Knockout drops:** combination of different substances (1); often sleeping pills or tranquilizers or party drugs (2); abuse for sexual offenses or robberies (1); **heroin:** also called diacetylmorphine (4); rapid development of tolerance (4)	**Knockout drops:** increase in acetylcholine, dopamine, and opioid peptides (5); impairment of perception, consciousness, or amnesia (3); **heroin:** strong agonist at opioid receptors (4)	**(1)** FNR-KO-Tropfen-Aerzteinformation.pdf (w.d.); **(2)** Wikipedia authors (2022); **(3)** Verba (2007); **(4)** Dekant and Vamvakas (2010a, p. 242); Stein (2003); **(6)** Blumenberg (1988)	**Knockout drops:** death and survival; h**eroin:** death and survival	Mechanism of action: no; symptoms: yes
84	**Exhaust gases:** often carbon monoxide (CO), carbon dioxide (CO_2_) and/or nitrogen oxides (NO_x_) (1,2); **carbon monoxide (CO):** gas (1); source: combustion processes (transport, industry, engines) (1)	**CO:** complex formation with hemoglobin (1); blocks oxygen binding site (1)	**(1)** Hardman et al. (2001e, pp. 1880ff); **(2)** Ziegler et al. (2014); **(3)** Neureuther (1978)	Death	Mechanism of action: no; symptoms: yes
82	**Notin-Gamma-Corbin:** fictitious	No mechanism because fictitious	No literature because fictitious; **(1)** Gies (1977)	Death	Mechanism of action: no; symptoms: yes
76	**Potassium cyanide (KCN):** cyanide (1)	**KCN:** complex formation—> blocking of cytochrome oxidases (1); interruption of the respiratory chain (1)	**(1)** Hardman et al. (2001e, p. 1893); **(2)** Gräwert (1977)	Death and survival	Mechanism of action: no; symptoms: no
75	No substance is mentioned	No mechanism because no substance is mentioned	No literature because no substance is mentioned; **(1)** Staudte (1977)	Death	Mechanism of action: no; symptoms: yes
50	**Potassium cyanide (KCN):** cyanide (1)	**KCN:** complex formation—> blocking of cytochrome oxidases (1); interruption of the respiratory chain (1)	**(1)** Hardman et al. (2001e, p. 1893); **(2)** Wirth (1975)	Death	Mechanism of action: no; symptoms: yes
39	**Carbon monoxide (CO):** gas (1); source: combustion processes (transport, industry, engines) (1)	**CO:** complex formation with hemoglobin (1); blocks oxygen binding site (1)	**(1)** Hardman et al. (2001e, pp. 1880ff); **(2)** Becker (1974)	Survival	Mechanism of action: no; symptoms: yes

Numbers in parentheses refer to references listed in column 4. Complete references are provided in the reference list. Relevant poisons and compounds are highlighted in boldface. —> indicates a consequence of compound action

### Comparison with real poisoning data

Real poisoning data were kindly provided to us by the Giftinformationszentrum (Poison Information Center) Mainz, Germany (https://www.unimedizin-mainz.de/giz/uebersicht.html, accessed 28 March 2022). These data were compared with the data from *Tatort*. From these data, information on substance categories, poisoning outcomes, application, and etiology was filtered. These data were used to establish an annual comparison of real-life poisonings. Since data on real poisoning outcomes were also available for at least some years, the relevant data were collected as well (GIZ-Mainz, Germany 2000, 2001, 2002, 2003, 2004, 2005, 2006, 2007, 2008, 2011, 2022). It should be noted, however, that available datasets on real poisonings are incomplete.

### Gender analysis

To examine gender roles in poisonings in *Tatort* as well as in reality, the total number of offenders and victims in the crime scene was first determined. Then, the number of men and women was determined for offenders and victims. With these data, a diagram showing the gender distribution of offenders and victims at the crime scene was generated. Next, the figures from GIZ from 2000 to 2008 + 2011 were analyzed. Here, the figures refer to general poisonings only. This information was used to generate a chart of the genders in general poisonings. Last, real numbers from a series of criminal poisonings (Fuhrmeister 2005) were used, and a chart was also created.

## Results

### Analysis of poisonings in all episodes

**Fig. 2:**
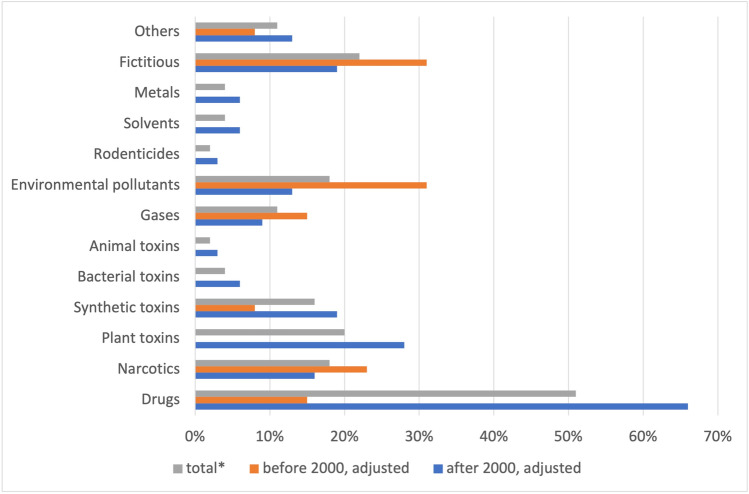
Substance categories involved in poisoning. Shown is a bar chart, where the total value (*percentage value of all episodes) is grey, the value before 2000 is orange, and the value after 2000 is blue. Table 1 provides details on the poisoning cases

Figure 2 summarizes the substance categories involved in poisonings in the analyzed *Tatort* episodes expressed in adjusted percentage. The adjustment had to be made because there are more substances involved in poisonings than the episodes analyzed (Table 1). Drugs are involved in 51% of all poisonings (15% before 2000 and 66% after 2000). For environmental pollutants, the value is 18% (31% before 2000 and 13% after 2000). Fictitious substances amount to 22% of all poisonings (31% before 2000 and 19% after 2000).

**Fig. 3:**
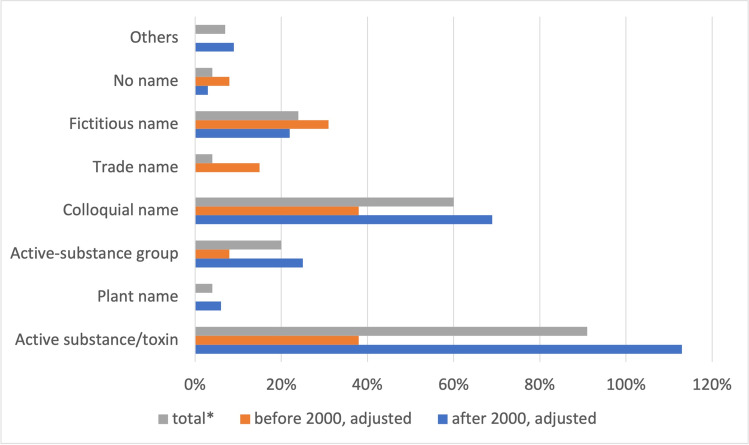
Naming of substances involved in poisoning. Shown is a bar chart, where the total value (*percentage value of all episodes) is grey, the value before 2000 is orange, and the value after 2000 is blue

Substances/toxins are named in 91% of all episodes (38% before 2000, 113% (more poisonings than episodes!) after 2000) (Fig. 3). The colloquial name is given in 60% of all cases (38% before 2000 and 69% after 2000). Substances without a name amount to 4% (8% before 2000 and 3% after 2000).

**Fig. 4:**
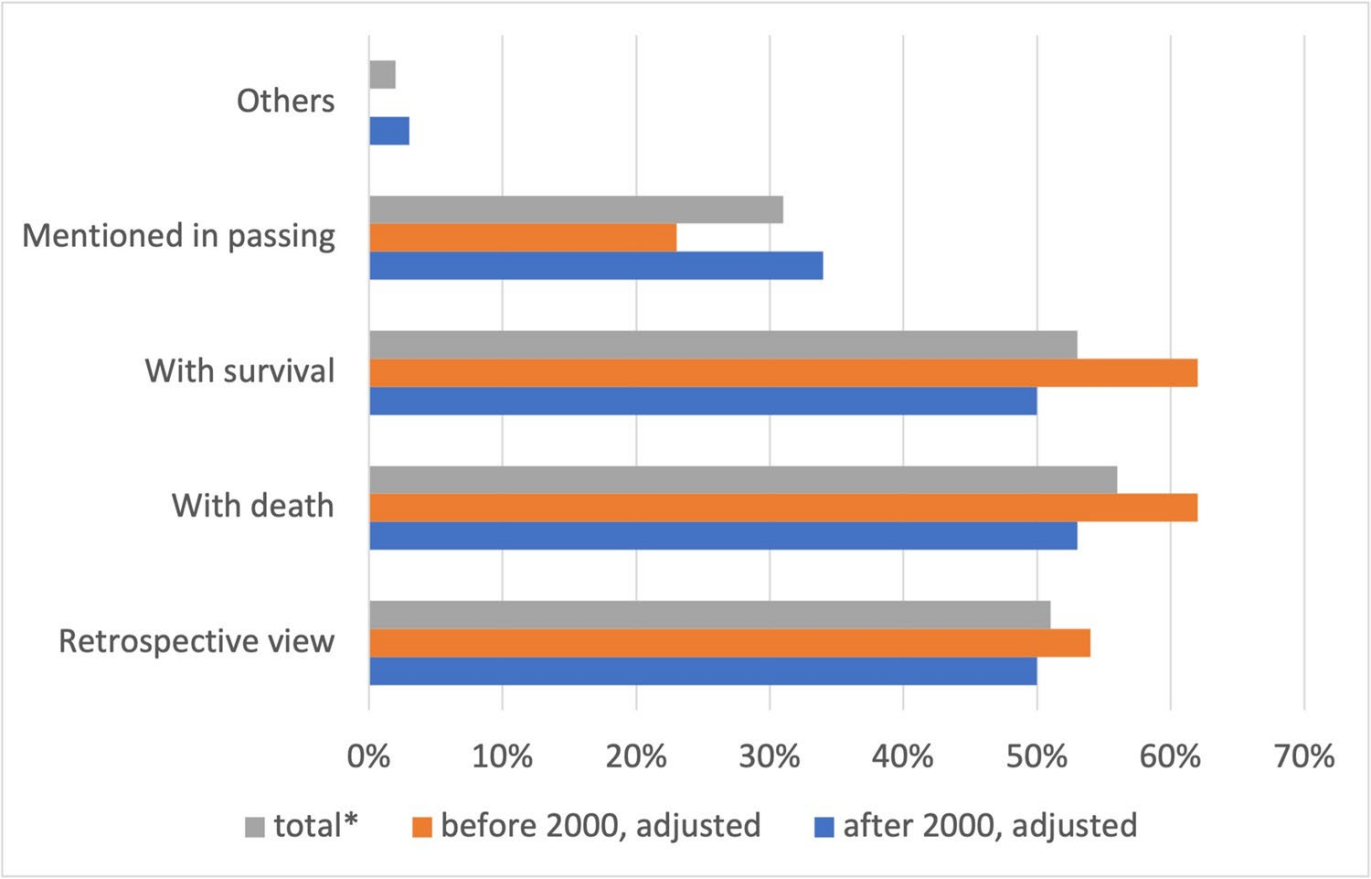
Presentation and outcome of poisoning. Shown is a bar chart, where the total value (*percentage value of all episodes) is grey, the value before 2000 is orange, and the value after 2000 is blue

Death resulting from poisoning in *Tatort* occurs in 56% of all poisonings (62% before 2000 and 53% after 2000) (Fig. 4). The value for survival is 53% (62% before 2000 and 50% after 2000). The retrospective view of poisoning accounts for 51% of all cases (54% before 2000 and 50% after 2000).

**Fig. 5:**
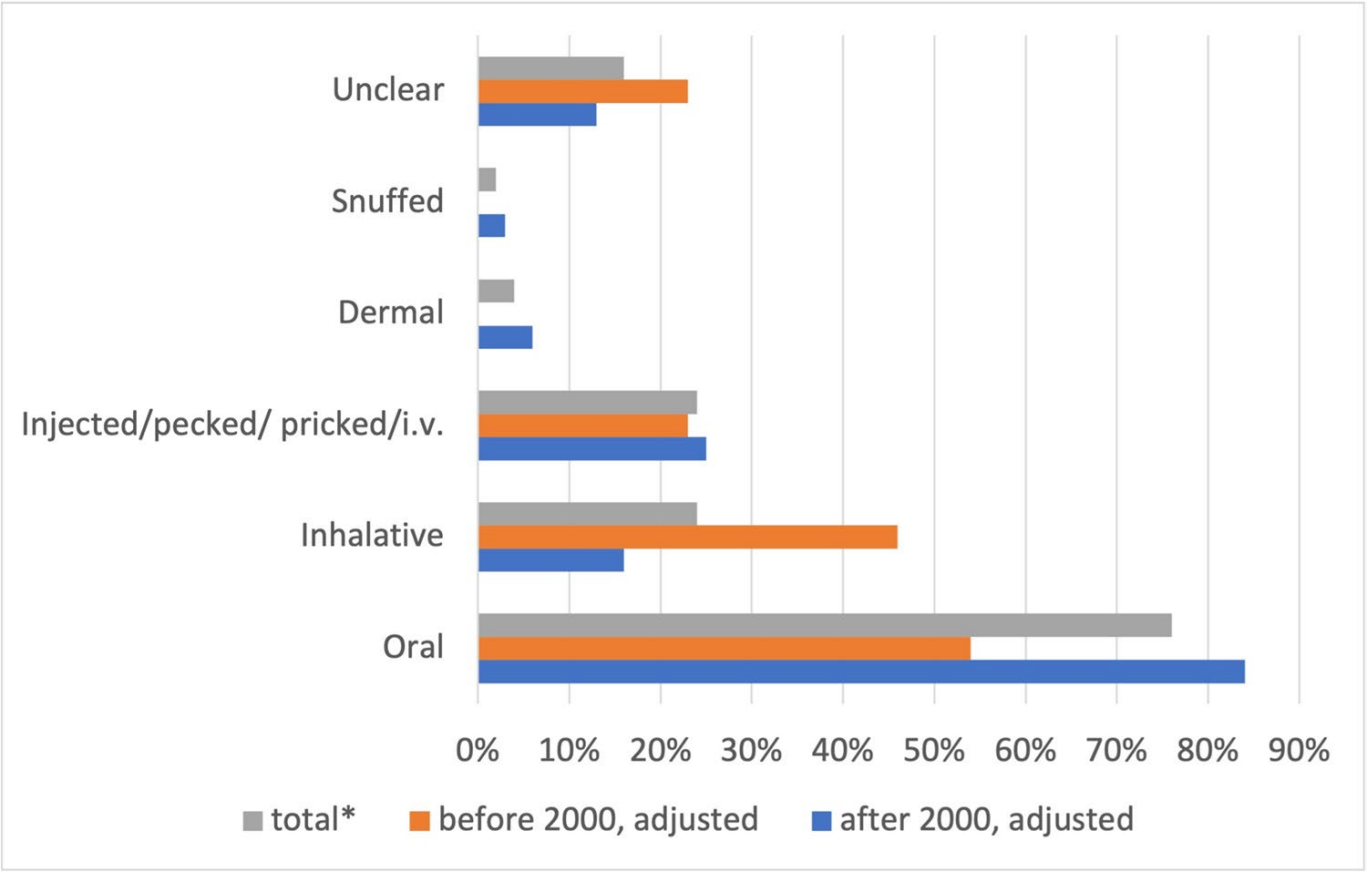
Application of poisons. Shown is a bar chart, where the total value (*percentage value of all episodes) is grey, the value before 2000 is orange, and the value after 2000 is blue

Oral application of substances occurred in 76% of all poisonings (54% before 2000 and 84% after 2000) (Fig. 5). Inhalation occurred in 24% of all poisonings (46% before 2000 and 16% after 2000).

**Fig. 6:**
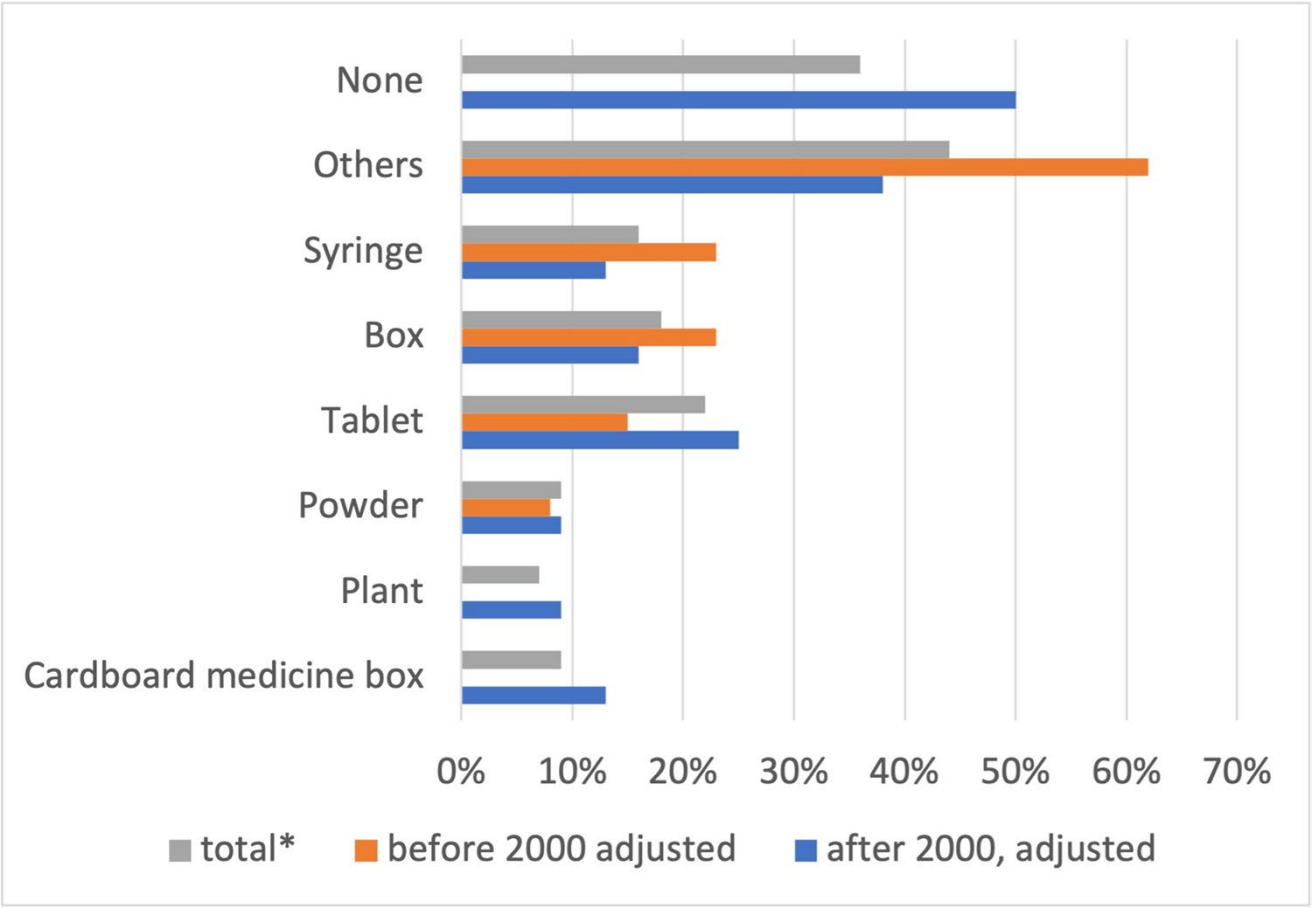
Substance presentations involved in poisoning. Shown in a bar chart, where the total value (*percentage value of all episodes) is grey, the value before 2000 is orange, and the value after 2000 is blue

Substances are presented in the form of tablets in 22% of all poisonings (15% before 2000 and 25% after 2000) (Fig. 6). Substances that are dealt with in context with poisoning in *Tatort* but which are not further presented account for a total of 36% (0% before 2000 and 50% after 2000). Forty-four percent of the substances (62% before 2000 and 38% after 2000) belong to the “others” category (e.g., the frog for frog’s toxins or the drink for knockout drops).

**Fig. 7:**
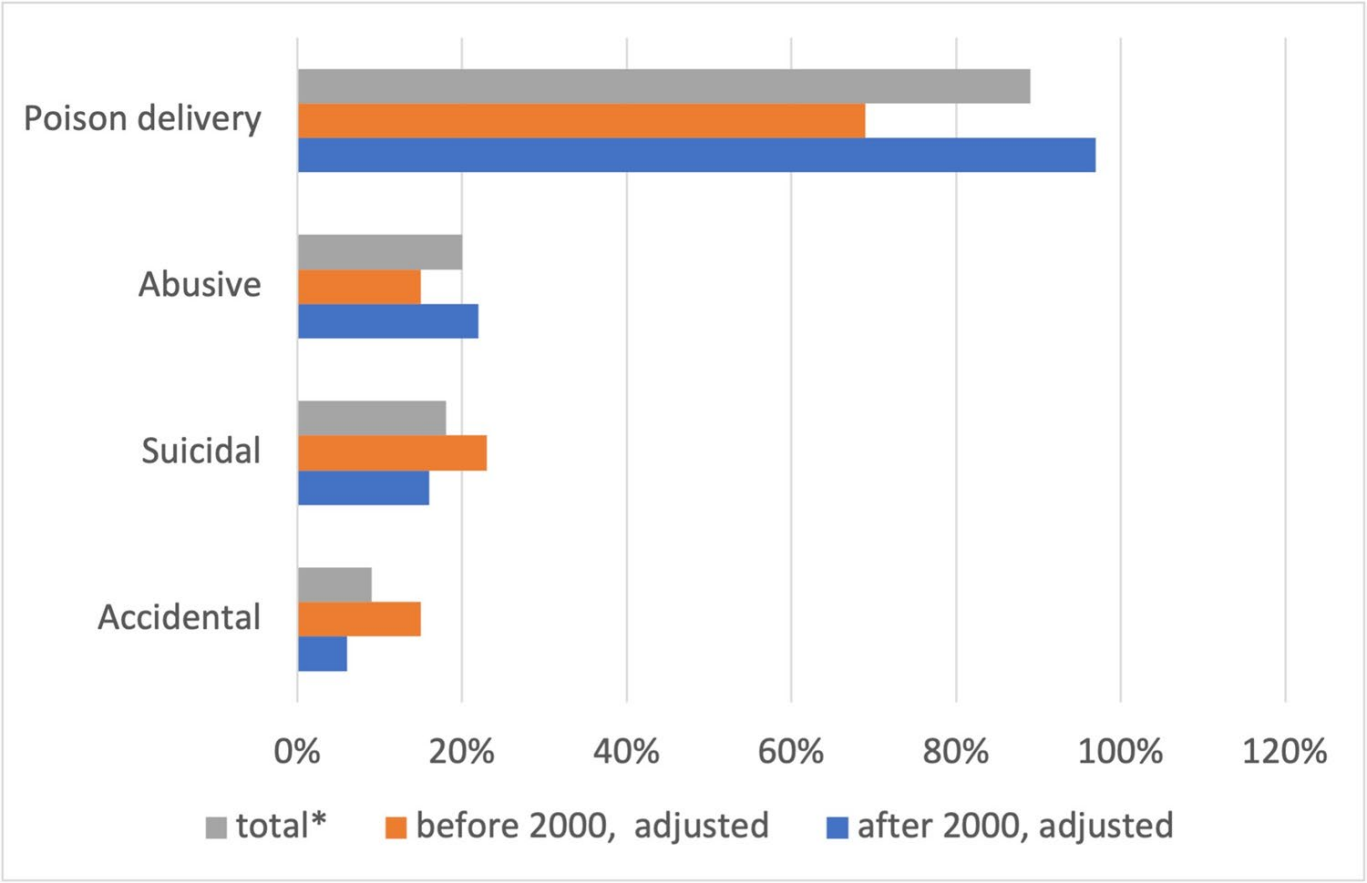
Etiology of poisoning. Shown is a bar chart, where the total value (*percentage value of all episodes) is grey, the value before 2000 is orange, and the value after 2000 is blue

External poison delivery accounts for 89% of all poisonings (69% before 2000 and 97% after 2000) (Fig. 7). Accidental poisoning accounts for 9% of all cases (15% before 2000 and 6% after 2000).

**Fig. 8:**
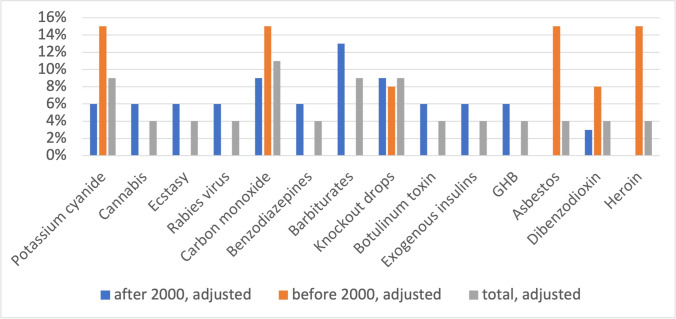
Substances repeatedly involved in poisoning. Shown in a bar chart, where the value before 2000 is orange, the value after 2000 is blue and the total value (percentage value of all episodes) is grey

Before 2000, potassium cyanide, carbon monoxide, asbestos, and heroin are the most frequent causes of poisoning (Fig. 8). After 2000, barbiturates, knockout drops, and carbon monoxide are the most common causes of poisoning.

### Episode ratings

**Fig. 9:**
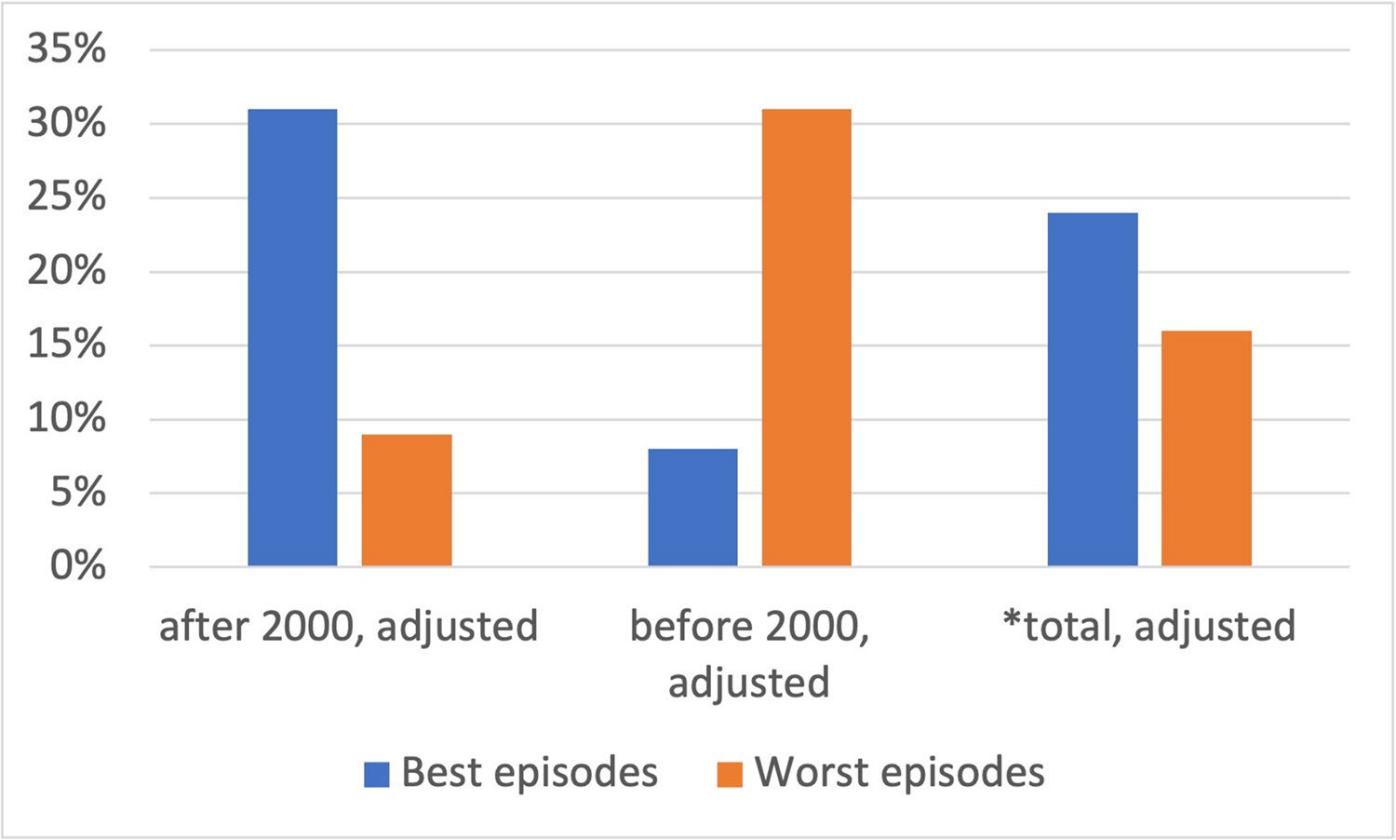
Ratings of episodes showing poisoning. Shown is a bar chart, where the value for the best episodes is blue and that for the weakest episodes is orange

Eight percent of all episodes before 2000 were placed into the best category, 31% of all episodes after 2000, and overall, the best episodes account for 24% of all episodes analyzed (Fig. 9). The value of the lowest rated episodes is 31% before 2000 and 9% after 2000. The weakest episodes account for 16% of all 45 episodes.

### Missing information

**Fig. 10:**
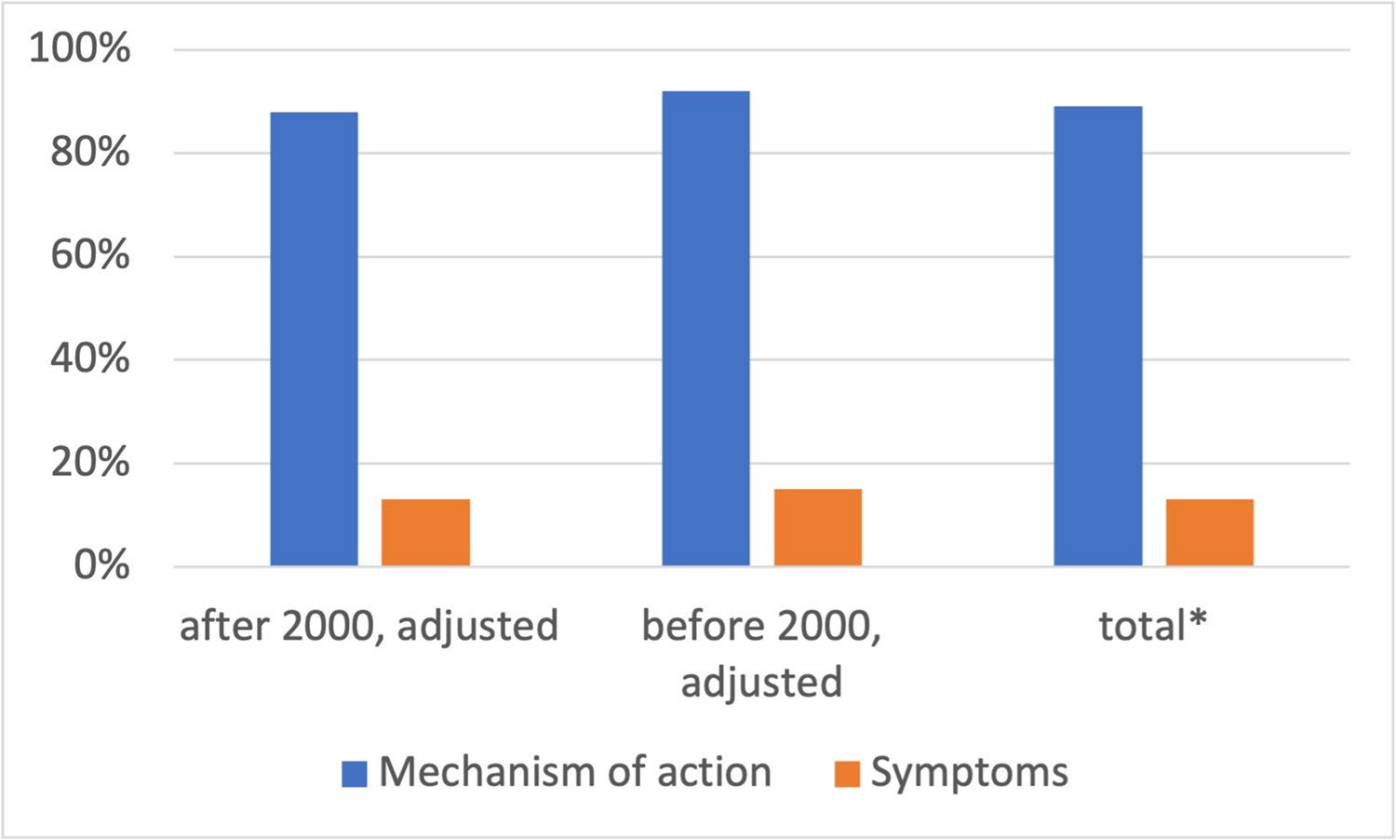
Missing information on mechanism of action and symptoms in poisonings. Shown is a bar chart, where the value for the mechanism of action is blue and that for symptoms is orange

In 89% of all episodes, the mechanism of action of poisoning (etiology) is missing (92% before 2000 and 88% after 2000) (Fig. 10). Symptoms are not represented in 13% of all episodes overall (15% before 2000 and 13% after 2000).

### Fiction–reality comparison

**Fig. 11:**
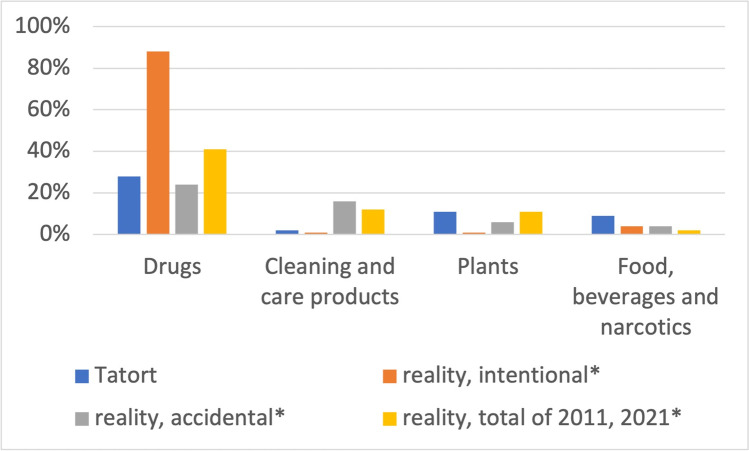
Fiction-reality comparison of substance categories involved in poisoning, *mean real-life values for intentional and accidental poisonings from 2000 to 2008, and *mean real-life values for all poisonings in 2011 + 2021. Shown is a bar graph, with *Tatort* values in blue, real-life accidental poisonings in grey, real-life intentional poisonings in orange, and all poisonings in 2011 + 2021 in yellow

When comparing the substance categories in *Tatort* with reality, the percentage for drugs is 28% for the crime scene, 88% is the mean for intentional poisonings with drugs from 2000 to 2008, 24% for accidental, and 41% for all poisonings from 2011 to 2021 (Fig. 11).

**Fig. 12:**
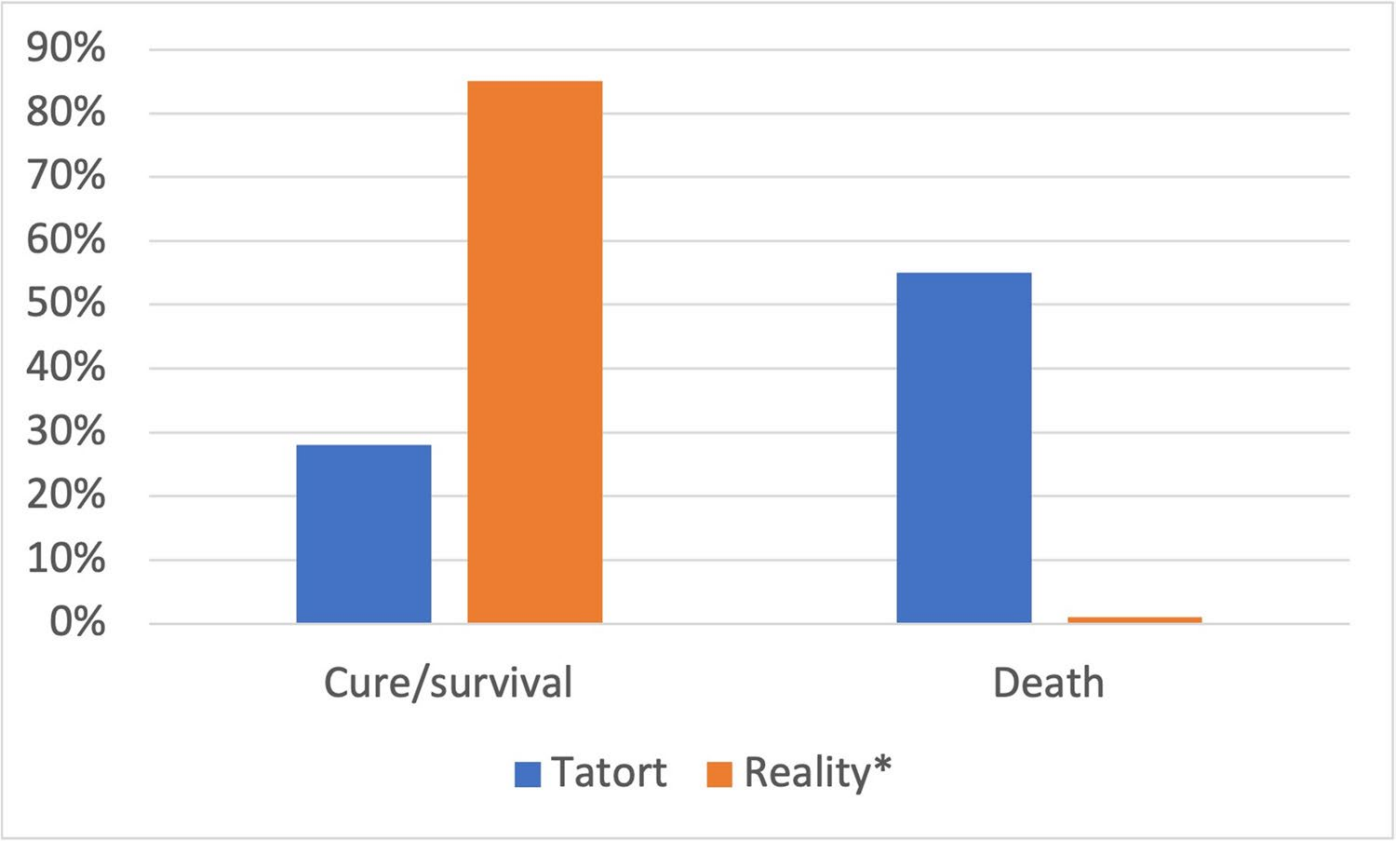
Fiction-reality comparison of poisoning outcomes using total values from *Tatort* and *mean real-life values from 2000 to 2007. Shown is a bar chart, where the value for *Tatort* is blue and that for reality is orange

When comparing poisoning outcomes in *Tatort* with reality, *Tatort* has a cure/survival rate of 28%, whereas, in reality, the mean value accounts for 85% (Fig. 12). The death rate in *Tatort* is 55%; in reality, the death rate is 1%.

**Fig. 13:**
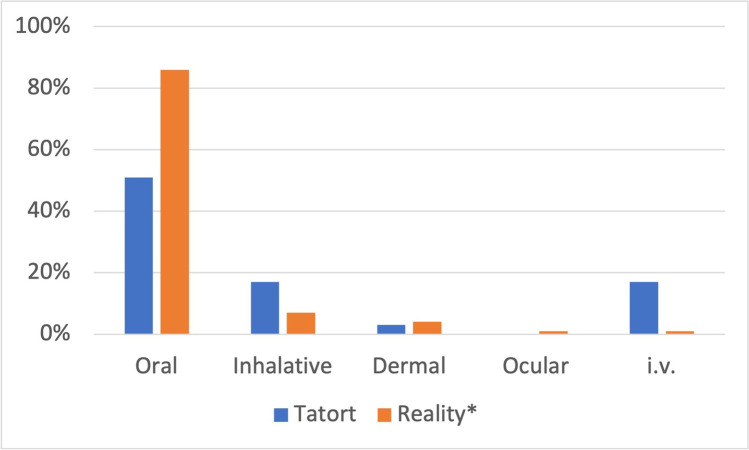
Fiction–reality comparison of the application using the total values from *Tatort* and the *mean reality values from 2000 to 2008 and 2011. Shown is a bar chart, with values for *Tatort* in blue and values for reality in orange

The oral route accounts for 51% of all poisoning cases in *Tatort*, whereas the mean real-life numbers amount to 86% (Fig. 13). Inhalation in *Tatort* occurs in 17% of all cases, and in reality, in 7%. Intravenous (IV) administration of substances in *Tatort* occurs in 17% of all cases and in 1% of all cases in reality.

**Fig. 14:**
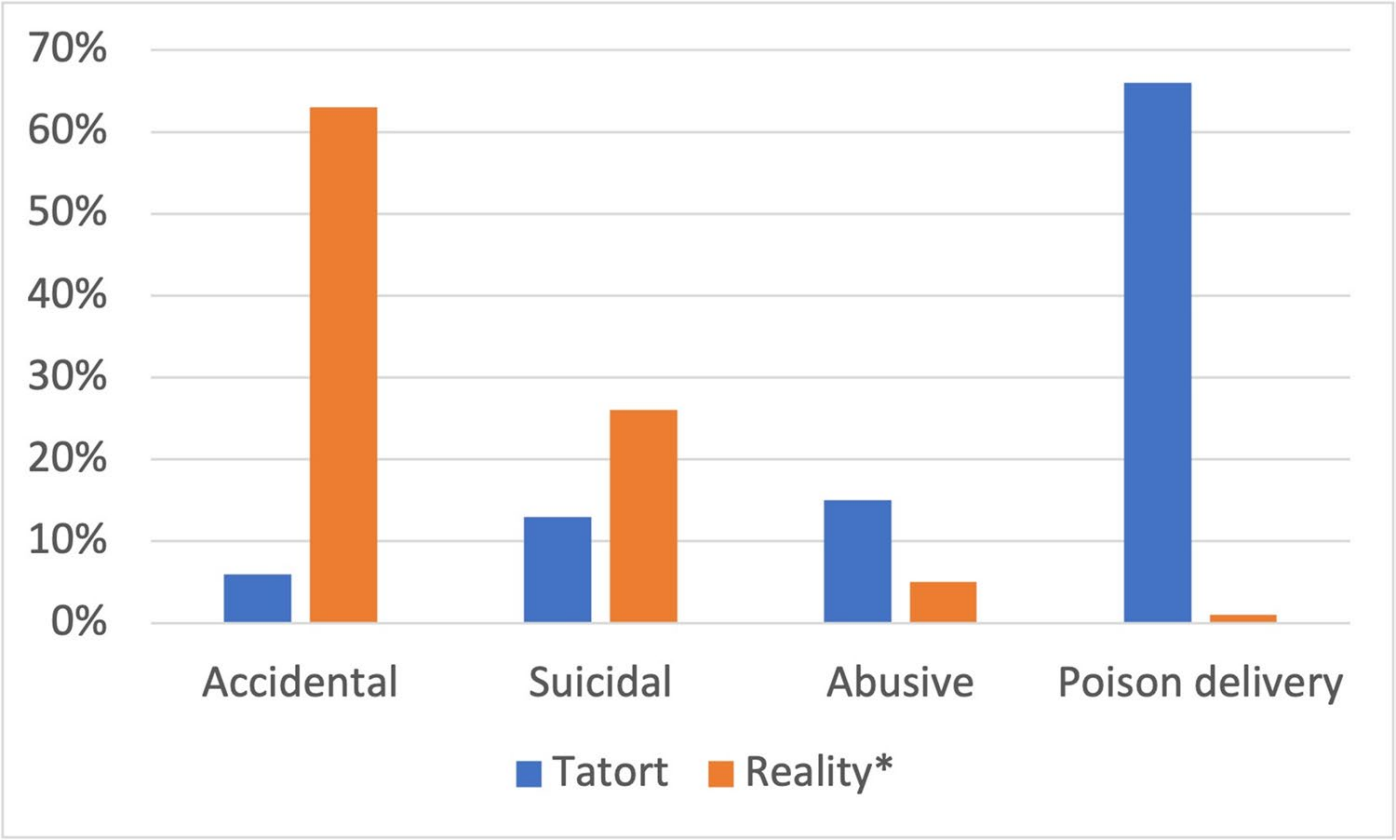
Fiction-reality comparison of etiology using total values from *Tatort* and *mean reality values from 2000 to 2008 and 2011. Shown is a bar chart, where the values for *Tatort* are blue and those for reality are orange

Accidental poisoning in *Tatort* accounts for just 6% of all cases, whereas the real value is 63% (Fig. 14). Poison delivery accounts for 66% of all cases in *Tatort* and 1% in reality.

### Gender analysis

**Fig. 15:**
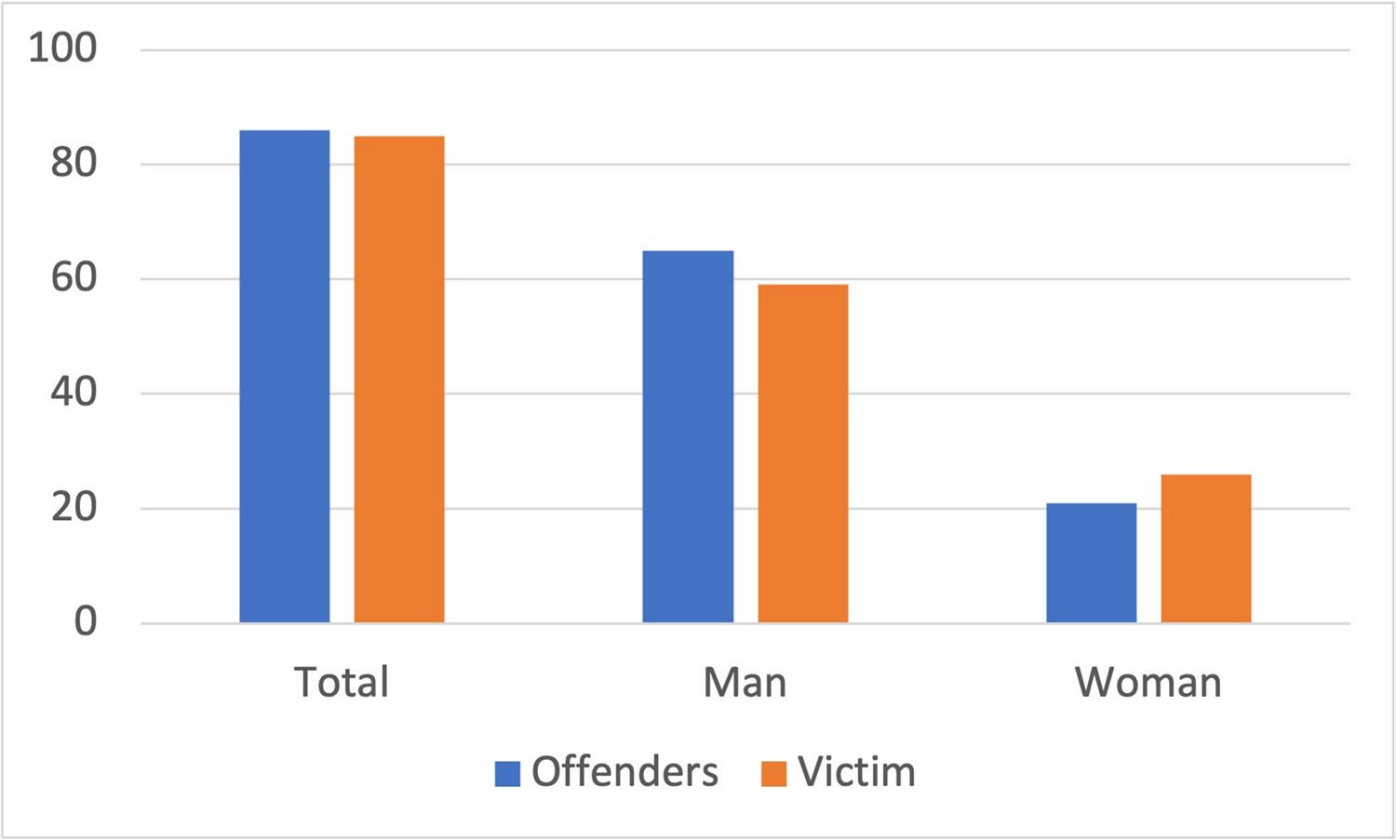
Gender analysis from *Tatort*. Shown is a bar chart, where the values for offenders are blue and those for victims are orange

In total, there are 86 offenders and 85 victims in the analyzed *Tatort* episodes (Fig. 15). This is because, in one episode, two perpetrators killed one victim together. Of the total number of offenders, 65 are males and 21 are females. There are 59 male victims and 26 female victims.

**Fig. 16:**
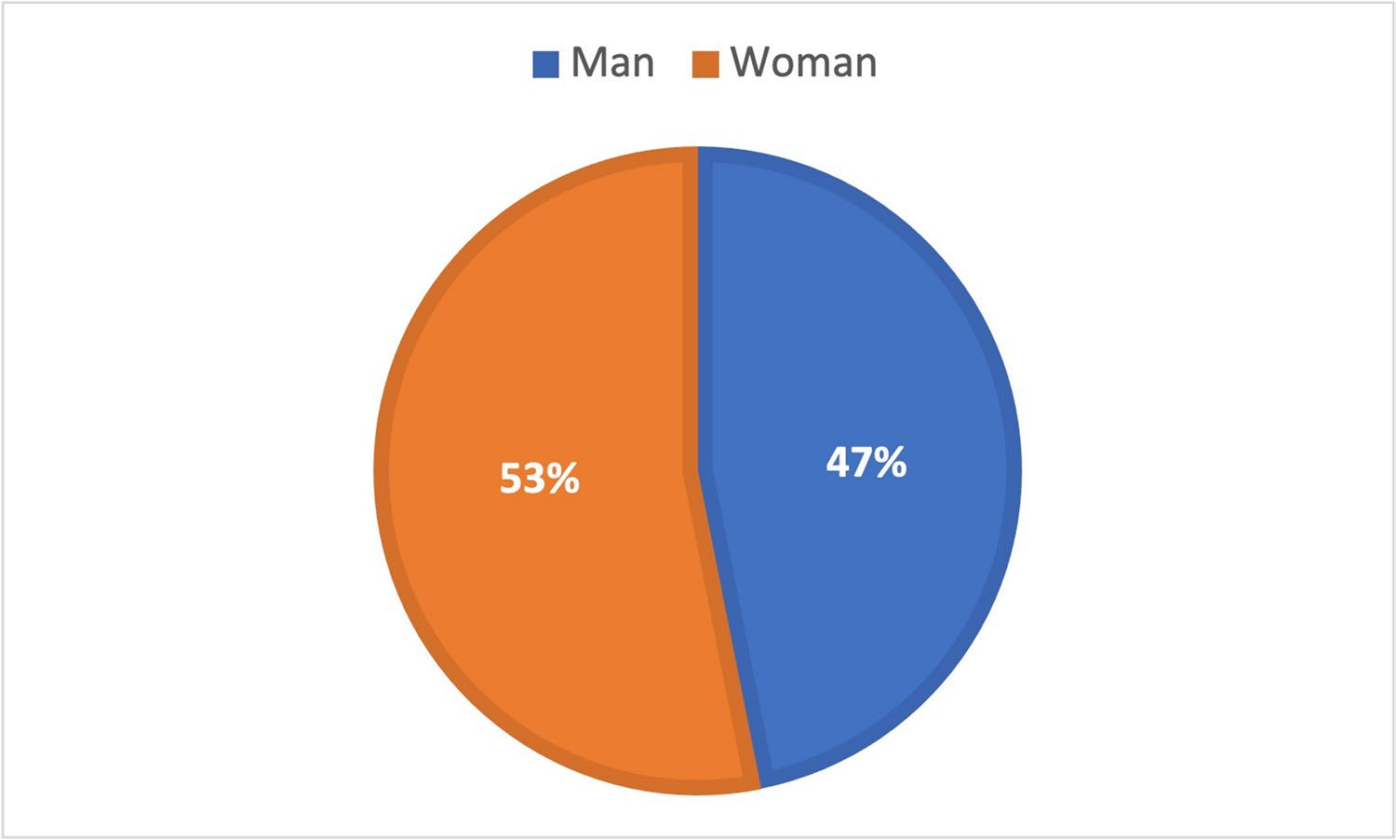
Gender in general poisoning with data from GIZ. Shown is a pie chart, where the value for men is blue and the one for women is orange

The mean value from the numbers of poisoning victims from GIZ from 2000 to 2008 + 2011 yields 47% for men and 53% for women (Fig. 16).

**Fig. 17:**
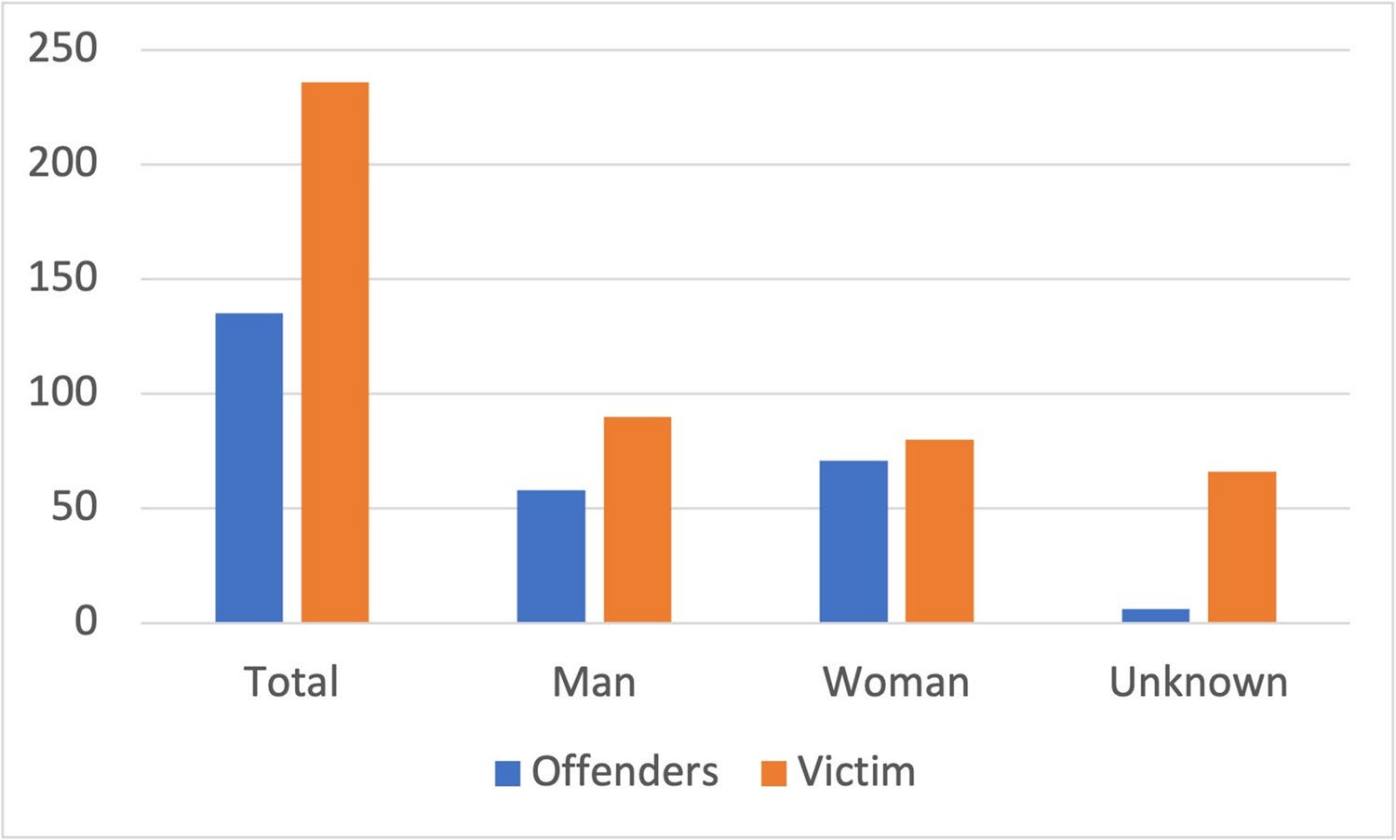
Gender in criminal cases involving poisoning. Shown is a bar chart, where the value for offenders are blue and those for victims are orange. Data are based on Fuhrmeister (2005)

In a representative study on true crime cases involving poisoning, the total number of offenders was 135 and of victims was 236 (Fig. 17) (Fuhrmeister 2005). There are 58 male and 71 female offenders. In 6 cases, the gender was unknown. Among the victims, there were 90 males and 80 females. In 66 cases, the gender was unknown (Fig. 17).

## Discussion

### Analysis of the different dimensions of poisoning

Plant-derived, bacterial and animal toxins, rodenticides, solvents, metals, and other substances (nanobots, rabies virus, alcohol) play an increasing role in the new episodes of *Tatort* (Fig. 2). Conversely, narcotics, gases, environmental pollutants, and fictitious substances play a greater role in the older episodes. Some categories, such as plant, bacterial and animal toxins, rodenticides, solvents, and metals, never occurred in the older episodes. This reflects an increasing diversity of substances in the *Tatort* episodes over the years.

In the new episodes, substances are mainly referred to as active ingredient/toxin, plant name, active-ingredient group, and colloquial name (Fig. 3). The old episodes preferentially use trade names, fictitious names, or there is no name at all. Fictitious names and no substance names result in a poorer understanding of poisoning in the old episodes. In addition, the use of trade names impedes understanding and suggests that only this particular trade product is toxic.

A retrospective view of poisoning, i.e., an event of poisoning that occurs at the beginning of an episode, is slightly more frequent in the old episodes (Fig. 4), as is poisoning with death or with survival. Poisonings mentioned in passing occur more frequently in new episodes (Fig. 4). The occurrence of incidental mentions in new episodes can also explain the increasing substance diversity. Cannabis or ecstasy, for example, are often mentioned in side stories.

Oral, injected/pricked/stung/IV, dermal, and snorted administration routes can be assigned to the new episodes, whereas inhalation and unclear routes of administration occur in the old episodes (Fig. 5). Thus, over the years, more emphasis has been placed on how poisons are administered, and which different routes of administration exist.

In the new episodes, substances are not only named but are also presented by means of cardboard medicine boxes, plants, powders, and tablets (Fig. 6). However, these presentations are incomplete. In 50% of the new episodes, only the substance is mentioned, but no additional presentation takes place. In the old episodes, the substance is preferentially presented in boxes, syringes, or by other means (drinks, poison dart frog, etc.). Abusive and intentional poisoning dominate in the new episodes, whereas accidental and suicidal poisonings dominate in the old episodes (Fig. 7).

Among the substances that are used in several *Tatort* episodes, balanced use of knockout drops in the old and new episodes is observed; potassium cyanide and carbon monoxide are thematized slightly more frequently before 2000 (Fig. 8). Heroin and asbestos are exclusively shown in the old episodes. Since asbestos fell into disrepute in the 1990s and its production and use have been banned in Germany since 31 October 1993 (https://www.umweltbundesamt.de/themen/gesundheit/umwelteinfluesse-auf-den-menschen/chemische-stoffe/asbest, accessed 22 March 2022), it was a hot topic, especially in the pre-2000 *Tatort* episodes. In the 1990s, the topic of illicit drug use was often thought of only in terms of heroin abuse, since it was one of the most widespread drugs in many countries (Beubler et al. 2006). However, changes have occurred over the years (Fischer et al. 2008). In some countries, the number of heroin users is stable and the incidence is decreasing (Beubler et al. 2006). In other countries, there is even a decrease (Fischer et al. 2008; Teesson et al. 2006). At the same time, polydrug use and stimulant use are on the rise (Beubler et al. 2006). These dynamic changes in the drug market, with the declining use of heroin, explain the increased representation of heroin in the old episodes. Since the use of stimulants such as ecstasy has increased in recent years and thus only then became a topical issue, it is understandable why ecstasy is only shown in the new episodes (Beubler et al. 2006). The fact that cases of intoxication with cannabis occur only in the new episodes may be due to the fact that the discovery of cannabinoid receptors and associated new possibilities for medicine, in the 1990s, caused significant public interest (Zuardi 2006). From these examples, we can see that the change in the substances used in *Tatort* is driven by current societal and medical topics.

### Missing information

Strikingly, the mechanism of action of poisons is not explained in 89% of all episodes (Fig. 10). In the older episodes, explanations are even more rudimentary. By contrast, symptoms are shown significantly more often, with only 13% of all episodes having no presentation of symptoms (Fig. 10). Symptoms of poisoning tend to be presented more often over time. Over the years, there has been no significant improvement in terms of portraying the mechanism of action and symptoms (Fig. 10), although molecular pharmacology has made large advances in terms of explaining the mechanism of action of drugs and poisons alike. Consequently, *Tatort* remains largely descriptive in most episodes and misses its chance to entertain the medical education of a broad audience. In addition, explaining the underlying mechanism of poisoning can contribute to raising public awareness. The series also fails to take advantage of this opportunity. A broader understanding of a substance with its mechanism of action and its associated poisoning symptoms can promote early recognition of poisoning and thus make a major contribution to poisoning prevention.

### Fiction–reality comparison

In the figures published by the Poison Information Center Mainz, Germany, an increasing number of poisonings can be observed (GIZ-Mainz, Germany 2022). This tendency is also reflected by *Tatort*. Thus, *Tatort* depicts reality quite well in terms of the development of the total number of poisonings.

The annual comparison of real intentional poisonings shows that no significant changes in the substance categories have taken place. Over the years, drugs account for the largest share, followed by food, stimulants, smoking agents, and plants (GIZ-Mainz, Germany 2000, 2001, 2002, 2003, 2004, 2005, 2006, 2007, 2008). However, an increase in accidental drug poisonings can be observed (GIZ-Mainz, Germany 2000, 2001, 2002, 2003, 2004, 2005, 2006, 2007, 2008). When comparing the substance categories used in *Tatort* with reality, we find a high similarity. Drugs are the main causative category both in *Tatort* and in reality (Fig. 11). Cleaning and care products are the second most common cause of accidental poisoning in real life, which is underrepresented in *Tatort*. In *Tatort*, in analogy to reality, herbal poisonings play an important role. Food, stimulants, and intoxicants are overrepresented in *Tatort*.

The poisoning outcome in *Tatort* is different from reality: In *Tatort*, only 28% of the victims survive, whereas in real life, the survival rate amounts to 85% (Fig. 12). In addition, 55% of poisoning victims die in *Tatort*, contrasting with only 1% in real life. This distortion of reality is likely due to the striving for thrill and action in such a crime series.

The main route of administration in *Tatort* is oral, followed by inhalation and dermal application (Fig. 13). IV administration is significantly overrepresented in *Tatort*, accounting for 17% of the total, compared to 1% in reality. Ocular administration is not treated at all in *Tatort*.

The discrepancy between intentional and accidental poisoning in reality *versus Tatort* is probably due to the genre. Substance abuse is well represented in the series, whereas the high number of suicides is underrepresented (Fig. 14). Almost no accidental poisonings occur in *Tatort*, although this is the most frequent type of poisoning in real life. Again, this is certainly due to the crime genre. Consequently, there is an almost complete lack of information that accidental poisoning can affect anyone. Vulnerable groups in particular need to be made aware of this issue, e.g., elderly people with polypharmacy and several over-the-counter medications (Tesfamariam et al. 2019). In particular, accidental drug poisoning has increased (GIZ-Mainz, Germany 2000, 2001, 2002, 2003, 2004, 2005, 2006, 2007, 2008). In episode 342 (Schlotterbeck 1996), attention was drawn to poison exposure in the workplace. This is a good start into public awareness, but it is far from being sufficient and needs to be significantly improved.

### Gender analysis

There are substantially more men than women in *Tatort* overall, both as offenders and victims (Fig. 15). Among women, there are more victims than offenders. Here, the crime scene contradicts itself, since in episode 699 (Stelzer 2008) (min: 1:25:45–1:25–52), it is stated that poison murders are often carried out by women.

Figures from the GIZ, which refer to general poisonings and not to criminal cases, show that women are slightly more frequently affected by poisonings than men (Fig. 16) (GIZ-Mainz, Germany 2000, 2001, 2002, 2003, 2004, 2005, 2006, 2007, 2008, 2011).

Based on the figures for the true crime case series, it can be seen that there are more female offenders (Fig. 17) (Fuhrmeister 2005). This fits with the statement in the crime scene that poison murders are more likely to be committed by women (Stelzer 2008) (min: 1:25:45–1:25–52). There are also slightly more male victims (Fig. 17) (Fuhrmeister 2005). In conclusion, *Tatort* is substantially biased towards men, being more offensive and being more often a victim of poisoning than in reality.

### Lowest-rated episodes

Overall, significantly more episodes were rated high than low (Fig. 9). These high-rated episodes are predominantly found among the new episodes, whereas the lowest-rated episodes are mostly among the old episodes, indicating that professional medical advice to film directors improved over time.

There are particularly three episodes with significant deficits. Fictitious substances do not allow for a plausibility check and because of the few details provided, episode 364 (Panzer 1997) could be significantly improved. Furthermore, the trade names Valcordin and Tilur are mentioned with incorrect indication and with no further information (https://www.gelbe-liste.de/wirkstoffe/Doxylamin_21690, accessed 22 January 2022; https://medikamio.com/de-ch/medikamente/tilur-tilur-retard/pil, accessed 22 January 2022). Here, it is advisable to describe a fictitious toxin with its mechanism of action in detail and to present the symptoms. In addition, the two drugs would have to be explained. If an indication is mentioned, it should be the correct main indication.

In episode 1009 (Spirandelli 2017), the term “sleeping pill poisoning” is used, which provides a good basis for further substance explanations. However, these details are missing, the same as the main cause leading to death or respiratory paralysis (Hardman et al. 2001b, p. 418; Seifert 2019j, k, pp. 310ff, 328). Furthermore, the incorrect statement was made that taking this drug would probably not have killed a healthy person (Spirandelli 2017, min: 31:33–32:00). With such a statement, the educational mission of the *Tatort* is not only disregarded, but a fatal trivialization of taking unknown substances in unknown doses is made. Additionally, the substances are named in different ways: First, they are referred to as “sleeping pills, painkillers and ecstasy” (Spirandelli 2017, min: 31:33–32:00), then as “ecstasy and benzos” (Spirandelli 2017, min: 50:42–50:46) and finally as “painkillers and barbiturates” (Spirandelli 2017, min: 1:12:30–1:12:50). For a layperson, the erroneous impression could arise that benzodiazepines and barbiturates are very similar and reduce pain (Seifert 2019j, p. 310f). It would be much better to mention respiratory paralysis and to emphasize that any arbitrary substance combination is potentially dangerous. In addition, a clear and consistent substance naming and an explanation of the underlying mechanism of action should be given.

Poisoning with benzodiazepines and barbiturates is shown in episode 750 (Moore 2009), with mydriasis being the main symptom. However, miosis is more likely with these groups of drugs (Hufschmidt and Lücking 2009). During the episode, three other substances are mentioned that do not belong to these substance groups but do not have a proper name either. Thus, the layman could possibly assign these three substances to barbiturates or benzodiazepines. A clear separation of the drug groups is advisable here, and brief explanations of barbiturates, benzodiazepines, and the additional three substances should be provided. Furthermore, if only one symptom is mentioned, it is essential that this symptom be correct.

### Potential for imitation

On the one hand, we discuss the possibility of public awareness by delivering correct facts; on the other hand, the conflict of very plausible and detailed episodes with the resulting risk of imitation must be considered. Some episodes are so correct and detailed that they are a potential danger to victims and indirect guidance for perpetrators. In episode 437 (Heidelbach 2000a), instructions for suicide with KCN are provided. In a scenario involving palliative and terminally ill people, attention is drawn to euthanasia with KCN. Moreover, the necessary dose, route of administration, and procurement of KCN are described. Other studies confirm that self-injury addressed in the media has a negative impact on suicidality in the population and should therefore be treated sensitively (Hawton et al. 1999). This underpins the danger posed by such detailed episodes.

Reports on other crime stories, such as those by Agatha Christie, have shown how extensively and sometimes detailed poisonings can be portrayed (Platt and Platt 1994). In *Tatort*, poisonings are also sometimes shown in great detail. Therefore, on the one hand, both crime stories are partly rich in technically correct information, but on the other hand, the possible problem of imitation must be considered.

The challenge for the future production of further *Tatort* episodes is therefore to find a compromise between the correct representation of important social issues and a reduced potential for imitation. One possibility would be to slightly change the names of the active ingredients or to generally use fictitious names without being unrealistic.

### Limitations of our study

Although this is, to the best of our knowledge, the first scientific analysis of pharmacological topics in a TV series, we are fully aware that our study has limitations. First, we have no information on whether film directors had professional medical advice at hand. Second, in several cases, data analysis was exceedingly difficult because poisoning was presented only very unclearly and briefly, so we had to repeatedly view relevant episode sections at a slow speed. Third, because careful analysis of any given *Tatort* episode is extremely time-consuming, we had to limit our analysis to representative episodes covering almost 50 years. Fourth, we could not obtain access to all relevant *Tatort* episodes with poisonings. Thus, the study is not comprehensive. Fifth, the database of intoxications from the Giftinformationszentrum Mainz was incomplete and included a large gap (see the reference list). Sixth, some of the rating scales developed in this study are not totally objective but clearly have a subjective element. Finally, we analyzed only one crime series. It will be interesting to analyze which role poisonings play in crime series from other countries such as the USA, the UK, France, and Denmark. Despite these limitations, our analysis shows that poisonings play an important role in *Tatort* and how societal changes impacted the presentation of poisonings, clearly highlighting the cultural dimension of pharmacology and toxicology.

### Conclusions and future studies

In the crime series *Tatort*, some aspects of real poisoning exposures are reflected quite well, even though there are some deviations from reality, which are partly due to the crime genre. The presentation of poisonings improved over the years. Unfortunately, the mechanism of action of the poisoning substances is omitted in almost all episodes and should be incorporated into future episodes to promote public awareness. Film directors should not tacitly assume that the inclusion of mechanistic aspects of poisons is “too difficult to comprehend” for a general audience. In addition, more detailed information on the substances and a more comprehensive symptom presentation would be useful. Since poisoning affects all population groups, a crime series like *Tatort* with such a large audience can be a potential medium for public awareness and education. To date, this potential remains largely untapped. In the future, vulnerable groups should be particularly addressed, more accidental poisonings should be included, and prevention of poisoning should be discussed and implemented. Future *Tatort* episodes should also become more sensitive in terms of correctly depicting gender aspects of poisonings.

This initial study revealed that *Tatort* is a rich source for pharmacological analysis. We noted that in addition to the specific topic of poisonings covered in this study, drugs in general play an important role in this series, i.e., various actors take drugs to treat symptoms and diseases, are actively treated by physicians in episodes or comment on the efficacy, lack of efficacy, or adverse effects of drugs. One particularly rich source for analysis is the highly popular *Tatort Münster* in which Prof. Börne is a major figure. He is a professor of forensic medicine and comments (in a very arrogant manner) on multiple pharmacological topics in every episode. However, because he is “a scientific authority” despite his conduct, his statements may be taken as true. We will analyze to what extent therapeutic drug use is properly and correctly presented in *Tatort*.

## Supplementary Information

Below is the link to the electronic supplementary material.

Supplementary file1 (DOCX 291 KB)

Supplementary file2 (DOCX 28 KB)

## Data Availability

All source data for this study are supplied as supplementary data to this paper.

## References

[CR1] Agthe A (Director) (2002) Bienzle und der süße Tod [Film] SWR 505

[CR2] Aktories K, Förstermann U, Hofmann FB, Starke K, Forth W, Henschler D, Rummel W (2013). Muscarinrezeptor-Antagonisten. Allgemeine und spezielle Pharmakologie und Toxikologie.

[CR3] Aktories K, Förstermann U, Hofmann FB, Starke K, Forth W, Henschler D, Rummel W (2013). Psychopharmaka-Pharmakotherapie psychischer Erkrankungen. Allgemeine und spezielle Pharmakologie und Toxikologie.

[CR4] Aktories K, Förstermann U, Hofmann FB, Starke K, Forth W, Henschler D, Rummel W (2013). Pharmakotherapie der Herzinsuffizienz. Allgemeine und spezielle Pharmakologie und Toxikologie.

[CR5] Aktories K, Förstermann U, Hofmann FB, Starke K, Forth W, Henschler D, Rummel W (2013). Chemische Kanzerogenese. Allgemeine und spezielle Pharmakologie und Toxikologie.

[CR6] Aktories K, Förstermann U, Hofmann FB, Starke K, Forth W, Henschler D, Rummel W (2013). Metalle. Allgemeine und spezielle Pharmakologie und Toxikologie.

[CR7] Aktories K, Förstermann U, Hofmann FB, Starke K, Forth W, Henschler D, Rummel W (2013). Pestizide. Allgemeine und spezielle Pharmakologie und Toxikologie.

[CR8] Aktories K, Förstermann U, Hofmann FB, Starke K, Forth W, Henschler D, Rummel W (2013). Akute Probleme der Toxikologie. Allgemeine und spezielle Pharmakologie und Toxikologie.

[CR9] Aktories K, Förstermann U, Hofmann FB, Starke K, Forth W, Henschler D, Rummel W (2013). Giftpflanzen, Pflanzengifte. Allgemeine und spezielle Pharmakologie und Toxikologie.

[CR10] Auwärter V, Kneisel S, Hutter M, Thierauf A (2012). Synthetische Cannabinoide. Rechtsmedizin.

[CR11] Bannert W (Director) (1993) Ein Sommernachtstraum [Film] BR 278

[CR12] Baxmeyer F (Director) (2010) Schlafende Hunde [Film] Radio Bremen 765

[CR13] Baxmeyer F (Director) (2017) Dein Name sei Harbinger [Film] Studio.TV.Film für RBB/ARD Degeto 1038

[CR14] Bayerische Landesanstalt (ed) (2012) Beiträge zur Europäischen Lärche. LWF Wissen 69:79–81

[CR15] Becker W (Director) (1974) Acht Jahre später [Film] WDR 39

[CR16] Bernardi S (Director) (2017) Böser Boden [Film] NDR 1037

[CR17] Beubler E, Haltmayer H, Springer A (2006). Opiate aus heutiger Sicht. Opiatabhängigkeit: Interdisziplinäre Aspekte für die Praxis (German Edition).

[CR18] Blumenberg H C (Director) (1988) Salü Palu [Film] SR 201

[CR19] Bonhoff G (2013) Über Weckamine: Pervitin Und Benzedrin (Monographien aus dem Gesamtgebiete der Neurologie und Psychiatrie, (77) (1st ed.) [E-Book]. Springer, Berlin

[CR20] Booken D, Velten FW, Utikal J, Goerdt S, Bayerl C (2006). Allergische Kontaktdermatitis durch Kolophonium und Terpentin in Harzen unbehandelter Kiefernholzmöbel. Hautarzt.

[CR21] Bottinelli C, Cartiser N, Bévalot F, Fanton L, Guitton J (2020). Is insulin intoxication still the perfect crime? Analysis and interpretation of postmortem insulin: Review and perspectives in forensic toxicology. Crit Rev Toxicol.

[CR22] Brunker K, Mollentze N (2018). Rabies virus. Trends Microbiol.

[CR23] Bützer P (2016) “Alkohol” ethanol. ResearchGate. https://www.researchgate.net/publication/267376646_Alkohol_Ethanol. Accessed 23 June 2022

[CR24] Chahoud R (Director) (2019) Lakritz [Film] WDR 1107

[CR25] Chan TYK (2009). Aconite poisoning. Clin Toxicol.

[CR26] Corrêa BAAP, Sena VMAD, Matsushita RH, Citeli NK (2021). Report of envenomation in humans by handling a dyeing poison frog Dendrobates tinctorius (Schneider, 1799) (Anura: Dendrobatidae) in the Amazon, Brazil. Rev Soc Bras Med Trop.

[CR27] Dağ U (Director) (2018) Sonnenwende [Film] SWR 1058

[CR28] Datenblatt: Vergiftung-Antidiabetika (Insulin) (2021) San-Arena Erlangen. https://www.san-erlangen.de/VirtuelleSanArena-Erlangen-Html4/html/Topic28be3c98c6924d31ad89f6749113c24c.html. Accessed 23 June 2022

[CR29] Dekant W, Vamvakas S (2010). Rauschmittel oder psychotrope Substanzen. Toxikologie: Eine Einführung für Chemiker, Biologen und Pharmazeuten.

[CR30] Dekant W, Vamvakas S (2010). Pflanzengifte. Toxikologie: Eine Einführung für Chemiker, Biologen und Pharmazeuten.

[CR31] Dekant W, Vamvakas S (2010). Bakterielle Toxine in Nahrungsmitteln. Toxikologie: Eine Einführung für Chemiker, Biologen und Pharmazeuten.

[CR32] DIE WELT (2012) Münster: Kind mit Terpentin vergiftet – Drei Jahre Haft. https://www.welt.de/regionales/duesseldorf/article112107299/Kind-mit-Terpentin-vergiftet-Drei-Jahre-Haft.html. Accessed 23 June 2022

[CR33] Editorial Gelbe Liste Pharmindex (2016) Acemetacin. Gelbe Liste. Pharmindex. https://www.gelbe-liste.de/wirkstoffe/Acemetacin_3180. Accessed 23 June 2022

[CR34] Editorial Gelbe Liste Pharmindex (2022) Valocordin®-Doxylamin, 25 mg/ml Tropfen zum Einnehmen, Lösung. Gelbe Liste. Pharmindex. https://www.gelbe-liste.de/produkte/Valocordin-Doxylamin-25-mg-ml-Tropfen-zum-Einnehmen-Loesung_508539. Accessed 15 June 2022

[CR35] Emmerich K (Director) (2003) Wenn Frauen Austern essen [Film] BR 542

[CR36] Esposti MD, Ngo A, Myers MA (1996). Inhibition of mitochondrial complex i may Account for IDDM induced by intoxication with the rodenticide vacor. Diabetes.

[CR37] Fischer B, Patra J, Cruz MF, Gittins J, Rehm J (2008). Comparing heroin users and prescription opioid users in a Canadian multi-site population of illicit opioid users. Drug Alcohol Rev.

[CR38] Fischer M (Director) (2000) Trittbrettfahrer [Film] Colonia Media 447

[CR39] Fischer T C (Director) (2014) Der Fall Reinhardt [Film] WDR 905

[CR40] FNR-KO-Tropfen-Aerzteinformation.pdf (w. d.) https://www.soforthilfe-nach-vergewaltigung.de/fileadmin/redaktion/pdf/FNR-KO-Tropfen-Aerzteinformation.pdf. Accessed 29 June 2022

[CR41] Freundner T (Director) (1998) Gefallene Engel [Film] BR 397

[CR42] Fuhrmeister A C (2005) Vergiftungen Panoramawechsel der letzten Jahrzehnte. Ergebnisse einer Literaturstudie. https://core.ac.uk/display/304640338?utm_source=pdf&utm_medium=banner&utm_campaign=pdf-decoration-v1. Accessed 28 July 2022

[CR43] Garde C (Director) (2006) Schattenspiele [Film] NDR 637

[CR44] Gies H (Director) (1977) Das Mädchen von gegenüber [Film] WDR 82

[CR45] GIZ-Mainz (2000) GIZ-Mainz-Jahresbericht-2000 https://www.unimedizin-mainz.de/typo3temp/secure_downloads/24969/0/1b172257662e2e60ac1f72939c752284b4d17bf4/GIZ-Mainz-Jahresbericht-2000.pdf. Accessed 29 June 2022

[CR46] GIZ-Mainz (2001) GIZ-Mainz-Jahresbericht-2001 https://www.unimedizin-mainz.de/typo3temp/secure_downloads/24969/0/1b172257662e2e60ac1f72939c752284b4d17bf4/GIZ-Mainz-Jahresbericht-2001.pdf. Accessed 29 June 2022

[CR47] GIZ-Mainz (2002) GIZ-Mainz-Jahreskurzbericht-2002 https://www.unimedizin-mainz.de/typo3temp/secure_downloads/24969/0/1b172257662e2e60ac1f72939c752284b4d17bf4/GIZ-Mainz-Jahresbericht-2002.pdf. Accessed 29 June 2022

[CR48] GIZ-Mainz (2003) GIZ-Mainz-Jahreskurzbericht-2003 https://www.unimedizin-mainz.de/typo3temp/secure_downloads/24969/0/1b172257662e2e60ac1f72939c752284b4d17bf4/GIZ-Mainz-Jahresbericht-2003.pdf. Accessed 29 June 2022

[CR49] GIZ-Mainz (2004*)* GIZ-Mainz-Jahreskurzbericht-2004 https://www.unimedizin-mainz.de/typo3temp/secure_downloads/24969/0/1b172257662e2e60ac1f72939c752284b4d17bf4/GIZ-Mainz-Jahresbericht-2004.pdf. Accessed 29 June 2022

[CR50] GIZ-Mainz (2005) GIZ-Mainz-Jahresbericht-2005 https://www.unimedizin-mainz.de/typo3temp/secure_downloads/24969/0/1b172257662e2e60ac1f72939c752284b4d17bf4/GIZ-Mainz-Jahresbericht-2005.pdf. Accessed 29 June 2022

[CR51] GIZ-Mainz (2006) GIZ-Mainz-Jahresbericht-2006 https://www.unimedizin-mainz.de/typo3temp/secure_downloads/24969/0/1b172257662e2e60ac1f72939c752284b4d17bf4/GIZ-Mainz-Jahresbericht-2006.pdf. Accessed 29 June 2022

[CR52] GIZ-Mainz (2007) GIZ-Mainz-Jahresbericht-2007 https://www.unimedizin-mainz.de/typo3temp/secure_downloads/24969/0/1b172257662e2e60ac1f72939c752284b4d17bf4/GIZ-Mainz-Jahresbericht-2007.pdf. Accessed 29 June 2022

[CR53] GIZ-Mainz (2008) GIZ-Mainz-Jahresbericht-2008 https://www.unimedizin-mainz.de/typo3temp/secure_downloads/24969/0/1b172257662e2e60ac1f72939c752284b4d17bf4/GIZ-Mainz-Jahresbericht-2008.pdf. Accessed 29 June 2022

[CR54] GIZ-Mainz (2011) GIZ_Mainz_Jahresbericht_2011 https://www.unimedizin-mainz.de/typo3temp/secure_downloads/24969/0/1b172257662e2e60ac1f72939c752284b4d17bf4/GIZ_Mainz_Jahresbericht_2011.pdf. Accessed 29 June 2022

[CR55] GIZ-Mainz (2022) Jahreskurzbericht 2021

[CR56] Gmbh B (w. d.) TilurÂ®/Tilur retardÂ® - Gebrauchsinformation. Medikamio - Dein persönlicher Arzneimittel-Assistent. https://medikamio.com/de-ch/medikamente/tilur-tilur-retard/pil. Accessed 15 June 2022

[CR57] Gmbh B (2022) Valocordin-Diazepam - Gebrauchsinformation. Medikamio - Dein persönlicher Arzneimittel-Assistent. https://medikamio.com/de-de/medikamente/valocordin-diazepam/pil. Accessed 27 March 2022

[CR58] Gräwert G (Director) (1977) Wer andern eine Grube gräbt [Film] SR 76

[CR59] Gusovsky F, Rossignol DP, McNeal ET, Daly JW (1988). Pumiliotoxin B binds to a site on the voltage-dependent sodium channel that is allosterically coupled to other binding sites. Proc Natl Acad Sci.

[CR60] Haffter P (Director) (1995) Ein ehrenwertes Haus [Film] MDR 302

[CR61] Hardman JG, Limbird LE, Goodman Gilman A (2001). Catecholamines, sympathomimetic drugs, and adrenergic receptor antagonists. Goodman & Gilman’s The Pharmacologic Basis of Therapeutics.

[CR62] Hardman JG, Limbird LE, Goodman Gilman A (2001). Hypnotics and Sedatives. Goodman & Gilman’s The Pharmacologic Basis of Therapeutics.

[CR63] Hardman JG, Limbird LE, Goodman Gilman A (2001). Ethanol. Goodman & Gilman’s The Pharmacologic Basis of Therapeutics.

[CR64] Hardman JG, Limbird LE, Goodman Gilman A (2001). Drug addiction and drug abuse. Goodman & Gilman’s The Pharmacologic Basis of Therapeutics.

[CR65] Hardman JG, Limbird LE, Goodman Gilman A (2001). Nonmetallic enviromental toxicants: Air pollutants, solvents and vapors, and pesticides. Goodman & Gilman’s The Pharmacologic Basis of Therapeutics.

[CR66] Hawton K, Simkin S, Deeks JJ, O’Connor S, Keen A, Altman DG, Philo G, Bulstrode C (1999). Effects of a drug overdose in a television drama on presentations to hospital for self poisoning: Time series and questionnaire study. BMJ.

[CR67] Heidelbach K (Director) (2000a) Bittere Mandeln [Film] WDR 437

[CR68] Heidelbach K (Director) (2000b) Quartett in Leipzig [Film] WDR, MDR 458

[CR69] Henning R (Director) (2019) One way ticket [Film] BR 1114

[CR70] Hufschmidt A, Lücking CH (2009) Capture 2.18. In: Neurologie compact: Für Klinik und Praxis, 5th edn. Thieme, Stuttgart. 10.1055/b-0034-4372

[CR71] Imboden M (Director) (2017) Am Ende geht man nackt [Film]

[CR72] Ionescu-Pioggia M, Bird M, Orzack MH, Benes F, Beake B, Cole JO (1988). Methaqualone. Int Clin Psychopharmacol.

[CR73] Jauch T (Director) (2003) Sonne und Sturm [Film] NDR 545

[CR74] Jauch T (Director) (2014) Mord ist die beste Medizin [Film] WDR 917

[CR75] Jessen L (Director) (2016) Feierstunde [Film] WDR 994

[CR76] Kahle C (2020) Doxylamin. Gelbe Liste. Pharmindex. https://www.gelbe-liste.de/wirkstoffe/Doxylamin_21690. Accessed 23 June 2022

[CR77] Kleinert A (Director) (2019) Die ewige Welle [Film] BR 1096

[CR78] Koch P (Director) (2018) Im toten Winkel [Film] Radio Bremen 1051

[CR79] Kren M (Director) (2015) Die letzte Wiesn [Film] BR 956

[CR80] Lehmann E (2021) Tatort Dresden: Gibt es tödliche Nanobots wirklich? Prisma. https://www.prisma.de/news/tv/Tatort-Dresden-Gibt-es-toedliche-Nanobots-wirklich,31501837. Accessed 23 June 2022

[CR81] Loose L (Director) (2022) Das Herz der Schlange [Film] SR 1187

[CR82] Marczynski B, Rozynek P, Huber H, Baur X (1999). Gentoxizität von Asbestfasern - eine Literaturauswertung. Pneumologie.

[CR83] Marka S (Director) (2017) Der scheidende Schupo [Film] MDR 1010

[CR84] Marka S (Director) (2019) Ein Tag wie jeder andere [Film] BR 1085

[CR85] Marka S (Director) (2021) Unsichtbar [Film] MDR 1174

[CR86] Meletzky F (Director) (2022) Des Teufels langer Atem [Film] WDR 1186

[CR87] Moore E (Director) (2009) Altlasten [Film] SWR 750

[CR88] Neureuther E (Director) (1978) Der Mann auf dem Hochsitz [Film] SWF 84

[CR89] Panzer W (Director) (1997) Tödlicher Galopp [Film] MDR 364

[CR90] Pfeiffer M (Director) (2008) Brandmal [Film] WDR 708

[CR91] Platt OS, Platt R (1994). The Poisonous Pen of Agatha Christie. N Engl J Med.

[CR92] Ranisch A (Director) (2017) Babbeldasch [Film] SWR 1012

[CR93] Renner UD, Oertel R, Kirch W (2005). Pharmacokinetics and Pharmacodynamics in Clinical Use of Scopolamine. Ther Drug Monit.

[CR94] Schlotterbeck D (Director) (1996) Bienzle und der Traum vom Glück [Film] SDR 342

[CR95] Seidel D, Solbach T, Fehse R, Donke L, Elliehausen H J (2007) Asbestbedingte Erkrankungen (BK-Nrn. 4103, 4104 und 4105). Gesundheitsberichterstattung Bundes 38: 24–26

[CR96] Seifert R (2019). Pathophysiology and therapy of type 1 reaction and pseudoallergic reactions. Basic knowledge of pharmacology.

[CR97] Seifert R (2019). Pharmacological modulation of the cholinergic synapse. Basic knowledge of pharmacology.

[CR98] Seifert R (2019). (Patho)physiological background and histamine receptors. Basic knowledge of pharmacology.

[CR99] Seifert R (2019). Pain pathophysiology. Basic knowledge of pharmacology.

[CR100] Seifert R (2019). Pharmacological interventions for pain therapy. basic knowledge of pharmacology.

[CR101] Seifert R (2019). MOR Agonists. Basic knowledge of pharmacology.

[CR102] Seifert R (2019). Important clinical studies on CHF pharmacotherapy. Basic knowledge of pharmacology.

[CR103] Seifert R (2019). Pathophysiology of diabetes mellitus (DM) and Pharmacotherapeutic concepts. Basic knowledge of pharmacology.

[CR104] Seifert R (2019). Insulins. Basic knowledge of pharmacology.

[CR105] Seifert R (2019). Allosteric GABA_A_R modulators. Basic knowledge of pharmacology.

[CR106] Seifert R (2019k) Important injection anesthetics. In: Basic knowledge of pharmacology, 1st edn. Springer International Publishing, Basel, p 328

[CR107] Seifert R (2019l) Pathophysiology of depression and pharmacotherapeutic concepts. In: Basic knowledge of pharmacology, 1st edn. Springer International Publishing, Basel, p 332

[CR108] Seifert R (2019m) Pathophysiology of dementias and pharmacotherapeutic concepts. In: Basic knowledge of pharmacology, 1st edn. Springer International Publishing, Basel, p 358

[CR109] Senti G, Ballmer-Weber B K, Wüthrich B (2000) Nuts, seeds and grains from an allergist’s point of view. Swiss Med Wkly 130:1801–1802. https://www.researchgate.net/profile/Brunello-Wuethrich/publication/12199084_Nuts_seeds_and_grains_from_an_allergist's_point_of_view/links/02e7e5292660e3f8c2000000/Nuts-seeds-and-grains-from-an-allergists-point-of-view.pdf. Accessed 29 June 2022 11130145

[CR110] Series editing “RKI-Ratgeber” (2020) RKI - RKI-Ratgeber - Tollwut. Robert Koch Institut. https://www.rki.de/DE/Content/Infekt/EpidBull/Merkblaetter/Ratgeber_Tollwut.html. Accessed 23 June 2022

[CR111] Spirandelli Z (Director) (2017) Söhne und Väter [Film] ProSaar Medienproduktion 1009

[CR112] Staudte W (Director) (1977) Spätlese [Film] WDR 75

[CR113] Stein MS (2003) Stellungnahme zur Nicht Geringen Menge von γ-Hydroxybuttersäure. T + K, 70(2):87–92

[CR114] Stelzer M (Director) (2008) Krumme Hunde [Film] WDR 699

[CR115] Taniguchi H, Yamashiro Y, Chung MY, Hara Y, Ishihara K, Ejiri K, Baba S (1989). Vacor inhibits insulin release from islets in vitro. J Endocrinol Invest.

[CR116] Teesson M, Ross J, Darke S, Lynskey M, Ali R, Ritter A, Cooke R (2006). One year outcomes for heroin dependence: findings from the Australian Treatment Outcome Study (ATOS). Drug Alcohol Depend.

[CR117] Tesfamariam S, Anand IS, Kaleab G, Berhane S, Woldai B, Habte E, Russom M (2019). Self-medication with over the counter drugs, prevalence of risky practice and its associated factors in pharmacy outlets of Asmara. Eritrea BMC Public Health.

[CR118] Thebault L, Lesne J, Boutin JP (1995). Cyanobacteria, their toxins and health risks. Med Trop.

[CR119] Thiel C (1991). Lebensmittelallergien und-intoleranzreaktionen. Z Ernährungswiss.

[CR120] Trendelenburg G, Ströhle A (2005). γ-Hydroxybuttersäure — Neurotransmitter, Medikament und Droge. Nervenarzt.

[CR121] van Riel AJHP, Schets FM, Meulenbelt J (2007). The effects of blue algae on health. Ned Tijdschr Geneeskd.

[CR122] Verba H (2007). KO-Tropfen und “date rape” – Verabreichung von Drogen zur Begehung von Sexualstraftaten. Z Psychotraumatol Psychother Psychol Med.

[CR123] Vogel P (Director) (1997) Der Tod spielt mit [Film] MDR 366

[CR124] Walliczek-Dworschak U (2019) Modafinil. Gelbe Liste. Pharmindex. https://www.gelbe-liste.de/wirkstoffe/Modafinil_27434. Accessed 23 June 2022

[CR125] Wikipedia authors (2022) K.-o.-Tropfen. Wikipedia. https://de.wikipedia.org/w/index.php?title=K.-o.-Tropfen&oldid=219449141. Accessed 23 June 2022

[CR126] Wirth F P (Director) (1975) Wodka Bitter-Lemon [Film] WDR 50

[CR127] Zahavi D (Director) (2016) Der König der Gosse [Film] MDR 995

[CR128] Zahavi D (Director) (2018) Tollwut [Film] WDR 1046

[CR129] Ziegler C, Rutscher R, Rühl R (2014). Unterschätzte Abgasbelastungen. Sicher ist sicher Arbeitsschutz aktuell.

[CR130] Zuardi AW (2006). History of cannabis as a medicine: a review. Rev Bras Psiquiatr.

