# Supplementary material for: Poisoning cases in the German crime series Tatort (crime scene) from 1974 to 2022
Source: Naunyn Schmiedebergs Arch Pharmacol. 2022 Aug 16;395(11):1419–40. doi: 10.1007/s00210-022-02281-9 (PMC9568490; doi:10.1007/s00210-022-02281-9)
Supplement: Supplementary file 1 — Supplementary file1 (DOCX 291 KB) [file 210_2022_2281_MOESM1_ESM.docx]

**Poisoning cases in the German crime series *Tatort*
(crime scene) from 1974 to 2022**

**Rachel Ellerbeck⋅ and Roland Seifert**

**Supplementary Table 1. Detailed analysis of the *Tatort* episodes**

**Table of Contents**

The Heart of the Snake (1187) …………………………………………………………………………. 1

The Devil's Long Breath (1186) ………………………………………………………………………… 3

Invisible (1174) ……………………………………………………………………………………………….. 6

One Way Ticket (1114) ……………………………………………………………………………………. 9

Licorice (1107) ………………………………………………………………………………………………… 11

The Eternal Wave (1096) ………………………………………………………………………………… 14

A Day Like Any Other (1085) …………………………………………………………………………… 16

Solstice (1058) ………………………………………………………………………………………………… 18

In the Blind Spot (1051) ………………………………………………………………………………….. 20

Rabies (1046 …………………………………………………………………………………………………… 22

Thy name be Harbinger (1038) ……………………………………………………………………….. 26

Evil Ground (1037) ………………………………………………………………………………………….. 27

In the End You Go Naked (1018) ………………………………………………………………………. 32

Babbeldash (1012) ………………………………………………………………………………………….. 33

The outgoing Schupo (1010) …………………………………………………………………………… 35

Sons and fathers (1009) ………………………………………………………………………………….. 37

The King of the Gutter (995) ……………………………………………………………………………. 39

Celebration (994) ……………………………………………………………………………………………. 41

The last Oktoberfest (956) ……………………………………………………………………………… 45

Murder is the Best Medicine (917) ………………………………………………………………….. 47

The Legal Case Reinhardt (905) ………………………………………………………………………. 52

Sleeping Dogs (765) ……………………………………………………………………………………….. 54

Legacy sites (750) …………………………………………………………………………………………… 56

Burn mark (708) …………………………………………………………………………………………….. 58

Crooked dogs (699) ………………………………………………………………………………………… 59

Shadow Games (637) ……………………………………………………………………………………… 60

Sun and Storm (545) ……………………………………………………………………………….………. 63

When Women Eat Oysters (542) …………………………………………………………………….. 65

Bienzle and the Sweet Death (505) …………………………………………………………………. 66

Quartet in Leipzig (458) ………………………………………………………………………………….. 68

Free Rider (447) ………………………………………………………………………………………………. 70

Bitter Almonds (437) ……………………………………………………………………………………….. 71

Fallen Angels (397) ………………………………………………………………………………………….. 74

Death plays along (366) ……………………………………………………………………….............. 76

Deadly Gallop (364) …………………………………………………………………………………………. 78

Bienzle and the Dream of Happiness (342) ………………………………………………………. 80

An honorable house (302) ……………………………………………………………………………….. 82

A Midsummer Night's Dream (278) ………………………………………………………………….. 84

Salut Palu (201) ………………………………………………………………………………………………. 85

The Man on the High Seat (84) ……………………………………………………………………….. 87

The Girl from the House on the Opposite Side of the Street (82) ……………………… 89

Harm set, harm get (76) ……………………………………………………………………………….…. 90

Late Vintage (75) …………………………………………………………………………………………….. 92

Vodka Bitter Lemon (50) ………………………………………………………………………………….. 93

Eight Years Later (39) …………………………………………………………………………............... 95

| *Tatort* episode | First broadcast | Location | Time period | Action | Active ingredient/group of active ingredients | Mechanism of action/symptoms | Literature | Plausibility/Detailedness | Time comparison |
| --- | --- | --- | --- | --- | --- | --- | --- | --- | --- |
| *The Heart of the Snake (1187) (3)* | 23.01.2022 | Saarbrücken | min: 08:35-08:50 | Inspector Adam Schürk sits down on an armchair at his father's house and injures himself with a pointed object | **Frog’s toxin:** Name of frog and name of toxin unknown; red color indicates affiliation with aposematic animals (color indicates toxicity) (1); probably group of Dendrobatidea (1); Dendrobates tinctorius (blue poison dart frog) possible example (1); lives in "Central America, French Guayana, Surinam and in the north of Brazil" (1); the toxins consist of alkaloid compounds (1); main alkaloid = pumiliotoxin (PTX) -> highly toxic in high concentrations (1) | **Pumiliotoxin:** exact physiology unknown (1); affects calcium channels (1) and increases sodium influx in cerebral synaptoneurosomes (2); cardiotonic, myotonic effects (2); symptoms: "movement disorders, clonic spasms, paralysis" (1); potentially lethal (1); respiratory and cardiac failure as possible cause of death (1) | **(1)** Correa et al. (2021); **(2)** Gusovsky et al. (1988); **(3)** Loose (2022) | **Symptoms:** shortness of breath, paralysis, heart problems - plausible; **mechanism of action:** paralysis only on striated muscle - implausible; **substance explanation:** nerve toxin, myotonolytic - plausible; frog name is missing; **latency:** no; **dose:** no; **cause of death:** no; **route of administration:** needle - plausible; toxin in vomit only when ingested orally - plausible; use in anesthesia (no active ingredient name is given) not described - implausible; **P:** 2; **D:** 3 | no |
|  |  |  | min: 11:10-11:53 | Adam breathes heavily and sweats |  |  |  |  |  |
|  |  |  | min: 19:51-20:21 | An African Cape cobra is taken from Adam's father's terrarium; it is a small, very poisonous cobra; the frog in the terrarium is also poisonous |  |  |  |  |  |
|  |  |  | min: 29:17-31:25 | Adam no longer has shortness of breath or other symptoms; he still remembers what happened |  |  |  |  |  |
|  |  |  | min: 37:55 | Adam reports that something stabbed him in the back when he sat down |  |  |  |  |  |
|  |  |  | min: 47:07-50:40 | Adam is physically examined upon admission to custody -> no pathological findings; Adam remembers the night before: his father asks him: "Are you having trouble breathing, son? Tell me something. Oh ……. this means it already has an effect. Your eye muscles should still be working somehow. It then slowly moves up. You are the snake, I am the frog. (...) The frog produces a poison, which only paralyzes the striated muscles. Your organs continue to function. Your heart muscle is also striated, but has an excitation system, the sinus node. Pretty unpleasant effect. I tested the dose on myself. It almost killed me twice. You'll be completely paralyzed for 6-8 hours. You might even die."; Adam is in severe respiratory distress; his father flushes a needle down the sink. |  |  |  |  |  |
|  |  |  | min: 56:35-56:41 | Inspector Leo Hölzer reports that Adam suspects he was poisoned by the frog’s toxin |  |  |  |  |  |
|  |  |  | min: 1:01:40-1:02:23 | The forensic pathologist found no traces of poison in the vomit from the crime scene; "It would only have made sense if it had been ingested orally - and not injected or inhaled"; the veterinarian knows nothing about the frog; "The frog produces a strong neurotoxin. It is a substance from the group of myotonolytics. It causes reversible paralysis of the skeletal muscles. The substance is used in anesthesia. Animal origin was not known until now." |  |  |  |  |  |

| *Tatort* episode | First broadcast | Location | Time period | Action | Active ingredient/group of active ingredients | Mechanism of action/symptoms | Literature | Plausibility/Detailedness | Time comparison |
| --- | --- | --- | --- | --- | --- | --- | --- | --- | --- |
| *The Devil's Long Breath (1186) (9)* | 16.01.2022 | Münster | min: 00:40-01:55 | Inspector Thiel wakes up and doesn't know how he got into the room; the night before he called Boerne, was confused and cried | **Scopolamine:** natural alkaloid of belladonna (Atropa belladonna), henbane (Hyoscyamus niger) and datura (Datura stramonium) (1); member of the nightshade family (1). The plant is found among others in South America (2); partly used in medicine (3); also abused as a drug called "Devil's Breath" or "Burundanga" (2); ingested via food and drinks, or blown into the face (7); abused by governments in the 1950s (7); difficult to detect (2) | **Scopolamine:** Nonselective muscarinic antagonism (3); has "peripheral antimuscarinic properties as well as central sedative, antiemetic, and amnestic effects" (3); inability to retain new information and stay attentive (4); causes visual and auditory hallucinations, euphoria, fatigue, disorientation, and motor incoordination (4); typical symptoms of anticholinergic syndrome "ataxia, disorientation, short-term memory loss, confusion, hallucinations, psychosis, excited delirium, seizures, coma, respiratory failure, or cardiovascular collapse" (5); "submissive and obedient behavior" due to "reduction of declarative memory" (2); criminal abuse to have victims withdraw money, for example (2) | **(1)** Aktories et al. (2013), p. 132; **(2)** Reichert et al. (2017); **(3)** [Renner](https://journals.lww.com/drug-monitoring%20/Abstract/2005/10000/Pharmacokinetics_and_Pharmacodynamics_in_Clinical.19.aspx) et al. (2005); **(4)** Safer, Allen (1971); **(5)** Adnane Berdai et al. (2012); **(6)** Antwerpes et al. (2019); **(7)** Nestler (2021); **(8)** Hardman et al. (2001), p. 429; **(9)** Meletzky (2022) | **Symptoms:** Amnesia, hallucinations, lack of will, accept orders - plausible; headache, nausea, vomiting, alcohol consumption - plausible (8); **mechanism of action:** no; **substance explanation:** naming of substance: Devil's breath, Burundanga - plausible; origin: Colombia- plausible (6); molecular formula- plausible (6); **latency:** no; **dose:** no; **cause of death:** no; **route of administration:** inhaled - plausible; previously used by governments - plausible; difficult to detect - plausible; many details (names, Colombia, lack of will, zombie, carrying out orders, story with valuables handed out, powder blown in face, amnesia) coincide with the article "Devil's Breath – ausgeliefert, willenlos, ein Zombie" from Hanf Magazin, 13.06.2017 (7); **P:** 1; **D:** 1 | no |
|  |  |  | min: 04:42 | Inspector Thiel asks for headache pills |  |  |  |  |  |
|  |  |  | min: 07:59-08:23 | Inspector Thiel vomits, does not know where his car and the key are and complains of a headache. |  |  |  |  |  |
|  |  |  | min: 16:14-17:24 | Inspector Thiel has repeated flashes of memory; he thinks he was very drunk; Boerne notices that he has no memories at all |  |  |  |  |  |
|  |  |  | min: 18:30-19:04 | The assumption that something was poured into his beer comes up |  |  |  |  |  |
|  |  |  | min: 26:51-27:16 | Inspector Thiel says: "As if I had the memories of a total stranger in my head"; “colorful images always come into my head” |  |  |  |  |  |
|  |  |  | min: 29:15-29:36 | Presumption that he was administered knockout drops; however, this presumption is immediately disproved as he was still able to drive home and perform other yet unknown acts |  |  |  |  |  |
|  |  |  | min: 41:47-42:18 | Thiel says again that he has no memories of what happened; it is "all erased" |  |  |  |  |  |
|  |  |  | min: 43:38-43:58 | The result of Thiel's blood test: "A blood alcohol level of 0.07 %. Apart from that, no abnormalities. No benzodiazepines, no ketamine, GHB inapparent." |  |  |  |  |  |
|  |  |  | min: 1:09:44-1:11:09 | In a video, a man confirms an inheritance and is conspicuously acquiescent; Thiel appears very similar in the monitoring video; "Frank is never that submissive usually", reports Thiel's father; he also suspects it's scopolamine; also called "the devil’s breath" |  |  |  |  |  |
|  |  |  | min: 1:15:42-1:17:55 | Thiel's father reports:" Burundanga is mainly found in Colombia. The active ingredient is scopolamine and is extracted from datura, for example. Chemical formula is C17H21NO4. Structural formula looks something like this. Dosing is very difficult. A little too much of it and you drop dead."; "And at the right dose, it makes the user lose his own will. There are cases where people even have helped clean out their own apartments or have withdrawn all their money from their bank accounts and given it to the perpetrators and the next day they don't remember it. Burundanga makes a zombie out of you"; In blood it is only detectable within the first 6-8 hours; Dr. Kühn lived in Colombia and when she was back in Germany, a sick man had bequeathed his inheritance to her; Intake: blown or inhaled; Thiel remembers that the victim blew something in his face, whereupon he did everything he was told to do. |  |  |  |  |  |
|  |  |  | min: 1:23:31-1:24:00 | Boerne says: "Scopolamine, the good old devil's stuff. But that's not quite correct. It had been used by the CIA as a truth drug, until they realized that people on scopolamine said anything but the truth. In exchange, they admitted anything that is asked of them. Completely will-less. Many of them committed suicide because of the severe hallucinations as side effects." |  |  |  |  |  |

| *Tatort* episode | First broadcast | Location | Time period | Action | Active ingredient/group of active ingredients | Mechanism of action/symptoms | Literature | Plausibility/Detailedness | Time comparison |
| --- | --- | --- | --- | --- | --- | --- | --- | --- | --- |
| *Invisible (1174) (2)* | 17.10.2021 | Dresden | min: 01:06-01:44 | Hand pain, dropping to her knees -> inspector Karin Gorniak | **NanoBots:** as depicted here, fictitious (1); production of artificial proteins under development (1) | No mechanism because fictitious | **(1)** Lehmann (2021); **(2)** Marka (2021) | **Symptoms:** pain, inability to move, hot, red skin - fictitious; **Mechanism of action:** Adherence to nonspecific nerve and pain fibers of autonomic nervous system - fictitious; **Substance explanation:** altered molecules, charged with amino acids - fictitious; **Latency:** no; **Dose:** no; **Cause of death:** sudden cardiac death - fictitious; **Route of administration:** absorbed via the skin - fictitious; **P:** cannot be rated because fictitious; **D:** 2 | no |
|  |  |  | min: 04:09-04.19 | Problem with the right hand (not specified at the moment) -> inspector |  |  |  |  |  |
|  |  |  | min: 06.15-07:25 | Skin burning on the arms, aggravation with movement -> both women, Anna Schneider is more affected. |  |  |  |  |  |
|  |  |  | min: 08:29-08:43 | Collapse and death -> Anna Schneider |  |  |  |  |  |
|  |  |  | min: 09.38-09.45 | Problem with the right hand (not specified at the moment) -> inspector |  |  |  |  |  |
|  |  |  | min: 11:01-11:27 | Description of Anna Schneider's symptoms: "pain from the slightest touch"; hot, red hands, inability to move |  |  |  |  |  |
|  |  |  | min: 12:29-12:50 | No forensic evidence of substances that could explain the symptoms; various neurologists have no explanation (diagnosis: hypersensitivity to pain) -> Anna Schneider |  |  |  |  |  |
|  |  |  | min: 16:28-17:17 | MRI brain; blood withdrawal; x-ray right forearm, right hand, CT, nerve response NAD; nothing that explains pain -> inspector. |  |  |  |  |  |
|  |  |  | min: 18:30-19:00 | Inspector describes the symptoms: 'Fell down because of pain in leg, then in hand' |  |  |  |  |  |
|  |  |  | min: 19:43-21:39 | Preliminary autopsy report: "sudden cardiac death" of unclear etiology; exclusion of drugs and 24 conventional toxins; ECG by smart watch: first fibrillation, then slowing down of heart rate and cardiac arrest; no pre-existing disease; redness on hand, from back to buttocks (no allergy). |  |  |  |  |  |
|  |  |  | min: 22:40-23:00 | Short-term pain -> inspector |  |  |  |  |  |
|  |  |  | min: 32:00-32:07 | Pain right hand -> inspector |  |  |  |  |  |
|  |  |  | min: 46:45 | Collapse -> inspector |  |  |  |  |  |
|  |  |  | min: 50:03-50:33 | Administration of poison to Anna Schneider's ex boyfriend Nils Klotsche |  |  |  |  |  |
|  |  |  | min: 51:00-51:43 | Presumably pain on touching; oral intake of a substance - thereafter improvement -> laboratory employee Martha Marczynski |  |  |  |  |  |
|  |  |  | min: 52:16 | Collapse -> Anna Schneider's ex boyfriend |  |  |  |  |  |
|  |  |  | min: 53:02-53:36 | Description of the substance: altered molecules that are charged with amino acids so that they dock to the non-specific nerve and pain fibers of the autonomic nervous system; cause of sensory and autonomic neuropathy and dose-dependent cardiac arrest. |  |  |  |  |  |
|  |  |  | min: 55:55 | Description of the successful development of a weapon which, after diffusion into the blood, is no longer detectable there. |  |  |  |  |  |
|  |  |  | min: 58:00-58:30 | Pain subsides -> inspector; Professor has developed a painkiller against NanoBots |  |  |  |  |  |
|  |  |  | min: 1:05:52- 1:06:12 | Administration of an injection to the inspector by Anna Schneider's ex-boyfriend; then unconsciousness |  |  |  |  |  |
|  |  |  | min: 1:08:30-1:10:33 | Description of the effect of the molecules: increase of pain sensation to infinity, cardiac arrest on exertion; pain in the hand; |  |  |  |  |  |
|  |  |  | min: 1:26:07-1:26:35 | Infusion and recovery -> inspector |  |  |  |  |  |

| *Tatort* episode | First broadcast | Location | Time period | Action | Active ingredient/group of active ingredients | Mechanism of action/symptoms | Literature | Plausibility/Detailedness | Time comparison |
| --- | --- | --- | --- | --- | --- | --- | --- | --- | --- |
| *One Way Ticket (1114) (3)* | 26.12.2019 | Munich | min: 01:15 | Traffic accident for still unknown reason -> victim Timo Harbig | **Phorbol:** toxin from spurge plants (Euphorbiaceae) (1); milk sap contains fatty acid esters of the diterpene alcohol phorbol (1); toxic to humans and animals (2); varies in toxicity depending on the plant (2); native country: Angola (2) | **Phorbol:** skin irritation (local), toxic, and cocarcinogenic (1); symptoms: fatal necrotizing/hemorrhagic gastroenteritis, resorptive toxicity: renal injury (1); skin contact: blistering and necrosis (1); eye contact: keratitis (1) | **(1)** Aktories et al. (2013), p. 1080; **(2)** Lochstampfer (2018); **(3)** Henning (2019) | **Symptoms:** bloody vomiting, kidney failure - plausible; **mechanism of action:** no; **substance explanation:** Stasi poison - not described in literature; from Angola - plausible; **latency period:** no; **dose:** no; **cause of death:** respiratory paralysis - not described in literature; **route of administration:** oral; **P:** 2; **D:** 3 | Comparison with “The outgoing Schupo” as both episodes deal with poison from Euphorbiaceae |
|  |  |  | min: 07:50-08:13 | Cause of death determined in first autopsy report: "Poisoning: mydriasis, consequences of convulsion due to muscle contraction, bloody vomiting before exitus." |  |  |  |  |  |
|  |  |  | min: 30:12-30:36 | Chemical-toxicological test results: "significant increase of leucocytes, hepatic necrosis, renal failure"- Suspected intoxication becomes more likely. |  |  |  |  |  |
|  |  |  | min: 30:40-31:55 | Description of the substance: "Spurge family (Euphorbia venena): Milk sap contains a toxin, i.e. a protein, which is highly toxic even in very small quantities; the toxin is difficult to detect in blood if not specially searched for; the final toxicological report is pending. |  |  |  |  |  |
|  |  |  | min: 33:13-34:29 | Description by forensic pathologist: "slow, agonizing death; within 24 h first nausea, then vomiting, diarrhea and cramping abdominal pain; finally, the poison paralyzes the entire body - respiration and other organs fail"; poison difficult to obtain and to produce; experiments in the Eastern Bloc in the mid-20th century until the 1980s; poison possibly originating from the Stasi (former state police in East Germany) |  |  |  |  |  |
|  |  |  | min: 39:25 | Former name of the poison: Norman herb |  |  |  |  |  |
|  |  |  | min: 39:29-39:47 | In the savannahs of Angola highest population of the special spurge; was imported from Angola and thus came to the Normans |  |  |  |  |  |
|  |  |  | min: 39:55-40:25 | Cause of death by Stasi poison confirmed |  |  |  |  |  |

| *Tatort* episode | First broadcast | Location | Time period | Action | Active ingredient/group of active ingredients | Mechanism of action/symptoms | Literature | Plausibility/Detailedness | Time comparison |
| --- | --- | --- | --- | --- | --- | --- | --- | --- | --- |
| *Licorice (1107) (5)* | 03.11.2019 | Münster | min: 01:20 | Dead body found -> market manager Hannes Wagner | **Potassium cyanide (KCN):** cyanide of prussic acid (HCN) (2); fast acting (2); uses: ship and room fumigation, soil sterilization, metallurgy, electroplating, insecticide, rodenticide (2); also in almonds, apricots -> odor of bitter almond (2); **Cannabis:** Active ingredient tetrahydrocannabinol (1); pharmacological effect varies as a function of dose (1); **Ecstasy:** 3,4- metylenedioxy-methamphetamine (MDMA): used as "truth drug" in World War 2 (4); abuse popular in 1980s (3) | **KCN:** blocks cytochrome oxidases by forming a very stable complex with an iron(III) atom of this enzyme (2); disruption of the respiratory chain and interruption of metabolic energy production -> death of the cell (2); initial symptom: hyperpnea, headache, then reddish skin discoloration due to arterialization of venous blood (2); further symptoms: dizziness, convulsions (2); cause of death: respiratory paralysis (2); **Cannabis:** acts via cannabinoid receptors in the brain (1); impairs cognitive function, reaction time, perception, memory, learning (1); dizziness, feeling of hunger (1); hallucinations and panic possible (1); analgesic, muscle relaxant, antiemetic (1); with chronic abuse: lack of motivation and impaired memory. **MDMA:** inhibition of dopamine and norepinephrine reuptake (4); in higher doses serotoninergic, adrenergic and dopaminergic effects, no hallucination (4); enhancement of positive sensations, higher sociability, higher performance and need to communicate, may switch to anxiety and nervousness (4); "tachycardia, dry mouth, jaw clenching, and muscle pain" (3); high dose: "hallucinations, agitation, hyperthermia, and panic attacks" (3) | **(1)** Hardman et al. (2001), p. 637; **(2)** Hardman et el. (2001), p. 1893; **(3)** Hardman et al. (2001), p. 639; **(4)** Dekant, Vamvakas (2010), pp. 236ff; **(5)** Chahoud (2019) | **KCN: symptoms:** dizziness, headache, skin redness - plausible; **mechanism of action:** no; **substance explanation:** explanation of the term, odor - plausible; **latency:** no; **dose:** no; **cause of death:** no; **route of administration:** oral - plausible; **P:** 1; **D:** 3; **Cannabis: symptoms:** no; **mechanism of action:** analgesic, euphoria, stimulation of appetite - plausible; **substance explanation:** no; **latency:** no; **dose:** no; **cause of death:** no; **route of administration:** oral - plausible; **P:** 1; **D:** 5; **MDMA: symptoms:** sociability, positive sensations - plausible; **mechanism of action:** no; **substance explanation:** no; **latency:** no; **dose:** no; **cause of death:** no; **route of administration:** oral - plausible; **P:** 1; **D:** 5 | Comparison with "Who digs a pit for others" "Bitter  Almonds", and "Vodka Bitter Lemon" in terms of potassium cyanide presentation; comparison with  "The Eternal Wave"  in terms of  cannabis effect/presentation. |
|  |  |  | min: 07:15-07:30 | Cause of death determined by forensic pathologist: cyanide poisoning. |  |  |  |  |  |
|  |  |  | min: 07:55-08:25 | No indication of poisoned food yet; presumption that it was bought on the weekly market |  |  |  |  |  |
|  |  |  | min: 09:55-11:35 | Odor test for bitter almond in order to identify potassium cyanide; food must have a strong taste; otherwise you will taste cyanide; licorice identified as poisoned food; official order to prohibit sale of licorice. |  |  |  |  |  |
|  |  |  | min: 13:35 | Ingestion of licorice by candy stand owner to prove that it does not contain cyanide. |  |  |  |  |  |
|  |  |  | min: 13:50-14.10 | Asking about symptoms: dizziness, headache; test for cyanide: put licorice into liquid - color change to blue -> test negative, color change to red -> cyanide detection; test is negative |  |  |  |  |  |
|  |  |  | min: 16:33-18:28 | Sale of marijuana products at old people’s home by inspector Thiel's father in order to reduce pain and to stimulate appetite |  |  |  |  |  |
|  |  |  | min: 39:48-40:32 | Description of the last minutes in Mr. Wagner's life; went upstairs to his office and crawled across the floor |  |  |  |  |  |
|  |  |  | min: 46:10-47:19 | Video of retirees: drug effects (people are dancing, have less pain/are free of pain); confiscation of hashish and marijuana in Thiel's father's cab. |  |  |  |  |  |
|  |  |  | min: 56:19-57:00 | "Three analyses of three samples: Licorice machine, soil, and licorice remains in the garbage"; were positive and highly toxic – suspicion that cyanide licorice is produced in the in the manufactory of Mrs. Monika Maltritz. |  |  |  |  |  |
|  |  |  | min: 59:11-1:00:09 | Forensic pathologist reads the diary (was Monika's school math tutor at the time); notes on Maltritz' mother's alleged suicide; "chewing gum on crossbar, black spots between teeth (possibly licorice residues), pink skin with some red round spots (livor mortis, postmortem lividity) – sign of cyanide poisoning or cold." |  |  |  |  |  |
|  |  |  | min: 1:07:11-1:08:15 | Chaos at the weekly market (people are dancing, sociable, talking crazy stuff); green smoothie contaminated with amphetamine |  |  |  |  |  |
|  |  |  | min: 1:09:18-1:09:33 | Ecstasy detected in a smoothie |  |  |  |  |  |
|  |  |  | min: 1:11:47-1:12:21 | Examination of the remains of Monika Maltritz' mother after exhumation; detection of low concentrations of cyanide even after so many years. |  |  |  |  |  |
|  |  |  | min: 1:12:58-1:13:12 | Ecstasy analysis: MDMA content of 95.0% - very high quality, traces of licorice |  |  |  |  |  |
|  |  |  | min: 1:17:52 | Finding of cyanide in the closet of Monika Maltritz’ ex-husband (not the perpetrator) |  |  |  |  |  |

| *Tatort* episode | First broadcast | Location | Time period | Action | Active ingredient/group of active ingredients | Mechanism of action/symptoms | Literature | Plausibility/Detailedness | Time comparison |
| --- | --- | --- | --- | --- | --- | --- | --- | --- | --- |
| *The Eternal Wave (1096) (3)* | 26.05.2019 | Munich | min: 04:39 | Cardboard medicine box (Tonatin) drops out of Mikesch Seifert’s backpack | **Fentanyl patches:** here fictitious brand Tonatin; drug indication: transdermal use for the treatment of severe chronic pain (1); increasing problem with MOR agonists: dependence with transdermal application (1); **Cannabis:** active ingredient tetrahydrocannabinol (2); pharmacological effects vary as a function of the dose (2) | **Fentanyl patch:** full MOR agonist (120 x more potent than morphine, max. efficacy is comparable) (1); inhibition of excitatory neurotransmitter release (1); sedation, potent analgesia (1); highly lipophilic -> rapid onset of action due to rapid penetration of BBB (1); **Cannabis:** acts via cannabinoid receptors in brain (2); impairment of cognitive function, reaction time, perception, memory, learning (2); dizziness, hunger (2); possible hallucinations and panic (2); analgesic, muscle relaxant, antiemetic (2); with chronic abuse: lack of motivation and memory impairment (2) | **(1)** Seifert (2019), pp. 121, 129; **(2)** Hardman et al. (2001), p. 637; **(3)** Kleinert (2019) | **Symptoms:** no; **Mechanism of action:** no; **Substance explanation:** boiling or chewing promotes rapid delivery to the CNS (1); **Latency:** no; **Dose:** no; **Cause of death:** respiratory depression - plausible; **Route of administration:** oral - plausible; Problem: only reference to junkies, not to, e.g., pain patients; **P:** 1; **D:** 6 | Comparison with  "Licorice" with regard to cannabis effect/presentation: much less detailed. Only analgesia is dealt with. |
|  |  |  | min: 12:57-13:06 | Batic and Leitmayr find Tonatin (the label says: for severe to very severe pain) |  |  |  |  |  |
|  |  |  | min: 18:34-18:53 | Storing of cartons with Tonatin |  |  |  |  |  |
|  |  |  | min: 19:35-20:38 | 2,000 boxes of Tonatin per carton; about 50,000-100,000 euros they want to earn; Seifert and his friend Robert Kraut want a professional contact |  |  |  |  |  |
|  |  |  | min: 26:09-26:57 | Discovery of two dead junkies; had Tonatin with them |  |  |  |  |  |
|  |  |  | min: 31:13-32:05 | Cause of death determined by forensic pathologist: "respiratory arrest in both cases (junkies) caused by an overdose of Tonatin; patches contain a strong synthetic analgesic, 100 times more potent than morphine; junkies boil the patches or chew them and thus bypass the retarded mechanism. The effect of the active ingredient occurs immediately." Substitute drug; Three packs of the patches were found, but they do not account for the amount of active ingredient in the blood. Min. 10x as high as in the patch. |  |  |  |  |  |
|  |  |  | min: 36:48-36:52 | Tonatin found without production date and batch number in Seifert's apartment |  |  |  |  |  |
|  |  |  | min: 52:22-52:43 | Manufacturer says the packages without batch number are from a misproduction (dose is 13 times higher) - should have been destroyed, but have been stolen (Robert Kraut works for the company as a temp) |  |  |  |  |  |
|  |  |  | min: 54:26-55:10 | Discovery of cannabis plantation in Mr. Kraut's house (allegedly for grandfather’s personal use for back pain); also for smoking |  |  |  |  |  |
|  |  |  | min: 1:01:26-1:01:40 | Seifert’s friend explains how the patches are liquefied: "The junkies chew the patches, maybe boil them" |  |  |  |  |  |
|  |  |  | min: 1:07:38-1:08:51 | Boiling of the patches |  |  |  |  |  |

| *Tatort* episode | First broadcast | Location | Time period | Action | Active ingredient/group of active ingredients | Mechanism of action/symptoms | Literature | Plausibility/Detailedness | Time comparison |
| --- | --- | --- | --- | --- | --- | --- | --- | --- | --- |
| *A Day Like Any Other (1085) (2)* | 24.02.2019 | Franconia | min: 17:52-18:06 | Report on the connection between the victims and the perpetrator (lawyer Peters): the male victim was a judge and the female university employee was a witness; "complaint of circulating harmful milk through contaminated animal feed"; Peters was the lawyer of Rolf Koch, who had given his cows animal feed poisoned with dioxins. | **Dibenzodioxin:** polychlorinated dibenzo-p-dioxin (PCDD) (1); enters the environment through numerous processes (e.g., waste incineration, production of non-ferrous metals, steel production, motor vehicle exhaust gases, etc.) (1); various mixtures with varying chlorine contents (1); chlorinated dioxins differ greatly in terms of their toxicity (1); most important intake route: consumption of fish and meat products (1); daily Intake currently approx. 30 pg TEQ per person per day (1); human exposure has been declining for approx. 10 years due to reduction of dioxin formation (1); storage especially in adipose tissue and also in breast milk fat (1); half life in humans approx. 6-9 years (1); main excretion via feces (1); most potent representative: 2,3,7,8-tetrachlorodibenzodioxin (TCDD) (1); **Yukon powder:** fictitious | **Dibenzodioxin:** varying acute toxicity (1); although there is no DNA binding and no genotoxicity, it is one of the most potent carcinogens (1); tumor-promoting effect via Ah receptor: High affinity to cytosolic receptor, which is useful for regulating protein expression for carcinogenic hydrocarbons and other pollutants, making them available for excretion (1); toxic effects are also due to Ah-receptor interaction (1); main symptom: emaciation syndrome -> progressive weight loss (possible cause of death) (1); death within up to 8 weeks after single exposure to high dose (1); symptoms of acute poisoning: nausea/vomiting, irritation of upper respiratory tract (1); after weeks of latency, chloracne manifests (1); possible nerve damages, lipid metabolism disorders, liver damage (1); **Yukon powder:** no mechanism because fictitious | **(1)** Aktories et al. (2013), pp. 1005f, 1055f; **(2)** Marka (2019) | **Symptoms:** abortion of a child - not described in literature but possible because of the emaciation syndrome; **mechanism of action:** no; **substance explanation:** no; **latency:** no; **dose:** no; **cause of death:** no; **route of administration:** oral via contaminated food - plausible; **P:** 4; **D:** 4 | Comparison with "Bienzle and the Dream of Happiness" with regard to dibenzodioxin presentation |
|  |  |  | min: 18:06-18:22 | Explanation of the poison dioxin: fungal poison; mold; cows ate poisoned feed and contaminated milk that got into the stores; plaintiff was the Kessler family - the woman was pregnant and consumed milk |  |  |  |  |  |
|  |  |  | min: 18:48-19:23 | (Review) Severe pain in pregnant Mrs. Kessler; hospitalization; Mrs. Kessler was then stable but lost her child. |  |  |  |  |  |
|  |  |  | min: 20:10-20:20 | Rolf Koch self-reported elevated levels in his milk |  |  |  |  |  |
|  |  |  | min: 20:30-20:56 | Lecture of paragraph 44, Food and Feed Act: "If you sell or otherwise circulate poisoned food, you can file a self-report and thus fulfill your duty of care; anything you say can be used against you in court." |  |  |  |  |  |
|  |  |  | min: 1:17:01 | Rolf Koch is sweating (fear; supposed to accelerate, increase the poisoning effect) |  |  |  |  |  |
|  |  |  | min: 1:18:30 | Body found -> Rolf Koch |  |  |  |  |  |
|  |  |  | min: 1:21:39-1:22:05 | Description of Koch's cause of death: Yukon powder (nerve poison) -> poisoning; "poison acts more quickly at high heart rate: anger, fear, excitement." |  |  |  |  |  |
|  |  |  | min: 1:23:17 | Frau Kessler/ Marie Grüber prepares coffee for Koch and poisons him (you only see her putting sweetener in the coffee) |  |  |  |  |  |
|  |  |  | min: 1:23:50-1:25:00 | Mrs. Kessler/ Marie Grüber gives Mr. Kessler poisoned water and both want to commit suicide with it; police prevents it; Mr. Kessler, however, has already drunk some water - slow and heavy breathing, hallucination of moments of pregnancy and others |  |  |  |  |  |

| *Tatort* episode | First broadcast | Location | Time period | Action | Active ingredient/group of active ingredients | Mechanism of action/symptoms | Literature | Plausibility/Detailedness | Time comparison |
| --- | --- | --- | --- | --- | --- | --- | --- | --- | --- |
| *Solstice (1058) (5)* | 13.05.2018 | Black Forest | min: 02:00-02:48 | Collapse to knees, then total collapse-> Sonnhild | **Vacor:** rodenticide (1); contains N-3-pyridylmethyl-N'-p-nitrophenylurea (1); causes insulin-dependent diabetes mellitus (1); pathomechanism of Vacor-induced diabetes still unclear (2) | **Vacor:** no effect on theophylline- or 12-o-tetradecanoylphorbol-13-acetate-induced insulin release (2); assumption that suppression of insulin release is the pathogenesis and that suppression is independent of cAMP and C-kinase (2); experiments with Vacor are investigating the development of severe peripheral neuropathies in humans caused by Vacor: Decreased amplitude of muscle action potential without abnormal conduction velocity in electrophysiological studies on peripheral motor nerve skeletal systems in mice treated with Vacor -> loss of presynaptic vesicles and swollen endoplasmic reticulum in axon terminal (after 3 days) and progressive degenerative changes (3); results suggest that degenerative changes in axon terminal at a neuromuscular junction contribute to peripheral neuropathy (3); development occurs in the early phase of intoxication (3) | **(1)** Esposti et al. (1996); **(2)** Taniguchi et al. (1989); **(3)** Ahn et al. (1998); **(4)** Schumann, Faust (2018); **(5)** Dag (2018) | **Symptoms:** Shortness of breath, drowsiness - plausible (4); **Mechanism of action:** no; **Substance explanation:** no; **Latency:** no; **Dose:** no; **Cause of death:** diabetes - implausible, should read ketoacidosis; **Route of administration:** i.v.- plausible; **P:** 3; **D:** 3. | no |
|  |  |  | min: 03:45-05:55 | Leg wraps for Sonnhild; bedridden; heavy breathing, intense sweating, inability to speak; then death. |  |  |  |  |  |
|  |  |  | min: 08:17-08:50 | Cause of death determined by forensic pathologist: "Untreated ketoacidosis leading to diabetic coma"; although diabetes mellitus type I is the cause of death in the death certificate, the glucohemoglobin level is definitively too high and no C-peptide is detectable -> evidence for lack of insulin administration despite known diabetes; the physician did not call for an ambulance |  |  |  |  |  |
|  |  |  | min: 26:49-27:07 | Check of Sonnhild's medical record: high blood glucose; prescribed insulin therapy appears to be correct; however, no insulin administered on date of death -> no evidence for lacking treatment |  |  |  |  |  |
|  |  |  | min: 29:36-29:45 | Press release about the death of a CI (confidential informant) who belonged to the Homeland Security Squadron: also died of diabetes |  |  |  |  |  |
|  |  |  | min: 30:43-30:58 | Continuing research on the death of the confidential informant: Marco K. was found dead in the apartment of the witness protection program; medical report shows that he had died acutely from diabetes, not clear whether the illness was known or whether it was also caused by lack of insulin administration |  |  |  |  |  |
|  |  |  | min: 1:00:14-1:00:33 | Forensic pathologist: "Diabetes cannot be artificially induced; any external influence can be excluded "-> current state of science |  |  |  |  |  |
|  |  |  | min: 1:05:50-1:06:21 | Torsten Schmidt (Sonnhild's fiancé) takes out a glass vial and a syringe and then burns the vial |  |  |  |  |  |
|  |  |  | min: 1:19:31 | Handing over a file |  |  |  |  |  |
|  |  |  | min: 1:20:02-1:20:23 | Extract of the file: "Vacor, a rat poison that has been banned worldwide since 1979 and has never been approved in Europe, is also immediately fatal to humans when overdosed; at lower doses, it destroys pancreatic ß-cells and chemically produces type 3E diabetes; symptoms identical to hereditary type I diabetes." |  |  |  |  |  |
|  |  |  | min: 1:25:15-1:25:52 | Finding the death body of Torsten Schmidt and a vial with the rat poison |  |  |  |  |  |

| *Tatort* episode | First broadcast | Location | Time period | Action | Active ingredient/group of active ingredients | Mechanism of action/symptoms | Literature | Plausibility/Detailedness | Time comparison |
| --- | --- | --- | --- | --- | --- | --- | --- | --- | --- |
| *In the Blind Spot (1051) (4)* | 11.03.2018 | Bremen | min: 04:02-04:22 | Pensioner Horst Claasen takes a handful of pills with suicidal intent | **Cholinesterase inhibitors:** (2); inhibitors of acetylcholinesterase (2): three groups of inhibitors: reversible (donepezil), carbamylating (physostigmine), and phosphorylating inhibitors (parathion) (2); **glutamate antagonists:** NMDAR antagonists (2); **antidepressants:** treatment of depression (2); these include: alpha2-adrenoreceptor antagonists, nonselective monoamine reuptake inhibitors (NSMRI), monoamine oxidase inhibitors (MAO-I), selective serotonin reuptake inhibitors (SSRI), selective norepinephrine reuptake inhibitors (SNRI), and selective serotonin/norepinephrine reuptake inhibitors (SSNRI) (2); **citalopram:** SSRI (2); **antipsychotics:** drugs used to "eliminate or attenuate psychopathological syndromes and mental illness" (1) | **Cholinesterase inhibitors:** Acetylcholine's residence time is extended (2); intoxication: massive accumulation of acetylcholine -> muscarinic syndrome (2); **glutamate antagonists:** reduction of the sensitivity of the glutamate receptor (2); **antidepressants:** normalization of neurotransmitter deficit (2); acute intoxications common in suicide attempts (1); intoxications differ depending on the substance (1); intoxications with NSMRI: cardiac problems such as tachycardia, AV block, as well as generalized convulsions (1); SSRI: serotonin syndrome with "hyperthermia, nausea, vomiting, confusion, psychomotor agitation, tremor, myoclonus, and possibly convulsions" (1); MAO-I: arousal and agitation (1); **citalopram:** predominantly improves mood (2); does not improve motivation because it has no effect on norepinephrine reuptake (2); blocks serotonin reuptake (2) | **(1)** Aktories et al. (2013), pp. 294, 310; **(2)** Seifert (2019), pp. 78, 332, 358; **(3)** Hell, Böker (2005); **(4)** Koch (2018) | **Symptoms:** Respiratory distress- plausible; **Mechanism of action:** no; **Substance explanation:** antipsychotics Application area - plausible; **Latency:** no; **Dose:** no; **Cause of death:** no; **Route of administration:** oral- plausible; Scenario: plausible, since number of suicides (partly unnoticed) by e.g. tablet overdoses, in seniors or sick people is high. "According to the Federal Statistical Office, about 35% of suicides involve seniors. (...) The suicide rate reaches a peak in old age that exceeds the middle age group by a factor of two" (3); **P:** 1; **D:** 3 | no |
|  |  |  | min: 06:10-06:25 | Mr. Claasen's breathing becomes increasingly slow and strained; speaking becomes increasingly difficult; then he collapses |  |  |  |  |  |
|  |  |  | min: 06:47-07:02 | Mr. Claasen is taken to hospital by an ambulance car; receives oxygen; but remains unconscious |  |  |  |  |  |
|  |  |  | min: 10:39-10:59 | Inspector Inga Lürsen finds a quantity of tablets; the forensic doctor explains that they contain cholinesterase inhibitors and glutamate antagonists-> for moderate to severe Alzheimer's disease; the name of the drug citalopram is visible; there are also high-dose antidepressants and antipsychotics in the box; "With these, one tries to alleviate restlessness, anxiety and aggression of the patients." |  |  |  |  |  |
|  |  |  | min: 23:23-25:00 | Mr. Claasen is in the hospital; he is awake and responsive; a female doctor says: "Half an hour later and he would not have survived this cocktail of pills"; During the conversation he is completely lucid again. |  |  |  |  |  |
|  |  |  | min: 1:23:32-1:25:25 | Mr. Claasen's son has also taken a drug overdose to commit suicide; he is breathing rapidly but is still responsive and clear in his statements; he is then picked up by the ambulance car and seems to be very sleepy on the bed. |  |  |  |  |  |

| *Tatort* episode | First broadcast | Location | Time period | Action | Active ingredient/group of active ingredients | Mechanism of action/symptoms | Literature | Plausibility/Detailedness | Time comparison |
| --- | --- | --- | --- | --- | --- | --- | --- | --- | --- |
| *Rabies (1046) (6)* | 04.02.2018 | Dortmund | min: 01:19-01:30 | Prisoner (Mr. Strecker) lies in a hospital room; gets an infusion and is monitored; increase in heart rate | **Rabies virus (RABV):** Zoonosis occurring worldwide (1); caused by neurotropic viruses (1); viruses of the Rhabdoviridea family, genus Lyssaviruses (1); different virus species (1); transmitted by saliva, from animals suffering from rabies, by a bite or scratch (1); virus is almost 100% fatal as soon as symptoms appear (2); 100% preventable by vaccination -> prompt administration of postexposure prophylaxis (PEP) for humans and complete vaccination coverage of animal reservoirs (2); domestic dogs account for 99% of human infections, resulting in 59,000 annual deaths (2); reservoir in wildlife: Carnivores; hematophagous and insectivorous bats (1); **Turpentine:** "clear, yellow-green, slightly viscous liquid with a fine aromatic odor" (3); balsamic taste (3); harnessed and used because of its many constituents (3); contains essential oils, various resin acids, bitter substances, dyes, water (3); resin effluxes from mainly pines and larches (3); belongs to the balsams (3); softens resins and is used as an additive for various products (varnishes, adhesives, etc.) (3); has traditionally been used as an additive for various products (varnishes, adhesives, etc.).) (3); was traditionally used in folk and veterinary medicine – today, because of its ADRs, internal use is obsolete (3); only used as ointments, gels or similar (3) | **RABV:** causes acute, neurologic, fatal infection (2); after infection, initial short-term local replication (2); migrates from site of exposure to the CNS by centripetal invasion of peripheral nerve and neurons (2); The virus is replicated in the brain, finally leading to symptoms (2); pathogen excretion via saliva (1); incubation period: 5 days to several years (average 2-3 months) (1); the shorter the distance from point of entry to the CNS, the shorter the incubation period (1); clinical symptoms: 1. prodromal phase: uncharacteristic symptoms: headache and muscle pain, loss of appetite, fever, burning, itching, increased pain sensation, fasciculation in the area of the bite (replication in the spinal ganglion and resulting local inflammation) (1); 2. Acute neurological phase: 1. encephalitic form: cerebral functional deficits, possible hydrophobia -> the mere visual or acoustic perception of water triggers severe anxiety -> can lead to restlessness and convulsions, resulting in dysphagia, salivary discharge; 2. paralytic form: Alterations in spinal cord nerves and peripheral nerves-> lead to paresthesias, hypotonic muscle weakness, descending paralysis -> dysphagia and respiratory paralysis (1); 3. Coma: death during coma due to respiratory paralysis or paralysis of cardiac muscles (1); between symptom onset and death about 7-10 days (1); **Turpentine:** contact dermatitis due to irritant effect (4); acute toxicity after i.v. administration: bloody sputum, pneumonia (5); death in case of overdose (5) | **(1)** Series editing „RKI-Ratgeber“ (2020); **(2)** Brunker, Mollentze (2018); **(3)** Bayerische Landesanstalt (2012); **(4)** Booken et al. (2006); **(5)** DIE WELT (2012); **(6)** Zahavi (2018) | **RABV: symptoms:** convulsions, dysphagia, hydrophobia - plausible; **mechanism of action:** no; **explanation of substance:** route of transmission - plausible; **latency:** 6 weeks – possible range; **dose:** no; **cause of death:** no; **route of administration:** prepared knife - possible; 7 days until symptoms occur - implausible because incubation period is unpredictable; inspector becomes intimate with victim although infection is known and she does not know anything about it - implausible; Dr. Zander's good condition after seizures - implausible; **P:** 3; **D:** 1; **Turpentine: symptoms:** sputum - plausible; **mechanism of action:** no; **substance explanation:** poisoning looks similar to RABV infection - plausible with regard to sputum; **latency:** no; **dose:** no; **cause of death:** no; **route of administration:** injected - plausible; **P:** 1; **D:** 5 | Comparison with “Celebration” with regard to the rabies presentation |
|  |  |  | min: 01:43-03:30 | Tachycardia (HF over 117); hemorrhage into sclerae; then HF over 200; convulsions and spitting of foam; BP 190/100; administration of diazepam i.v.; towel placed on face; then cardiac arrest and start of reanimation; administration of 1 mg epinephrine. |  |  |  |  |  |
|  |  |  | min: 06:18-06:20 | Prisoner's health had deteriorated some time before he passed away |  |  |  |  |  |
|  |  |  | min: 06:34-06:46 | An inmate watches someone picking up a bag with drugs from a can of corn in the food storage area |  |  |  |  |  |
|  |  |  | min: 06:55-07:13 | Forensic pathologist’s report: "According to the laboratory report the victim was infected with Rabies virus.”; Cause of infection unclear, as the man has been in custody for 4 years |  |  |  |  |  |
|  |  |  | min: 07:50-08:00 | Explanation of the route of transmission: "The virus is transmitted via saliva and must at least come into contact with blood or mucous membranes". |  |  |  |  |  |
|  |  |  | min: 09:00-09:10 | The man who handled the corn dose before is cornered by his observer and he takes a bag of drugs away from him |  |  |  |  |  |
|  |  |  | min: 10:37-10:47 | A physician Dr. Zander) from the correctional facility expresses suspicion about infection: "I was infected with rabies in the same way". |  |  |  |  |  |
|  |  |  | min: 11:24-13:20 | Dr. Zander says that he will die; he has also been infected with rabies; 6 weeks earlier there was a stabbing in the correctional facility; he and the victim were injured; presumption that the virus was on the knife; first symptoms as with strong flu; Since Dr. Zander, unlike the inmate, was not hit on the neck, but on the hip, he is still alive, because the path to the brain is longer; it takes max. 7 days until first symptoms occur: Inability to swallow, strong vomiting, seizures |  |  |  |  |  |
|  |  |  | min: 19:10-19:37 | Talk with an employee in one of the four laboratories in Dortmund which do research with the rabies virus; this laboratory has the lowest security level -> because they do not work with Ebola/Marburg virus etc.; 2 weeks ago there was a burglary |  |  |  |  |  |
|  |  |  | min: 20:10-20:50 | Laboratory employee explains transport possibilities of the virus: "deep cold or in a nutrient fluid" - If it gets into a wound or on mucous membranes it is infectious; it survives on prepared knife approx. 30 minutes-> very complex; because of the chaos caused by the burglary, it is unclear whether something is missing |  |  |  |  |  |
|  |  |  | min: 21:48-21:50 | Inmate who stole the sachet reports the theft to another inmate |  |  |  |  |  |
|  |  |  | min: 26:16-27:04 | Another person has symptoms: vomiting and seizure-> then death |  |  |  |  |  |
|  |  |  | min: 28:35-29:10 | Dr. Zander reports cause of death of second person: did not die of rabies but was poisoned; has vomited bile at his mouth |  |  |  |  |  |
|  |  |  | min: 37:43 | Dr. Zander kisses the inspector on her cheek |  |  |  |  |  |
|  |  |  | min: 39:04-39:55 | Dr. Zander and inspector get intimate |  |  |  |  |  |
|  |  |  | min: 40:37-41:05 | Forensic pathologist’s report: Cause of death of second victim: poisoning; puncture site on right neck (something must have been injected directly into the vein); head wound -> victim presumably first knocked unconscious and then poisoned; still unclear with which poison |  |  |  |  |  |
|  |  |  | min: 41:00-41:28 | Prisoner asks others to turn off the tap |  |  |  |  |  |
|  |  |  | min: 59:50-59:59 | Prisoner covers his ears and is restless; spasms of the swallowing muscles, foaming at the mouth |  |  |  |  |  |
|  |  |  | min: 1:10:43-1:10:51 | Prisoner lies in sickroom and convulses; infusion |  |  |  |  |  |
|  |  |  | min: 1:10:57 | Dr. Zander has a seizure |  |  |  |  |  |
|  |  |  | min: 1:13:55 | Dr. Zander is doing better and is doing normal conversation |  |  |  |  |  |
|  |  |  | min: 1:14:25-1:14:30 | The second dead man was poisoned with turpentine |  |  |  |  |  |
|  |  |  | min: 1:26:30-1:26:55 | Death of Dr. Zander |  |  |  |  |  |

| *Tatort* episode | First broadcast | Location | Time period | Action | Active ingredient/group of active ingredients | Mechanism of action/symptoms | Literature | Plausibility/Detailedness | Time comparison |
| --- | --- | --- | --- | --- | --- | --- | --- | --- | --- |
| *Thy name be Harbinger (1038) (1)* | 10.12.2017 | Berlin | min: 1:10:19-1:10:43 | Mrs. Tietzsche (former laboratory manager), who is seriously ill, takes an overdose of tablets; chews the tablets and then swallows them with water | no substance naming | no mechanism because no substance named | no literature because no substance named; **(1)** Baxmeyer (2017) | **Symptoms:** no; **mechanism of action:** no; **substance explanation:** no; **latency:** no; **dose:** no; **cause of death:** no; **route of administration:** oral- plausible; former laboratory employee chews up tablets - implausible; **P:** 3; **D:** 6 | no |
|  |  |  | min: 1:20:38-1:20:45 | Message from the hospice that Mrs. Tietzsche has died |  |  |  |  |  |
|  |  |  | min: 1:26:34-1:27:00 | Mrs. Tietzsche's wife says good-bye to the dead woman |  |  |  |  |  |

| *Tatort* episode | First broadcast | Location | Time period | Action | Active ingredient/group of active ingredients | Mechanism of action/symptoms | Literature | Plausibility/Detailedness | Time comparison |
| --- | --- | --- | --- | --- | --- | --- | --- | --- | --- |
| *Evil Ground (1037) (5)* | 26.11.2017 | Hamburg | min: 08:07-08:14 | Disposal of "poison" (this is how the policewoman calls it) by tanker truck and then disposal into the soil; woman in charge of the Norfrack plant explains: “wastewater, which we dispose of properly and in a legal way" | **Benzene:** solvent (4); use: chem. syntheses (4); highly toxic (4); component of fuels (car) (4); **toluene:** solvent (4); **radioactive metals:** waste from industrial plants, diagnostics, therapeutics, "fall out" from atomic explosion and nuclear reactor malfunctions (1); accumulation in body possible (1); conversion to other elements possible by nuclear reactions -> examples: thorium, plutonium, strontium, radium (1); **mercury:** metallic and organic (1); metallic: stable in environment, various sources possible (1); **cyanobacteria/ blue-green algae:** In fresh and brackish water (2); can form blooms if proper climatic conditions prevail (2); certain species produce endo-, cyto-, neuro-, and hepatotoxins -> blooms toxic to animals and humans (2); oral ingestion through consumption of contaminated water or dermal exposure caused by swimming in contaminated water (2) | **Benzene:** ingestion: oral, inhalation (4); toxic effect on hematopoietic system (4); symptoms: dizziness, euphoria, headache, nausea, vomiting, staggering, tremors, paralysis, unconsciousness (4); chronic poisoning: affects CNS and gastrointestinal tract: pallor (anemia), nervousness (4); carcinogenic (4); **toluene:** "CNS- depressant" (4); no blood count changes, not carcinogenic (4); **radioactive metals:** radiation emission and chemical toxicity (1); different tissue damages as a function of pharmacokinetic behavior, type and intensity of radiation (1); **met. mercury:** reaction with free SH groups of proteins (1); strong enzyme inhibition (1); acute poisoning: inhalation -> pneumonia, nausea, vomiting, colic (1); swallowing-> burns of mouth, pharynx, esophagus, or glottis with resulting Quincke's edema and asphyxia, gastroenteritis with electrolytic and protein losses, polyuria, then oliguria and even anuria with uremia (1); 3rd phase is colitis with massive colics and stomatitis (1); chronic -> target organ CNS, inflammatory changes in motor centers lead to intention tremor, irritability, anxiety, sleep disturbances, speech disturbances and concentration and memory weakness, brown lens discoloration (1); **organic:** e.g. methylmercury (1); bind to SH groups in proteins (1); strong neurotoxicity due to rapid CNS uptake (1); acute: "restlessness, psychomotor agitation, tremor, limitations of sensory perceptual quality, convulsions and finally paralytic states" (1); chronic -> encephalopathy stronger than with inorganic mercury (1); diffuse changes in brain (1); **cyanobacteria:** mechanism still unclear (3); symptoms: mild gastrointestinal discomfort or skin irritation (3); different cyanotoxin species with different toxicity and lethality (3) | **(1)** Aktories et al. (2013), pp. 1023f, 1029; **(2)** Thebault et al. (1995); **(3)** van Riel et al. (2007); **(4)** Hardman et al. (2001), p. 1893; **(5)** Bernardi (2017) | **Symptoms:** pale skin - plausible; aggression, inner and outer restlessness, anxiety, irritability - plausible and fits to organic mercury or chronic poisoning with metallic mercury; shortness of breath - could be due to irritation of metallic mercury; skin rash due to cyanotoxin - plausible; **mechanism of action:** carcinogenic - plausible for benzene, but not for all substances; **substance explanation:** no; **latency:** no; **dose:** no; **cause of death:** no; **route of administration:** swimming and swallowing of lake water - plausible because cyanobacteria in the lake have contact with skin. Metallic mercury is swallowed and neurological symptoms occur; Taking of samples without gloves - unprofessional; episode has zombie character - unrealistic; **P:** 3; **D:** 2 | no |
|  |  |  | min: 10:27-10:59 | Youngest nephew of the victim (Naderi family) stares emotionlessly at inspector Grosz and then bites her right wrist |  |  |  |  |  |
|  |  |  | min: 12:14-12:23 | Mrs. Grosz describes the appearance of the little nephew: "Neck had a rash". |  |  |  |  |  |
|  |  |  | min: 15:21-16:42 | Description of the Norfrack plant, where the dead man worked: "They extract the natural gas from depths of up to 5000 meters"; "The problem is that the gas does not come to the surface alone, but together with toxic substances that are bound deep down - benzene, toluene, radioactive substances, mercury. Some of them get directly into the air that we breathe during extraction, causing illnesses ranging from horrible skin rashes to cancer"; "And respiratory problems. But (...) they have to get rid of the broth again (...) they crush it, they just push the poison into old boreholes. Enormous quantities, non-stop. Thousands of tankers per drilling site. But it doesn't end up at the depth where they originally took it out. Once the stuff gets into the groundwater, then good night, then it's over"; one child has severe breathing problems; wife and the 2 children have dark circles under their eyes, pale skin, dull hair and are aggressive. |  |  |  |  |  |
|  |  |  | min: 20:51-21:33 | Several children discover the victim's older nephew in a car; behave very aggressively, laugh provocatively, hit the car, try to get into the car and have very pale skin color |  |  |  |  |  |
|  |  |  | min: 22:22-22:24 | Mrs. Grosz thinks that everyone in the place does not look healthy |  |  |  |  |  |
|  |  |  | min: 23:40-23:42 | It is true that the poison is pressed into boreholes but according to the scientist Henry Fohlen, from the Lower Saxony Office for Mining, only amoebae would be poisoned 1000 meters beneath the soil surface. |  |  |  |  |  |
|  |  |  | min: 27:27-27:46 | The responsible woman from Norfrack denies that the skin rashes have anything to do with the gas production; they are due to the cyanobacteria/blue-green algae from the nearby lake, where people go swimming |  |  |  |  |  |
|  |  |  | min: 28:45 | Severe rash of the victim's brother’s chest is shown |  |  |  |  |  |
|  |  |  | min: 32:26-34:00 | Homeless man has severe shortness of breath and a very hoarse voice; appears very aggressive; appears mentally confused; doctors say a severe form of paranoid schizophrenia |  |  |  |  |  |
|  |  |  | min: 36:53-37:12 | Youngest nephew has fever-> allegedly flu; rash is from blue-green algae -> doctor's certificate as a prove |  |  |  |  |  |
|  |  |  | min: 38:19-38:59 | The dead man suspected that more wastewater had to be transported because a pipe was defective; if defect was not detected, hectoliters of water would permanently infiltrated into the soil -> disaster-> acidified soil would have to be removed after repair |  |  |  |  |  |
|  |  |  | min: 41:36 | Mr. Fohlen does not wear gloves when taking samples from the lake |  |  |  |  |  |
|  |  |  | min: 42:05-42:30 | Mr. Fohlen falls into the lake |  |  |  |  |  |
|  |  |  | min: 56:19-56:50 | Organic farm owner drinks water from the company’s well and says it is clean |  |  |  |  |  |
|  |  |  | min: 58:56 | The woman in charge of Norfrack plant reports that there was a problem with the pipe, but that it has been fixed |  |  |  |  |  |
|  |  |  | min: 1:00:35-1:03:05 | Mr. Fohlen has strong rash, is vomiting and is very agitated; his samples seem to prove that 100 hectoliters of wastewater must have run into the lake -> benzene, toluene, radioactive isotopes, mercury, combinations of heavy metals and substances unknown to him (possible interactions); but also blue-green algae, i.e. cyanobacteria. Maybe the algae have been intentionally added to the lake in order to prevent that people go swimming; "stuff seems to easily overcome the blood-brain barrier, leads to neuronal deficits"-> changes in character; depends on quantity and body weight |  |  |  |  |  |
|  |  |  | min: 1:06:42-1:08:15 | Playroom of youngest nephew is heavily vandalized -> destruction by the child; he has seizures: hitting, biting, repeatedly hits his head on tabletop (since then hearing loss) -> all these symptoms occurred after swimming in lake; doctors suspect psychosis |  |  |  |  |  |
|  |  |  | min: 1:17:39-1:17:52 | Youngest nephew learned to swim in the lake, swallowed water and a few days later symptoms occurred |  |  |  |  |  |
|  |  |  | min: 1:23:06-1:25:00 | Several children are extremely aggressive and try to kill older nephew; put a bag over Mrs. Grosz' head and try to suffocate her -> severe changes in character |  |  |  |  |  |
|  |  |  | min: 1:26:06-1:26:52 | Mr. Fohlen is doing better; expresses doubts about the results of his samples -> presumably blackmailed by Norfrack |  |  |  |  |  |
|  |  |  | min: 1:27:12-1:27:32 | Radio report: Environmental agency will examine the allegations; currently no evidence of serious contamination |  |  |  |  |  |

| *Tatort* episode | First broadcast | Location | Time period | Action | Active ingredient/group of active ingredients | Mechanism of action/symptoms | Literature | Plausibility/Detailedness | Time comparison |
| --- | --- | --- | --- | --- | --- | --- | --- | --- | --- |
| *In the End You Go Naked (1018) (2)* | 09.04.2017 | Franconia | min: 03:17-03:42 | An incendiary device is thrown into a refugee shelter; spreading of fire | **Carbon monoxide (CO):** tasteless, odorless gas (1); source: incomplete combustion processes of organic matter (1); automobile is largest source (1); many suicides and accidents per year (1); blood level (Hb-CO-> carboxyhemoglobin) in smokers 5.9% (1) | **CO:** complex formation with hemoglobin due to high affinity for iron(II)-containing porphyrins and hemoglobin -> blocks oxygen binding site (1); CO binding to hemoglobin is also reversible, but it is much stronger than that to oxygen and thus the site is blocked and the oxygen transport is minimized (1); due to high affinity (250-fold stronger), even low concentrations (approx. 500 ml/m3) in the respiratory air are sufficient to block half of the heme (1); toxic effect depends on Hb-CO level (1); symptoms due to oxygen deficiency (1); target organs: brain and myocardium (1); symptoms depend on Hb-CO level: visual impairment, mild headache, nausea, vomiting, dizziness, syncope, convulsions, coma, respiratory failure (1); death from Hb-CO concentration of 60-70% (1) | **(1)** Hardman et al. (2001), pp. 1880ff; **(2)** Imboden (2017) | **Symptoms:** no; **Mechanism of action:** no; **Substance explanation:** no; **Latency:** "3 breaths", "went very fast"- inaccurate, but reflects rapid progression at high concentrations; **Dose:** no; **Cause of death:** asphyxiation - plausible; **Route of administration:** inhalation - plausible; **P:** 1; **D:** 6 | Comparison with "The Legal Case Reinhardt”, "Stigma", "Fallen Angels", and  "Eight Years Later" with regard to carbon monoxide poisoning |
|  |  |  | min: 04:14-04:31 | Uncertainty about the perpetrator who threw the incendiary device; 3 first-aid persons and 5 persons have to be examined in the hospital for smoke inhalation; one person is still missing-> Neyla Mafany |  |  |  |  |  |
|  |  |  | min: 08:08-08:18 | The door of the storeroom where the victim Neyla Mafany is lying cannot be opened from the inside -> like a trap |  |  |  |  |  |
|  |  |  | min: 14:06-14:17 | Report on cause of death: "Acute smoke inhalation, the victim breathed three times and then the lungs were blocked. It happened very quickly, she suffocated. Further violence is not ascertainable". |  |  |  |  |  |
|  |  |  | min: 23:15-25:57 | Witnesses' report on the fire attack |  |  |  |  |  |
|  |  |  | min: 1:17:18-1:18:05 | Report that the door is equipped with a magnetic lock and that due to the high temperature the neodymium magnetic switch lost its function. Thus, Neyla Mafany could not get out of the room -> no second person who had locked the room |  |  |  |  |  |

| *Tatort* episode | First broadcast | Location | Time period | Action | Active ingredient/group of active ingredients | Mechanism of action/symptoms | Literature | Plausibility/Detailedness | Time comparison |
| --- | --- | --- | --- | --- | --- | --- | --- | --- | --- |
| *Babbeldash (1012) (6)* | 26.02.2017 | Ludwigs-hafen | min: 01:50-01:58 | Injection of a substance (looks like poppy seeds) into a chocolate croissant | **Poppy seeds (Papaver somniferum):** seeds of the opium poppy (5); used in two ways: a) Seeds are taken from dried seed pods and are used as ingredient in food or pressed to yield a light yellow oil. b) The capsule is still unripe and a milky juice is extracted, which is used for opium production (5); in literature only few cases of poppy seed allergy are known (5); but can be confirmed by a positive skin test and IgE detection (5) | **Poppy seed:** Food allergies: pathological reaction to food and/or additives (1); there are different pathomechanisms: immunological reaction type 1 and type 3 and non-immunological reactions (1); type 1 reaction in this case; type 1 immediate type reaction: reaction to the allergen taking place within seconds to minutes (2); prerequisite is (asymptomatic) sensitization (2); repeated allergen contact: mast cell activation and histamine release (2); IgE molecules that are bound to the Fcℇ receptor are bivalently linked by the antigen (2); Tyrosine phosphorylation of the receptor and other proteins triggers a signal transduction cascade finally leading to histamine exocytosis (2); histamine binds to membrane-bound histamine receptors (H1-H4), thereby causing symptoms: urticaria, asthma, laryngeal edema, rhinitis, conjunctivitis, and even anaphylactic shock (2); oral consumption may also cause furry sensations enorally and pharyngeally, dysphagia, dyspnea, and vomiting (3); most emergency kits contain an antihistamine such as cetirizine (second generation H1R antagonist) or diphenhydramine (first generation H1R antagonist) and a cortisone preparation, plus an epinephrine auto-injector (4) | **(1)** Thiel (1991); **(2)** Aktories et al. (2013), p. 201; **(3)** Braun, Kövery (1988); **(4)** Seifert (2019), p. 52; **(5)** Senti et al. (2000); **(6)** Ranisch (2017) | **Symptoms:** foaming at the mouth - plausible due to difficulty in swallowing; shortness of breath and circulatory collapse - plausible; **mechanism of action:** no; **explanation of substance:** no; **latency:** rapid since Mr. Oehlenschläger was able to stand next to her without being noticed - imprecise but plausible; **dose:** no; **cause of death:** no; **route of administration:** oral - plausible; lack of knowledge about the extent of an allergy (in this case Bieni) - plausible; **P:** 1; **D:** 3 | no |
|  |  |  | min: 09:54-10:55 | Theater director Sophie Fetter is found; foaming at the mouth; no pulse palpable. First responders begin resuscitation; notifying emergency medical services; suspect allergy-related symptoms and search for emergency kit |  |  |  |  |  |
|  |  |  | min: 13:42-15:00 | Death notice in newspaper saying that Mrs. Fetter died because the baker had baked "death croissants"; it is suspected that a chocolate poppy seed croissant has caused an allergic shock |  |  |  |  |  |
|  |  |  | min: 16:34-16:40 | Proof that there were poppy seeds in all croissants -> question if they were added accidentally or intentionally |  |  |  |  |  |
|  |  |  | min: 40:20-40:41 | Raw croissant dough delivered by the wholesale baker had only chocolate filling-> the poppy seeds must have been filled in at the bakery on the spot or on the way to the theater or in the theater |  |  |  |  |  |
|  |  |  | min: 43:23-43:45 | It is definitively murder, because the forensic examination revealed that the poppy was injected into the croissant afterwards |  |  |  |  |  |
|  |  |  | min: 53:58-54:20 | Allegedly, the baker Manfred Oehlenschläger did not notice that poppy seed mass is missing from the bucket |  |  |  |  |  |
|  |  |  | min: 1:07:33-1:08:25 | Bieni (one of the actors) admits that he has filled in the poppy seed mixture; he thought that it would just lead to “throat blocking” of Sophie Fetter. And it was just intended to avoid the premiere; he knew about the allergy and made sure the emergency kit was in the bag |  |  |  |  |  |
|  |  |  | min: 1:15:01-1:15:25 | Sascha (victim's husband) finds Sophie Fetter's emergency kit in his locker |  |  |  |  |  |
|  |  |  | min: 1:17:45-1:19:45 | Manfred Oehlenschläger confesses that during the allergic shock (Sophie was having difficulty breathing and went down; salivation) he found the emergency kit in her bag but did not give it to her. He is arrested |  |  |  |  |  |

| *Tatort* episode | First broadcast | Location | Time period | Action | Active ingredient/group of active ingredients | Mechanism of action/symptoms | Literature | Plausibility/Detailedness | Time comparison |
| --- | --- | --- | --- | --- | --- | --- | --- | --- | --- |
| *The outgoing Schupo (1010) (4)* | 05.02.2017 | Weimar | min: 10:02-11:26 | Policeman Ludwig Maria Pohl (Lupo) breathes heavily and collapses; complains of nausea in hospital ("queasy stomach") although he has eaten a lot (5 eggs, bacon and cornflakes); explanation that he has been poisoned with ricin ("ricin is a pretty strong poison"), that there is no antidote and that he therefore will die; he has hallucinations | **Ricin:** From Ricinus communis (2); tree is also called miracle tree (2); belongs to the Euphorbiaceae (2); grows 1-4 m high (2); in Southern Europe it grows wild on roadsides, in central Europe it grows as an annual ornamental plant (2); used because of the oil (which is not poisonous because the poison remains in press residues (1); used as a laxative (1)) and the press residues (animal feed, fertilizer) (2); Seed surrounded by red-brown-black shell and is readily processed into jewelry (2); toxin is a lectin that occurs in the seed (2); toxin is acid-stable (2); consists of two polypeptide chains linked by a disulfide bridge (2); use as a biological warfare agent possible because it can be dispersed as an aerosol in the air (1) | **Ricin:** B-chain serves to anchor to the cell surface and allows entry into the cell (2); later, the chains separate and the A-chain (N-glucosidase) cleaves an adenine residue from the 28S ribosomal RNA, resulting in a blockade of protein biosynthesis (2); one ricin molecule can kill one cell (2); lethal dose in children: approx. 5 seeds, in adults: approx. 20 (2); latency to symptoms: several hours to 2 days (2); symptoms: "extensive necrosis in the walls of the gastrointestinal tract, but also in the liver, kidney, and spleen" (2); results in severe bleeding (2); death within 3-4 days (2); no antidote (2) | **(1)** Dekant, Vamvakas (2010), p. 249; **(2)** Aktories et al. (2013), pp. 1075f; **(3)** Prigent et al. (2017); **(4)** Marka (2017) | **Symptoms:** Shortness of breath in min. 10 - more likely due to gardening; circulatory collapse - plausible as bleeding may already occur; hallucinations - unlikely at the time, more likely with even higher blood loss; disorientation and sweating - plausible due to circulatory derailment; **mechanism of action:** no; **substance explanation:** at 32:36 - plausible; no antidote- plausible; **latency:** 16-24h - plausible; **dose:** no; **cause of death:** no; **route of administration:** oral - plausible; many meals in the hospital - implausible as 1. food restriction would have to be prescribed and 2. appetite is often lower in severe pain; rapid improvement of his condition - implausible; antibody formation - very unlikely but seen in mice (3); **P:** 3; **D:** 1 | Comparison with "One Way Ticket", because both  episodes deal with poison from  Euphorbiaceae: overall quite plausible and there is a lot of information about the substances in both episodes; in both episodes, the  symptoms match reality, but in this episode, due to the rapid improvement of the general condition, deductions have to be made. |
|  |  |  | min: 11:48-12:03 | Due to the high concentration of ricin, only murder can be inferred; must have happened one day before; "latency period is 16-24 hours and maximum survival time is 72 hours" |  |  |  |  |  |
|  |  |  | min: 19:09-19.50 | Lupo is disoriented |  |  |  |  |  |
|  |  |  | min: 28:04-28:15 | Ricin was administered via cocoa -> all cocoa packages were contaminated by injecting the ricin into them |  |  |  |  |  |
|  |  |  | min: 30:26 | Lupo sweats heavily |  |  |  |  |  |
|  |  |  | min: 32:14-32:24 | The lawyer of Lupo's half-sisters says that ricin was very popular in the Middle Ages and that there was a poison garden at Schwanitz Castle. |  |  |  |  |  |
|  |  |  | min: 32:36-33:18 | Discovery of the "miracle tree, Ricinus communis. Belongs to the spurge family"; "For ricin production you need the seed, the poison is in the seed coat, it is a glycoprotein - it belongs to the group of ribosome-inactivating proteins"; it is even a "Gibsonii mirabilis" a dwarf variety that blooms only in summer, but whose seed can be stored. |  |  |  |  |  |
|  |  |  | min: 33:55-34:09 | Finding the castor beans; "One of the most potent poisons in the world. Compared to this poison, the bite of the black mamba is a mosquito bite." |  |  |  |  |  |
|  |  |  | min: 1:07:23-1:07:29 | Finding of a production manual for ricin and the accessories |  |  |  |  |  |
|  |  |  | min: 1:24-29-1:27:49 | Lupo lies in bed; he ate poisoned cake; the cocoa was supposed to be a distraction |  |  |  |  |  |
|  |  |  | min: 1:28:00-1:48:40 | Doctor explains that Lupo has formed antibodies, which is very rare: "It's a miracle. He will survive." |  |  |  |  |  |

| *Tatort* episode | First broadcast | Location | Time period | Action | Active ingredient/group of active ingredients | Mechanism of action/symptoms | Literature | Plausibility/Detailedness | Time comparison |
| --- | --- | --- | --- | --- | --- | --- | --- | --- | --- |
| *Sons and fathers (1009) (6)* | 29.01.2017 | Saarbrücken | min: 07:40-08:04 | Discovery of skin blisters in the back of the knee and elsewhere on the dead body of Dirk Rebmann by inspector Stellbrink  -> he saw symptoms like these before with sleeping pills poisoning. | **Ecstasy: 3,4- metylenedioxy-methamphetamine (MDMA):** used as "truth drug" in World War II (1); abuse popular in 1980s (4); **benzodiazepines:** frequently used drug group (2); **barbiturates:** belong to sedatives, hypnotics and injection narcotics (2); methohexital and thiopental are used as injection narcotics and phenobarbital is used to control seizures (2); **analgesics:** drugs used to treat pain (2); subdivided into opioid analgesics and non-opioid analgesics (2) | **MDMA:** inhibition of dopamine and norepinephrine reuptake (1); in higher doses serotoninergic, adrenergic and dopaminergic effects, no hallucination (1); enhancement of positive sensation, higher sociability, performance and need to communicate, may shift to anxiety and nervousness (1); "tachycardia, dry mouth, jaw clenching, and muscle pain" (4); high dose: "hallucinations, agitation, hyperthermia, and panic attacks" (4); **benzodiazepines:** binding to benzodiazepine binding site at GABAA receptor (2); enhancement of GABAergic inhibition (2); effect of self-limitation -> weak GABA responses of the cell are enhanced but strong ones do not exceed the physiological maximum -> therefore they are not lethal alone but only in combination with other sedatives -> difference from barbiturates (2); anxiolytic, sedative-hypnotic, muscle relaxant, and antiepileptic (2); **barbiturates:** easily pass the blood-brain barrier, rapid onset of action (2); binding to GABAA receptor and enhanced inhibition of excitatory neurotransmission and potentiation of GABAergic inhibitory transmission (2); unlike benzodiazepines, barbiturates in high doses can activate the receptor without GABA being present (2); sedative-hypnotic, antiepileptic, but not analgesic, anxiolytic, muscle relaxant (2); much easier to overdose (2); contraindications e.g. alcohol (intoxication), sleeping pills or analgesics (3); intoxication: coma, respiratory depression (3); **analgesics:** intoxication: unconsciousness, respiratory depression, death by respiratory paralysis (3) | **(1)** Dekant, Vamvakas (2010), pp. 236ff; **(2)** Seifert (2019), pp. 118ff, 310ff, 328; **(3)** Hardma et al. (2001), p. 418, p. 598; **(4)** Hardman et al. (2001), p. 639; **(5)** Holzer (1940); **(6)** Spirandelli (2017) | **Symptoms:** skin blisters (5)- plausible; **mechanism of action:** no; **substance explanation:** no; **latency:** no; **dose:** no; **cause of death:** heart failure - plausible but respiratory paralysis missing; **route of administration:** oral - plausible; statement that it would not have killed healthy people - very critical by downplaying the combined ingestion of substances in unknown doses; ambiguity in substance naming: first "sleeping pills, painkillers and ecstasy" (min: 31:33-32:00), then "ecstasy and benzos" (min: 50:42-50:46) and finally "painkillers and barbiturates" (min: 1:12:30-1:12:50); layperson might think benzodiazepines and barbiturates are the same thing; **P:** 4; **D:** 6 | Comparison with  "Crookes dogs", "The Legal Case Reinhardt”, and "Legacy sites” in terms of barbiturate presentation |
|  |  |  | min: 31:33-32:00 | Notification that Mr. Rebmann was poisoned; cause of death was heart failure from a "cocktail of sleeping pills, painkillers and ecstasy"; taken about an hour before his death; presumption that it would not have killed a healthy person, but that due to the pre-existing heart conditions it ended fatally here |  |  |  |  |  |
|  |  |  | min: 45:24-45:33 | Laboratory examination revealed that traces of the drug-tablet cocktail were found on the lid and thread of the victim's drinking bottle -> substances had been administered via the liquid of this bottle |  |  |  |  |  |
|  |  |  | min: 50:15 | Illicit drugs are found in the house of the Weller family |  |  |  |  |  |
|  |  |  | min: 50:42-50:46 | Pascal Weller says: "Two days before his (Mr. Rebmann’s) death I got him ecstasy and benzos (benzodiazepines)". |  |  |  |  |  |
|  |  |  | min: 51:54-51:59 | Inspector Stellbrink explains that Mr. Rebmann died from the substances that he previously had obtained from Pascal Weller |  |  |  |  |  |
|  |  |  | min: 1:12:30-1:12:50 | The cook Jean Carlino confesses that he stored the painkillers and barbiturates in the medicine cabinet and that he had procured ecstasy (but he is innocent) |  |  |  |  |  |
|  |  |  | min: 1:12:29-1:12:35 | The taste of the energy drink probably masked the taste of the tablets |  |  |  |  |  |
|  |  |  | min: 1:23:02-1:25:00 | Karim Löscher (stepson of the victim) wanted to commit suicide; for this purpose he had taken medication from the medicine cabinet and mixed it with Pascal Weller's ecstasy; then he was surprised by his mother and she poured it into her husband's drink |  |  |  |  |  |

| *Tatort* episode | First broadcast | Location | Time period | Action | Active ingredient/group of active ingredients | Mechanism of action/symptoms | Literature | Plausibility/Detailedness | Time comparison |
| --- | --- | --- | --- | --- | --- | --- | --- | --- | --- |
| *The King of the Gutter (995) (7)* | 02.10.2016 | Dresden | min: 02:47-02:51 | Inspector Karin Gorniak describes three homeless people (Hansi, Platte, Eumel): "They have a BAC of 0.9 % altogether"; one of the three men has problems with his balance. | **Knockout drops:** imprecise term for combination of various substances (1); often sleeping pills, tranquilizers, or party drugs (3); often overdosed (3); odorless and tasteless (3); often abused for sexual offenses or robberies (1); over 100 active ingredients are abused for this purpose (3); examples: barbiturates, benzodiazepines, antihistamines, anticholinergics, ketamine or neuroleptics, party drugs like heroin or gamma-hydroxybutyrate (GHB) (3); GHB: occurs physiologically in the body as a metabolite of the neurotransmitter y-aminobutyric acid (6); other term is "liquid ecstasy" (4); "since 1 March 2002 it has been subject to the German narcotics law" (6); since 1990 it has been abused, starting in the USA (5) | **Knockout drops/GHB:** brain: increase in acetylcholine, dopamine, and opioid peptides (6); thus, influence on cholinergic, dopaminergic, and opioid systems (6); impairment of perception and consciousness until amnesia (2); rapid penetration of BBB (6); in the 1960s it was manufactured to be used as a GABA agonist for anesthesia (6); later, clinical use for anesthesia was restricted because GHB was thought to induce seizures and seemed to be not analgesic enough (6); still in use for treatment of alcohol/opiate dependence and narcolepsy (6); low dose: 0.5-1.5 g causes relaxation and disinhibition (1); medium dose: 1.0-2.5 g euphoria and sexual arousal (1); high dose: >2.5 g "drowsiness, dizziness, deep sleep, coma, respiratory paralysis, death"(1); administration via drinks -> in combination with alcohol the effect is enhanced (1); onset of action: ca. 15 min (1); duration approx. 4 h (1); "nausea, vomiting, disorientation, agitation, myoclonia, bradycardia, respiratory depression, coma, and transient amnesia" (6) | **(1)** FNR-KO-Tropfen-Aerzteinformation.pdf (w.d.); **(2)** Verba (2007); **(3)** Wikipedia authors (2022); **(4)** Trendelenburg, Ströhle (2005); **(5)** Galloway et al. (1997); **(6)** Stein (2003); **(7)** Zahavi (2016) | **Symptoms:** circulatory disturbances and impaired consciousness, vomiting - plausible; **mechanism of action:** no; **substance explanation:** comparison with generic drug - unfortunate because incomprehensible; **latency:** no; **dose:** no; **cause of death:** no; **route of administration:** oral - plausible; the three men should have had more memory problems because of amnesia; unlikely that with 2 unconscious people in the restaurant, no one calls 911; **P:** 3; **D:** 4 | Comparison with "Salut Palu", "The Last Oktoberfest"  and "Murder is the Best Medicine" with  regard to the  presentation of the knockout drops |
|  |  |  | min: 04:48-05:10 | Statement that the three homeless people were at the bar in an Italian restaurant having a drink that a man previously had bought for them |  |  |  |  |  |
|  |  |  | min: 06:40-06:54 | Report that one of the three had to vomit and that the other two were unconscious; "I couldn't do anything anymore." |  |  |  |  |  |
|  |  |  | min: 08:59-10:04 | The Italian reports that Mr. Taubert and the three men were alone in the restaurant; they drank wine and liquor and left together (he was paid for this lie) |  |  |  |  |  |
|  |  |  | min: 23:33-23:45 | Results of blood tests from the homeless people show that their BAC was no more than 0.04 % -> they were practically sober |  |  |  |  |  |
|  |  |  | min: 1:08:58-1:09:08 | Forensic pathologist reports that knockout drops were found in blood samples of three homeless men: "kind of amateurish generic name for pentobarbital" |  |  |  |  |  |
|  |  |  | min: 1:19:54-1:19:57 | Mr. Springer confesses that he put the knockout drops in the drinks; "I'll get money if I clobber them at the Italian restaurant" |  |  |  |  |  |

| *Tatort* episode | First broadcast | Location | Time period | Action | Active ingredient/group of active ingredients | Mechanism of action/symptoms | Literature | Plausibility/Detailedness | Time comparison |
| --- | --- | --- | --- | --- | --- | --- | --- | --- | --- |
| *Celebration (994) (10)* | 25.09.2016 | Münster | min: 09:15-10:05 | Mr. Götz receives a drug from his psychologist, Dr. Adam (at this point it is unknown which drug it is) | **Botulinum toxin:** colloquial term Botox (1); one of the best known and strongest toxins (1); dose of less than one microgram can be lethal (1); formed by bacterium Clostridium botulinum (1); occurs everywhere and belongs to the gas-forming rod bacteria (1); anaerobic bacteria -> grow and produce best in the absence of air and in an environment containing proteins (1); occurs in spoiled foods (e.g. canned food) (1); heat-labile toxin which is destroyed by cooking food (long and hot enough) (1); recent studies report use in patients with depression (2); **Rabies virus (RABV):** zoonosis occurring worldwide (3); caused by neurotropic viruses (3); viruses of the family Rhabdoviridea, genus Lyssaviruses (3); different virus species (3); transmitted by saliva, from animals suffering from rabies, by a bite or scratch (3); virus is almost 100% fatal once symptoms appear (3); 100% preventable by vaccination -> prompt administration of postexposure prophylaxis (PEP) for humans and vaccination coverage of animal reservoirs (4); domestic dogs account for 99% of human infections, resulting in 59,000 annual deaths (4); reservoir in wildlife: carnivorous, hematophagous, and insectivorous bats (3); **Exogenous insulins:** anabolic hormone for glucose homeostasis (5); used in the treatment of type 1 diabetes and also in late stages of diabetes type 2 (6); human insulin and biosynthetic analogues are used (6); peptide hormone - therefore no oral use possible (6); long-term therapy: subcutaneous; emergencies: intravenous (6); distinction made between short-acting and long-acting insulins (6); due to increase in diabetic drugs and thus insulin therapy, homicides and suicides with insulin are also increasing (7) | **Botulinum toxin:** inhibition of acetylcholine release from presynaptic cells (1); latency period after consumption of contaminated food: 12 hours to 2 days (1); symptoms: "dry mouth, impaired vision, speech and swallowing, ptosis of eyelids, muscle weakness in neck and extremities" (1); high intoxication: death by cardiac arrest or respiratory paralysis after 2-10 days (1); study reports have indicated antidepressant effects after injection into the "anger muscles" such as procerus muscle (2); **RABV:** causes acute, fatal neurological infection (4); after infection, initially short-lived local replication (4); travels from exposure, by invading peripheral nerve fibers, centripetally to CNS along neurons (4); replication of virus occurs in CNS, causing symptoms (4); pathogen excretion via saliva (3); incubation period: 5 days to several years (average 2-3 months) (3); the closer the exposure site to the CNS, the shorter the incubation period (3); clinical symptoms: 1. Prodromal stage: uncharacteristic symptoms: headache and muscle pain, loss of appetite, fever, burning, itching, increased pain sensation, fasciculation in the area of the bite wound (replication in the dorsal root ganglion and resulting local inflammation) (3); 2. Acute neurological stage: 1. encephalitic form: cerebral functional deficits, hydrophobia possible -> visual or acoustic perception of water alone triggers severe anxiety-> can then lead to restlessness and convulsions, resulting in dysphagia, salivary discharge; 2. paralytic form: Change in spinal cord nerves and peripheral nerves-> followed by paresthesias, hypotonic muscle weakness, descending paralysis-> dysphagia and respiratory paralysis (3); 3. coma: death during coma due to respiratory paralysis or paralysis of cardiac muscles (3); approx. 7-10 days between symptom onset and death (3); **exogenous insulins:** most important ADR in intoxication: hypoglycemia (6); unconsciousness follows if the early signs of hypoglycemia are not recognized, thus increasing the risk of aspiration (6); in the course of time, irreparable brain damage and circulatory arrest may occur due to the glucose deficiency (5); in persons who do not have diabetes, hyperglycemia may rarely occur in intoxication due to the body's counterregulation (5) | **(1)** Dekant, Vamvakas (2010), pp. 253f; **(2)** Finzi et al. (2018); **(3)** Series editing „RKI-Ratgeber“ (2020); **(4)** Brunker, Mollentze (2018); **(5)** Datenblatt: Vergiftung-Antidiabetika (Insulin) (2021); **(6)** Seifert (2019), pp. 244, 246f; **(7)** Bottinelli et al. (2020); **(8)** Al-Chalabi, Hardiman (2013); **(9)** Kasper (1912); **(10)** Jessen (2016) | **Symptoms:** progressive paralysis, death by respiratory paralysis - resembles ALS - plausible (8); hysterical paresis - plausible (9); **mechanism of action:** no; **substance explanation:** botulism triggered by botulinum toxin - plausible; botulinum toxin is used in research for treatment of depression - plausible; **latency:** several minutes - implausible; if it were rabies- even more implausible; **dose:** no; **cause of death:** failure of respiratory muscles - plausible; **route of administration:** oral - plausible; doctors think rabies is airborne - implausible; insulin overdose lethal and pleasant due to unconsciousness - plausible; **P:** 3; **D:** 2 | Comparison with  "Rabies" with regard to the rabies  presentation: detailed symptom presentation although it is not a  rabies virus infection here; In this episode, the latency period is incorrect -> in "Rabies", however it is correct; In this episode, the focus is on paralysis. In the other episode, the  "better known"  symptoms such as  salivary discharge disorder etc. are shown; The transmission route  is explained correctly in "Rabies"; here it is implausible that doctors believe that it can be transmitted via the air. |
|  |  |  | min: 23:21-23:42 | Mr. Götz draws up a syringe with medicine and injects it into the food |  |  |  |  |  |
|  |  |  | min: 26:22-26:40 | Prof. Boerne gets a snack from Mr. Götz and eats it |  |  |  |  |  |
|  |  |  | min: 30:18-30:46 | Mr. Götz says that he has poisoned a snack (Boerne's) with a drug that simulates the symptoms of ALS; death will be like that of ALS patients; |  |  |  |  |  |
|  |  |  | min: 33:27-33:50 | One of the guests (Prof. Papst) says that he can no longer feel his right leg and that it is probably paralyzed -> he suspects that these are the first symptoms of ALS. |  |  |  |  |  |
|  |  |  | min: 40:05-41:00 | Prof. Papst now still expresses dizziness and paralysis symptoms in his left leg; Boerne looks at his hands. He feels symptoms in his hands; Boerne thinks that the other guest has hysterical paresis; Boerne has balance problems |  |  |  |  |  |
|  |  |  | min: 41:21-42:00 | Boerne's eye muscles begin to fail -> he has heavily dilated pupils; he can no longer see properly and move his eyelids |  |  |  |  |  |
|  |  |  | min: 44:27-46:28 | Boerne tests his mouth and tongue muscles; he speaks haltingly and stutters; says his acetylcholine secretion is inhibited; symptoms are very similar to ALS; Prof. Papst lies on the floor and coughs and is afraid not to know when he will suffocate; Boerne's diagnosis is botulism; an antitoxin would be necessary; the paralysis spreads very quickly |  |  |  |  |  |
|  |  |  | min: 47:30-47:40 | Dr. Adam's patient file on Mr. Götz states that he wants to use a deadly virus against Boerne and his colleagues |  |  |  |  |  |
|  |  |  | min: 49:51-50:21 | Boerne sees double images and sweats profusely |  |  |  |  |  |
|  |  |  | min: 52:13-53:15 | In the laboratory of Mr. Götz, samples presumably from Rabies virus are found; paralytic rabies resembles ALS (paralysis of extremities and facial muscles); "You have to be bitten first" -> "Not in times of laboratory research. If you have a high virus titer, a tiny lesion in the oral mucosa is enough to transmit it. Manifest rabies is fatal in any case." |  |  |  |  |  |
|  |  |  | min: 54:56-56:10 | Boerne has paralysis of the neck muscles and can no longer lift his head; according to Mr. Götz, paralysis of the arms, legs and finally of the internal organs will follow |  |  |  |  |  |
|  |  |  | min: 57:15-57:23 | Dr. Adam hopes it is rabies; she reports that the immunoglobulin is injected intramuscularly |  |  |  |  |  |
|  |  |  | min: 58:05-58:08 | Prof. Papst is apparently feeling better again |  |  |  |  |  |
|  |  |  | min: 59:23-59:31 | Mr. Götz wants the other hostages to kill Boerne, since he is going to die anyway; he says that the longer they are in a room with Boerne, the greater the risk of infection |  |  |  |  |  |
|  |  |  | min: 1:02:05-1:03:05 | One of the hostages would have enough insulin to kill Boerne; it would be a gentler death; symptoms look like paralytic rabies and so he would die anyway and the lives of the others would be saved |  |  |  |  |  |
|  |  |  | min: 1:05:54-1:06:45 | Boerne is supposed to get a rabies vaccine and an immunoglobulin, but his assistant says that in his condition it wouldn't do any good anyway, and he himself doesn't think it's rabies; Mr. Götz confirms that it's not rabies; Boerne's saliva runs out of his mouth and he collapses. |  |  |  |  |  |
|  |  |  | min: 1:07:49-1:08:20 | The laboratory confirms that it is not rabies |  |  |  |  |  |
|  |  |  | min: 1:12:06-1:12:22 | Finding of a study: Botox against depression; "Botox causes a relaxation of the forehead muscles and the brain responds to this measure with a relaxed emotional state." |  |  |  |  |  |
|  |  |  | min: 1:13:12-1:13:33 | Confirmation that Botox is a neurotoxin with paralyzing effects, which is not contagious; a bacterium in spoiled food produces the toxin |  |  |  |  |  |
|  |  |  | min: 1:14:56-1:15:25 | Finding many bottles of Botox in Dr. Adam's office; "Botox is a neurotoxin. If ingested rather than injected, it causes botulism. Progressive paralysis until respiratory muscle failure." |  |  |  |  |  |
|  |  |  | min: 1:20:45-1:21:50 | Boerne has hallucinations and is unconscious |  |  |  |  |  |
|  |  |  | min: 1:24:24-1:24:38 | Inspector Thiel reports that Boerne is on the path of recovery thanks to an antitoxin; however, he is still connected to the heart-lung machine |  |  |  |  |  |
|  |  |  | min: 1:25:17-1:25:27 | Report that Boerne is well again after a few days only |  |  |  |  |  |

| *Tatort* episode | First broadcast | Location | Time period | Action | Active ingredient/group of active ingredients | Mechanism of action/symptoms | Literature | Plausibility/Detailedness | Time comparison |
| --- | --- | --- | --- | --- | --- | --- | --- | --- | --- |
| *The last Oktoberfest (956) (9)* | 20.09.2015 | Munich | min: 04:55-06:10 | Inspector Leitmayr discovers a young man at a subway station who is sweating profusely, has a restricted mental state, is sitting on the floor and cannot get up -> possibly drunk | **Knockout drops:** imprecise term for a combination of different substances (3); often sleeping pills, tranquilizers, or party drugs (5); often overdosed (5); odorless and tasteless (5); often abused for sexual offenses or robberies (3); over 100 active ingredients are abused for this purpose (5); examples: barbiturates, benzodiazepines, antihistamines, anticholinergics, ketamine or neuroleptics, party drugs like heroin or gamma-hydroxybutyrate (GHB) (5); **GHB:** naturally occurring in the body, metabolite of the neurotransmitter y-aminobutyric acid (8); other term is "liquid ecstasy" (6); "since 1 March 2002 it has been subject to the German Narcotic Drugs Act" (8); since 1990 it has been abused, starting in the USA (7); **alcohol:** ethyl alcohol or ethanol, EtOH (1); drinks with ethanol belong to addictive drugs (1) | **Knockout drops/GHB:** Increase in acetylcholine, dopamine, and opioid peptides (8); impairment of perception and consciousness to the point of amnesia (4); rapid penetration of BBB (8); in the 1960s it was manufactured to be used as a GABA agonist for anesthesia (8); clinical use for anesthesia then curtailed because it is thought to be seizure- inducing and not analgesic enough (8); possible use in alcohol/opiate dependence and narcolepsy (8); small dose: 0.5-1.5 g causes relaxation and disinhibition (3); medium dose: 1.0-2.5g euphoria and sexual arousal (3); high dose: > 2.5g "sleepiness, drowsiness, deep sleep, coma, respiratory paralysis, death"(3); administration via drinks -> enhancement of effect in combination with alcohol (3); onset of action: approx. 15 min (3); duration approx. 4 h (3); "nausea, vomiting, disorientation, agitation, myoclonia, bradycardia, respiratory depression, coma, and transient amnesia" (8); **alcohol:** acute toxicity and dose-dependent CNS effects (1); low doses: stimulant (1); high doses: toxic and narcotic (2); "neurotoxic (nerves), hepatotoxic (pancreas), and cardiotoxic (heart) effects (2); initially nausea with vomiting, hypoglycemia with impaired consciousness, hyperventilation, and hypothermia (1); followed by rise in blood pressure, red, dry, and hot skin, and a decrease in muscle performance (1); in very severe intoxications: respiratory depression (1) | **(1)** Hardman et al. (2001), pp. 429ff; **(2)** Bützer (2016); **(3)** FNR-KO-Tropfen-Aerzteinformation.pdf (w.d.); **(4)** Verba (2007); **(5)** Wikipedia authors (2022); **(6)** Trendelenburg, Ströhle (2005); **(7)** Galloway et al. (1997); **(8)** Stein (2003); **(9)** Kren (2015) | **Symptoms:** sleepiness, drowsiness, collapse, unconsciousness, impaired consciousness, vomiting - plausible; **mechanism of action:** no; **substance explanation:** similarity of GHB and alcohol intoxication – plausible; rapid CNS penetration, former anesthetic, problem with alcohol combination - plausible; **latency period:** no; **dose:** 400µg lethal - if micrograms per milliliter then plausible; **cause of death:** can also be secondary; e.g. choking on vomit – plausible. **route of administration:** oral - plausible; GHB often abused as a knock-out drops, but knockout drops do not always contain GHB- should have been explained; **P:** 2; **D:** 2 | Comparison with "Salut Palu," "The King of the Gutters," and "Murder is the Best medicine” with regard to the  presentation of  knockout drops |
|  |  |  | min: 07:47-07:50 | The inspector explains that man died because he choked on his vomit |  |  |  |  |  |
|  |  |  | min: 10:16-10:20 | A man crushes a glass bottle at the Oktoberfest fairground |  |  |  |  |  |
|  |  |  | min: 18:14-18:30 | A waitress says that she served the man only two big glasses of beer. After that they consumed only non-alcoholic drinks |  |  |  |  |  |
|  |  |  | min: 19:30-19:53 | Autopsy report states that the man had only 0.07 % BAC; the forensic pathologist is asked to do a tox screen |  |  |  |  |  |
|  |  |  | min: 20:15-20:51 | Another man collapses at the Oktoberfest fairground and is unconscious |  |  |  |  |  |
|  |  |  | min: 21:17-21:30 | Inspector Batic reports GHB poisoning at the Oktoberfest |  |  |  |  |  |
|  |  |  | min: 21:45-22:15 | The forensic report confirms GHB poisoning; there are probably 15 other cases |  |  |  |  |  |
|  |  |  | min: 22:20-22:55 | The forensic pathologist explains: "GHB floods within a few minutes. It used to be an anesthetic until they took it off the market because it's too dangerous. Now the kids take it because it makes you high. But if you take too much of it, it knocks you out. Especially in combination with alcohol."; the Italian didn't die from it because he was young; "If you have someone with high blood pressure (...). You can be glad that it has not yet "crumbled" someone. |  |  |  |  |  |
|  |  |  | min: 28:36-28:40 | "GHB is extremely dangerous when overdosed, especially when combined with alcohol." |  |  |  |  |  |
|  |  |  | min: 32:33-32:45 | Another guest collapses and sweats profusely; is unresponsive |  |  |  |  |  |
|  |  |  | min: 32:52-33:00 | The guest has a heart defect and therefore had such a strong reaction to the GHB; but he is still alive |  |  |  |  |  |
|  |  |  | min: 38:35-39:55 | Inspector Batic's aunt is unresponsive and vomits -> not GHB but alcohol intoxication |  |  |  |  |  |
|  |  |  | min: 49:36 | The radio reports on the incidents; here the name k.o. drops is used |  |  |  |  |  |
|  |  |  | min: 56:08-56:50 | Reanimation of another guest; but eventually he dies |  |  |  |  |  |
|  |  |  | min: 57:15-57:28 | Forensic report on the last dead man: 1500 micrograms, 400 can already be fatal |  |  |  |  |  |
|  |  |  | min: 1:19:05-1:19:10 | Finding the manufacturing site/small laboratory in Gränsel's apartment |  |  |  |  |  |
|  |  |  | min: 1:19:45-1:19:48 | Finding Gränsel's body; the forensic pathologist suspects that he overdosed himself |  |  |  |  |  |

| *Tatort* episode | First broadcast | Location | Time period | Action | Active ingredient/group of active ingredients | Mechanism of action/symptoms | Literature | Plausibility/Detailedness | Time comparison |
| --- | --- | --- | --- | --- | --- | --- | --- | --- | --- |
| *Murder is the Best Medicine (917) (14)* | 21.09.2014 | Münster | min: 01:32-02:15 | A man (pharmacist Andreas Hölzenbein) is attacked on a park bench: he is injected with something into his right thigh. Then he collapses and is subsequently resuscitated (with defibrillation); a girl tells the emergency service that she saw the incident | **Exogenous insulins:** anabolic hormone for glucose homeostasis (1); used in the treatment of type 1 diabetes and also in late phases of type 2 diabetes (2); human insulin and biosynthetic analogues are used (2); peptide hormone; therefore, no oral administration is possible (2); continuous therapy: subcutaneous administration; emergencies: intravenous administration (2); difference between short-acting and long-acting insulins (2); because of the increase in diabetic drugs and thus insulin therapy, homicides and suicides with insulin are also increasing (3); **K.o. drops:** inaccurate term for a combination of different substances (4); often sleeping pills, tranquilizers, or party drugs (6); often overdosed (6); odorless and tasteless (6); often abused for sexual offenses or robberies (4); over 100 active ingredients are abused for this purpose (6); examples: barbiturates, benzodiazepines, antihistamines, anticholinergics, ketamine or neuroleptics, party drugs such as heroin or gamma-hydroxybutyrate (GHB) (6); **GHB:** occurs naturally in the body, metabolite of the neurotransmitter y-aminobutyric acid (9); other term is "liquid ecstasy" (7); "Since 1 March 2002 it has been subject to the German Narcotic Drugs Act" (9); since 1990 it has been abused, starting in the USA (8); **amphetamines:** synthetic drugs (10); dextroamphetamine (D-amphetamine) is a stronger derivative (10); taken as powder or injection (10); until 1950, amphetamines were used for psychiatric diseases, colds or to suppress appetite (10); due to high dependence potential: Narcotics Act (10); basic structure of phenylethylamine (10); in drug scene, phenylethyl derivatives are called, among others, "speed" (10); **modafinil:** belongs to the class of psychostimulant drugs (11); approved for therapy of excessive sleepiness in the course of narcolepsy (11); **propranolol:** ß1 and ß2 receptor antagonist (12); use in the therapy of cardiovascular diseases such as hypertension (12); **midazolam:** drug class of benzodiazepines (2); **Zytarix:** fictitious | **Exogenous insulins:** most important ADR in intoxication: hypoglycemia (2); unconsciousness, if the early signs of hypoglycemia are not recognized, thus increasing the risk of aspiration (2); in the course of time, irreparable brain damage and circulatory arrest may occur due to the glucose deficiency (1); in persons who do not have diabetes, hyperglycemia may rarely occur in intoxication due to the counter-regulation of the body (1); **knockout drops/GHB:** Increase in acetylcholine, dopamine, and opioid peptides (9); impairment of perception and consciousness to the point of amnesia (5); rapid penetration of the BBB (9); in the 1960s it was manufactured to be used as a GABA agonist for anesthesia (9); clinical use for anesthesia then curtailed because it is thought to be seizure- inducing and not analgesic enough (9); possible use in alcohol/opiate dependence and narcolepsy (9); small dose: 0.5-1.5 g causes relaxation and disinhibition (4); medium dose: 1.0-2.5 g euphoria and sexual arousal (4); high dose: >2.5 g "sleepiness, drowsiness, deep sleep, coma, respiratory paralysis, death"(4); administration via drinks-> in combination with alcohol the effect is enhanced (4); onset of action: approx. 15 min (4); duration approx. 4 h (4); "nausea, vomiting, disorientation, agitation, myoclonia, bradycardia, respiratory depression, coma, and transient amnesia" (9); **amphetamines:** release of norepinephrine and dopamine (10); norepinephrine has centrally stimulating effect, and dopamine has an euphoric effect (10); very high doses: hallucinations and "psychotic states with delusions," due to very strong dopamine release (10); **modafinil:** centrally acting sympathomimetic (11); inhibition of dopamine reuptake (11); thereby promoting wakefulness (11); **propranolol:** inhibition of epinephrine and norepinephrine action by blocking ß-receptors (12); thereby lowering heart rate, blood pressure, and relieving cardiac stress (12); **midazolam:** allosteric modulation at GABAA receptor (2); sedative-hypnotic and antiepileptic (2) | **(1)** Datenblatt: Vergiftung-Antidiabetika (Insulin) (2021); **(2)** Seifert (2019), pp. 244, 246f, 312; **(3)** Bottinelli et al. (2020); **(4)** FNR-KO-Tropfen-Aerzteinformation.pdf (w.d.); **(5)** Verba (2007); **(6)** Wikipedia authors (2022); **(7)** Trendelenburg, Ströhle (2005); **(8)** Galloway et al. (1997); **(9)** Stein (2003); **(10)** Dekant, Vamvakas (2010), pp. 235f; **(11)** Walliczek-Dworschak (2019); **(12)** Hardman et al. (2001), p. 249; **(13)** Wolter (2016); **(14)** Jauch (2014) | **Symptoms:** circulatory collapse - plausible; retrograde amnesia of midazolam – wrong, it causes anterograde amnesia (2); wakefulness promoted by amines and modafinil - plausible; knockout drops: defenseless - plausible **Mechanism of action:** no; **Substance explanation:** fishy odor of amines - plausible (10); **Latency period:** immediate collapse of Mr. Hölzenbein - implausible; **Dose:** no; **Cause of death:** cardiac arrest- plausible; **Route of administration:** knockout drops: oral - plausible; insulin: injected - plausible; amphetamines: snorted - plausible; midazolam: oral - plausible; more counterfeits because of online drug trade - plausible (13); **P:** 2; **D:** 3 | Comparison with "Salut Palu", "The King of the Gutter" and "The Last Octoberfest” " with regard to the knockout drops;  comparison with  "Celebration"  with regard to the  exogenous insulin: in both, the  effect was not discussed; both mention lethality in case of overdose; here, poisoning with insulin was carried out, in the other only mentioned |
|  |  |  | min: 08:30-09:15 | A doctor from the hospital excludes external influence in case of cardiac arrest; Prof. Boerne finds a puncture site on the sartorius muscle; he insists on a toxicological examination; the doctor justifies himself and says that such an examination is unnecessary in case of admission to hospital due to ventricular fibrillation. |  |  |  |  |  |
|  |  |  | min: 10:24-10:50 | The paramedic reports that the man was already unconscious when the emergency serviced arrived at the park |  |  |  |  |  |
|  |  |  | min: 13:54-14:18 | Mr. Hölzenbein, who is intubated, is administered a substance via the central venous catheter; he then suffers a cardiac arrest |  |  |  |  |  |
|  |  |  | min: 14:44-14:58 | Boerne reports that Hölzenbein's blood test showed no appreciable abnormalities |  |  |  |  |  |
|  |  |  | min: 15:19-15:25 | According to Boerne, adrenaline, insulin and gamma-hydroxybutyric acid are examples of substances that quickly become undetectable |  |  |  |  |  |
|  |  |  | min: 22:35-22:45 | A doctor from the hospital thinks that after such serious tachycardia, heart failure is not uncommon |  |  |  |  |  |
|  |  |  | min: 27:05-27:50 | Hözenbein says that before his attack, he analyzed a sample with HPLC (high performance liquid chromatography), which is very expensive and time-consuming; he analyzed "Zytarix"; a highly toxic substance used as an infusion in chemotherapy; the preparation is always individually prepared on the day of treatment; "If there is too much poison in it, the patient dies, if there is too little, it doesn't help." |  |  |  |  |  |
|  |  |  | min: 29:32-29:43 | Boerne reports that his bedmate has no complaints, despite high-dose chemotherapy with Zytarix |  |  |  |  |  |
|  |  |  | min: 36:38-37:03 | Boerne suspects that the drug Zytarix is counterfeit; this would not be difficult, as it is simply a matter of putting glucose into a different package; |  |  |  |  |  |
|  |  |  | min: 42:00-42:17 | The forensic pathologist found nothing; there is also no evidence of organic heart failure; "No abnormalities and toxicology was also negative" |  |  |  |  |  |
|  |  |  | min: 43:40-44:42 | Dr. Süßmilch reported that she also suspected fraud with Zytarix; Dr. Knapp (hospital pharmacist) did not want to show the analyses and only said that everything was normal; she had then hired Hölzenbein; Hölzenbein confirmed that only a subtherapeutic dose of the active ingredient is contained in Zytarix; "patients may pay for this mess with their lives". |  |  |  |  |  |
|  |  |  | min: 52:40-53:00 | Dr. Knapp reports on the analysis: Zytarix contains too little active ingredient |  |  |  |  |  |
|  |  |  | min: 53:25-53:34 | Dr. Knapp pours the contents of an ampoule into Dr. Süßmilch's drink |  |  |  |  |  |
|  |  |  | min: 54:34-54:50 | Dr. Süßmilch expresses the suspicion that Dr. Knapp is consuming amphetamines; she then drinks her beverage |  |  |  |  |  |
|  |  |  | min: 55:38-56:02 | Dr. Süßmilch lies unconscious on the floor; inspector Thiel begins resuscitation and then presumably stops from exhaustion |  |  |  |  |  |
|  |  |  | min: 57:20-57:45 | The forensic pathologist says that Dr. Süßmilch had died shortly before Thiel's arrival; no evidence of external influences |  |  |  |  |  |
|  |  |  | min: 1:02:30-1:02:38 | The forensic pathologist could not find anything unusual with Dr. Süßmilch |  |  |  |  |  |
|  |  |  | min: 1:03:36-1:04:05 | Something is injected into a NaCl infusion. Afterward, the label of NaCl is pasted over with that of Zytarix |  |  |  |  |  |
|  |  |  | min: 1:04:10-1:04:20 | Dr. Knapp takes some powder from a small container and snorts it through the nose |  |  |  |  |  |
|  |  |  | min: 1:07:17-1:07:57 | Dr. Knapp wants to stop adulterating the chemotherapeutic drugs; Frank Scheinmann threatens him; he also warns him that he will not get his abusive drugs |  |  |  |  |  |
|  |  |  | min: 1:10:18-1:10:30 | Dr. Knapp puts more tablets into Boerne's dispenser |  |  |  |  |  |
|  |  |  | min: 1:12:23-1:12:40 | Boerne wants a hair from Dr. Knapp checked for D-amphetamine, because he noticed a fishy, ammonia-like odor; symptoms probably indicate "speed"; "In World War 2, amphetamine was used to keep soldiers awake, motivated and aggressive." |  |  |  |  |  |
|  |  |  | min: 1:20:57-1:22:00 | Dr. Süßmilch was found to have a puncture site under her hair; in the surrounding tissue, the C-peptide of a human insulin was found, which has a longer half-life than the patient's own insulin; cause of death is therefore an insulin injection; Boerne suspects that she was previously administered gamma-hydroxybutyric acid (knockout drops) to render her defenseless; Dr. Knapp took many drugs: modafinil, propranolol and amphetamines-> to keep him from getting tired; the additional drugs in Boerne's dispenser were 30 mg of midazolam-> not lethal but it does provide retrograde amnesia |  |  |  |  |  |
|  |  |  | min: 1:25:39-1:25:47 | Dr. Knapp breaks an ampoule again and pours the contents into Mia's drink -> also knockout drops |  |  |  |  |  |

| *Tatort* episode | First broadcast | Location | Time period | Action | Active ingredient/group of active ingredients | Mechanism of action/symptoms | Literature | Plausibility/Detailedness | Time comparison |
| --- | --- | --- | --- | --- | --- | --- | --- | --- | --- |
| *The Legal Case Reinhardt (905) (3)* | 23.03.2014 | Cologne | min: 02:27-03:09 | Three burned children's bodies are found in the house of Reinhardt family | **Barbiturates:** belong to sedatives, hypnotics and injectable narcotics (1); methohexital and thiopental used as injectable narcotics and phenobarbital used for its anticonvulsant effect (1); **carbon monoxide (CO):** tasteless, odorless gas (2); source: incomplete combustion processes of organic matter (2); car is largest source (2); many suicides and accidents annually (2); blood level (Hb-CO-> carboxyhemoglobin) in smokers 5.9% (2) | **Barbiturates:** Very rapidly cross the BBB, rapid onset of action (1); binding to the GABAA receptor and enhanced inhibition of excitatory neurotransmission and strengthening of GABAergic inhibitory transmission (1); unlike benzodiazepines, they can also cause activation of the receptor in high doses without the presence of GABA (1); sedative-hypnotic, antiepileptic but not analgesic, anxiolytic, muscle relaxant (1); much easier to overdose (1); contraindications e.g. alcohol (intoxication), sleeping pills or painkillers (1); **CO:** complex formation with hemoglobin due to high affinity for iron(II)-containing porphyrins and hemoglobin -> blocks oxygen binding site (2); CO binding to hemoglobin also reversible, but much stronger than that of oxygen and thus the site is blocked and oxygen transport is minimized (2); due to high affinity (250 times stronger) even low concentrations (ca. 500 ml/m3) in the respiratory air are sufficient to block half of the heme (2); toxic effect depends on Hb-CO level (2); symptoms due to oxygen deficiency (2); target organs: brain and myocardium (2); symptoms depend on Hb-CO level: visual impairment, mild headache, nausea, vomiting, dizziness, syncope, convulsions, coma, respiratory failure (2); death from Hb-CO concentration of 60-70% (2) | **(1)** Seifert (2019), pp. 310, 328; **(2)** Hardman et al. (2001), pp. 1880ff; **(3)** Fischer (2014) | **Symptoms:** Cough - plausible if due to CO or attempted suicide by hanging; **Mechanism of action:** no; **Substance explanation:** sleeping pill - colloquial; **Latency:** no; **Dose:** no; **Cause of death:** asphyxiation; **Route of administration:** no; Emergency physician and physicians at hospital find no evidence of attempted suicide - unlikely; **P:** 3; **D:** 6 | Comparison with "Sons and Fathers,"  "Crooked Dogs,"  and "Legacy" in terms  of barbiturate presentation;  comparison with "In the End You Go  Naked," "Burn mark", ”Fallen Angels," and "Eight Years Later" in terms of carbon monoxide poisoning |
|  |  |  | min: 04:08-04:58 | Mrs. Reinhardt is found near a lake, near the house; she appears totally distraught and dazed; she has a severe cough attack; the emergency service provides her with oxygen |  |  |  |  |  |
|  |  |  | min: 05:45-05:48 | According to emergency physician Mrs. Reinhardt suffers from a mild smoke intoxication and severe shock |  |  |  |  |  |
|  |  |  | min: 11:03-11:38 | Statement from psychologist that Mrs. Reinhardt has amnesia, a psychotic episode, in the wake of her shock |  |  |  |  |  |
|  |  |  | min: 12:26-12:45 | Forensic pathologist finds children's position unusual, finds no evidence of asphyxiation panic, and concludes they were either previously dead or sedated; tox screen pending |  |  |  |  |  |
|  |  |  | min: 20:23-20:40 | Toxicological findings showed anesthesia with a strong sleeping drug (barbiturate); then the children suffocated |  |  |  |  |  |
|  |  |  | min: 44:26 | Mrs. Reinhardt is coughing (every now and then in the course of the episode) |  |  |  |  |  |
|  |  |  | min: 1:06:03-1:06:19 | Finding of sleeping pills (Dormirol 200; active ingredient: profoxol) at Mr. Reinhardt's new wife Marijke Steen's home |  |  |  |  |  |
|  |  |  | min: 1:08:34-1:08:57 | The active ingredient in Mrs. Steen's tablets matches the one used to anesthetize the children |  |  |  |  |  |
|  |  |  | min: 1:25:45 | Mrs. Reinhardt tells that she tried to hang herself |  |  |  |  |  |

| *Tatort* episode | First broadcast | Location | Time period | Action | Active ingredient/group of active ingredients | Mechanism of action/symptoms | Literature | Plausibility/Detailedness | Time comparison |
| --- | --- | --- | --- | --- | --- | --- | --- | --- | --- |
| *Sleeping Dogs (765) (1)* | 30.05.2010 | Bremen | min: 02:46-03:35 | Discovery of the body of Ruth Thalheim; for the time being, no evidence of third-party involvement | **Zytrex 3:** fictitious | No mechanism because fictitious | No literature because fictitious; **(1)** Baxmeyer (2010) | **Symptoms:** edema due to puncture, ventricular fibrillation - fictitious; **Mechanism of action:** no; **Substance explanation:** poison from Stasi times, no antidote - fictitious; **Latency:** 6-10h - fictitious; **Dose:** no; **Cause of death:** cardiac arrest - fictitious; **Route of administration:** injected - fictitious; **P:** cannot be rated because fictitious; **D:** 2 | no |
|  |  |  | min: 05:08-05:20 | The forensic pathologist suspects a heart attack |  |  |  |  |  |
|  |  |  | min: 23:33-24:55 | The forensic pathologist suspects a puncture site at the knee because of an edema, but he does not find a matching hematoma; tox screen was negative; Inspector Inga Lürsen suspects a poison from Eastern Germany times, as these are difficult to detect; "The poisons are not detectable with the standard screening" |  |  |  |  |  |
|  |  |  | min: 39:01-39:42 | The forensic pathologist‘s report showed that poisoning was done with Zytrex 3; this is a synthetic poison developed by the Eastern German State Security Stasi; cause of death is cardiac failure (first ventricular fibrillation, then cardiac arrest and then death), some hours after administration; administration of the poison: injected via an ultrafine needle or via the mucosa; no antidote known; the poison is not accessible but if old recipes are available, it can still be produced |  |  |  |  |  |
|  |  |  | min: 1:12:25-1:12:38 | The injection was recorded on video; this was 6-10 hours before death |  |  |  |  |  |
|  |  |  | min: 1:17:33-1:17:42 | Zytrex 3 is found in a refrigerator |  |  |  |  |  |
|  |  |  | min: 1:19:10-1:19:56 | Mr. Schröder was also poisoned; he is very short of breath, then becomes unconscious and dies |  |  |  |  |  |

| *Tatort* episode | First broadcast | Location | Time period | Action | Active ingredient/group of active ingredients | Mechanism of action/symptoms | Literature | Plausibility/Detailedness | Time comparison |
| --- | --- | --- | --- | --- | --- | --- | --- | --- | --- |
| *Legacy sites (750) (5)* | 27.12.2009 | Stuttgart | min: 02:23-02:57 | A mortician notices unusual marks on the corpse of Mr. Schubert: "Burn mark from a foam fungus (...) this could well be due to poisoning"; in the documents, the dilated pupils are not mentioned; an autopsy is ordered | **Barbiturates:** belong to the sedatives, hypnotics and injection narcotics (1); methohexital and thiopental are used as injection narcotics and phenobarbital is used for its anticonvulsant effect (1); **benzodiazepines:** frequently used drug class (1); **methaqualone (2-methyl-3-o-tolyl-4 (3H)-quinazolinone):** belongs to sedatives and hypnotics (2); effect and chemical structure similar to barbiturates (2); produced in 1973 in search of antimalarial drugs (2); misused as a recreational drug due to euphoric effect (2); **diphenhydramine:** First-generation H1 receptor antagonist of (1); used for type 1 allergies, insect bites, sunburns, insomnia, vomiting during pregnancy, kinetosis, and for premedication before anesthesia (1); **etodoxizine:** fictitious | **Barbiturates:** rapidly cross BBB and rapid onset of action (1); binding to the GABAA receptor and enhanced inhibition of excitatory neurotransmission and strengthening of GABAergic inhibitory transmission (1); unlike benzodiazepines, they can also cause activation of the receptor in high doses without the presence of GABA (1); sedative-hypnotic, antiepileptic but not analgesic, anxiolytic, muscle relaxant (1); much easier to overdose (1); contraindications e.g. alcohol (intoxication), sleeping pills or analgesics (1); **benzodiazepines:** binding to benzodiazepine binding site at GABAA receptor (1);enhancement of GABAergic inhibition (1); effect of self-limitation-> weak GABA responses of the cell are enhanced but strong ones not beyond physiological maximum ->. therefore not lethal alone but only in combination with other sedatives -> difference from barbiturates (1); anxiolytic, sedative-hypnotic, muscle relaxant, and antiepileptic (1); **methaqualone:** Positive allosteric GABAA receptor modulator (2); no binding to the binding sites of barbiturates, benzodiazepines, or neurosteroids (2); **diphenhydramine:** causes sedation, has antiallergic effects, and causes itch relief (1); ADR in combination with alcohol: severe sedation (1); overdose: antimuscarinic syndrome (1) | **(1)** Seifert (2019), pp. 95, 310ff, 328; **(2)** [Ionescu-Pioggia](https://doi.org/10.1097/00004850-198804000-00001) et al. (1988); **(3)** Hufschmidt, Lückring (2009); **(4)** Hell, Böker (2005); **(5)** Moore (2009) | **Symptoms:** Mydriasis - implausible since barbiturates and benzodiazepines tend to cause miosis (3); **mechanism of action:** no; **substance explanation:** sedatives and sleeping pills; **latency:** dose dependent - plausible; **dose:** no; **cause of death:** no; **route of administration:** oral - plausible; no separation of the three substances from benzodiazepines and barbiturates - misunderstanding about affiliation; plausible scenario, since the number of suicides (partly unnoticed) due to e. g. tablet overdoses, among seniors or sick people is high (4); **P:** 4; **D:** 6 | Comparison with "Sons and Fathers,"  "The Legal Case Reinhardt Case" and "crooked dogs " with regard to barbiturate presentation |
|  |  |  | min: 03:20-03:40 | Forensic pathologist confirms cause of death: poisoning; toxicological examination still pending; suspects barbiturates or benzodiazepines -> statement that tranquilizers or an overdose of sleeping pills were the cause of death |  |  |  |  |  |
|  |  |  | min: 11:47-12:26 | Mr. Schubert's physician said he did not think it was unusual for him to have dilated pupils because he was in severe pain from osteoporosis; When found, Mr. Schubert was in bed and there was probably no evidence of third-party involvement |  |  |  |  |  |
|  |  |  | min: 21:20-21:55 | The following drugs were found at the Schubert's home: methaqualone, diphenhydramine and etodoxizine; these are sleeping pills. After overdose, time until death varies |  |  |  |  |  |
|  |  |  | min: 38:10-38:22 | Statement that the sleeping pills were administered with blueberry compote and whipped cream |  |  |  |  |  |
|  |  |  | min: 45:18-47:30 | Mrs. Schubert swallows a large amount of pills and lies down; when Inspector Lannert finds her, she is unconscious and he calls the ambulance; at the hospital, Mrs. Schubert is already as fit as before. |  |  |  |  |  |
|  |  |  | min: 1:18:22-1:20:05 | Granddaughter Leonie explains that she was at her grandparents in the evening and her grandfather was already asleep. |  |  |  |  |  |
|  |  |  | min: 1:21:42-1:23:38 | Playing the farewell video of the Schubert couple -> it was unsuccessful double suicide |  |  |  |  |  |
|  |  |  | min: 1:24:42-1:25:47 | Mr. Schubert eats the blueberry compote and before his wife eats the compote, granddaughter Leonie comes in |  |  |  |  |  |

| *Tatort* episode | First broadcast | Location | Time period | Action | Active ingredient/group of active ingredients | Mechanism of action/symptoms | Literature | Plausibility/Detailedness | Time comparison |
| --- | --- | --- | --- | --- | --- | --- | --- | --- | --- |
| *Burn mark (708) (1)* | 19.10.2008 | Cologne | min: 02:34-02:53 | There is one fatality (a young woman) in a house fire; the victim was asleep when the fire started | see episode 1018 | see episode 1018 | see episode 1018; **(1)** Pfeiffer (2008) | **Symptoms:** Unconsciousness - plausible; **Mechanism of action:** no; **Substance explanation:** no; **Latency:** Two breaths lead to unconsciousness - imprecise and dependent on CO concentration; **Dose:** CO-Hb 61%; **Cause of death:** carbon monoxide poisoning - plausible; **Route of administration:** inhalation - plausible; Unconsciousness during sleep - plausible; **P:** 1; **D:** 5 | Comparison with "In the End You Go  Naked, "The Legal Case Reinhardt" “Fallen Angels" and "Eight Years Later" with regard to carbon monoxide poisoning |
|  |  |  | min: 03:20.03:37 | Cause of death is carbon monoxide poisoning; "After 2 breaths you lose consciousness"; the fire started in the basement, and probably an accelerant was used |  |  |  |  |  |
|  |  |  | min: 07:07-07:11 | Report that the victim died on the way to the hospital |  |  |  |  |  |
|  |  |  | min: 09:44-09:57 | Forensic pathologist’s report: "CO-Hb 61%" -> Carbon monoxide in lethal dose |  |  |  |  |  |

| *Tatort* episode | First broadcast | Location | Time period | Action | Active ingredient/group of active ingredients | Mechanism of action/symptoms | Literature | Plausibility/Detailedness | Time comparison |
| --- | --- | --- | --- | --- | --- | --- | --- | --- | --- |
| *Crooked dogs (699) (2)* | 18.05.2008 | Münster | min: 57:20-57:35 | Mr. Wesskamp is found dead in the summerhouse; Cause of death: barbiturates in combination with alcohol; forensic pathologist Boerne: "The ticket to the hereafter” | **Barbiturates:** included in sedatives, hypnotics, and injectable narcotics (1); methohexital and thiopental used as injectable narcotics and phenobarbital used for anticonvulsant effects (1); **thelotal:** fictitious barbiturate | **Barbiturates:** easily cross the BBB, rapid onset of action (1); binding to the GABAA receptor, enhanced inhibition of excitatory neurotransmission, enhanced GABAergic inhibitory transmission (1); Unlike benzodiazepines, barbiturates in high doses can also cause activation of the receptor without GABA being present (1); sedative-hypnotic, antiepileptic but not analgesic, anxiolytic, muscle relaxant (1); much easier to overdose (1); contraindications e.g. alcohol (intoxication), sleeping pills or painkillers (1) | **(1)** Seifert (2019), pp. 310ff, p. 328; **(2)** Stelzer (2008) | **Symptoms:** no; **Mechanism of action:** no; **Substance explanation:** disgusting taste of thelotal - fictitious; Combination of barbiturate and alcohol is lethal - plausible; **Latency:** no; **Dose:** 30 tablets of 35mg each - unclear if all tablets were taken; therefore too imprecise; **Cause of death:** no; **Route of administration:** oral - plausible; **P:** 2; **D:** 6 | Comparison with "Sons and Fathers," "The Legal Case Reinhardt" and "Old Burdens" with regard to barbiturate presentation |
|  |  |  | min: 58:57-59:41 | Impossible to take the medication unknowingly, because thelotal tastes "disgusting" and you taste it whatever you eat; the package contains 30 tablets with 35mg active ingredient each -> "enough for half a herd of elephants"; in 2002 the barbiturate has already expired |  |  |  |  |  |
|  |  |  | min: 1:01:10-1:01:25 | Statement of Prof. Breitmeyer (pharmacologist): Thelotal remains effective even after the expiration date. |  |  |  |  |  |
|  |  |  | min: 1:01:45-1:02:18 | In 2002, the composition of thelotal was modified -> the bitter taste was added, because there had been a case of poisoning before |  |  |  |  |  |
|  |  |  | min: 1:02:35-1:02:40 | The old case was an inheritance dispute in Flensburg in 1998 |  |  |  |  |  |
|  |  |  | min: 1:25:45-1:25:52 | Statement that murders by poisoning are mainly carried out by women |  |  |  |  |  |

| *Tatort* episode | First broadcast | Location | Time period | Action | Active ingredient/group of active ingredients | Mechanism of action/symptoms | Literature | Plausibility/Detailedness | Time comparison |
| --- | --- | --- | --- | --- | --- | --- | --- | --- | --- |
| *Shadow Games (637) (5)* | 20.08.2006 | Hamburg | min: 04:27-06:27 | The pre-removal detainee Jonathan Waputo vomits profusely; is in severe pain; is picked up by paramedics; is shaking badly and sweating; he nevertheless manages to pull a guard's gun and escapes; because of the discomfort he can only walk hunched over | **Aconitine:** Poison from Aconitum napellus (blue monkshood) (1); the plant is considered the most poisonous plant found here (1); ingredients used for murders by poisoning in ancient times (1); grown as an ornamental plant (1); the diterpene alkaloid aconitine is one of the many ingredients (1); is found in tubers and seeds, in flowers and leaves; the concentration depends on the season (2); aconitine is also found in other plants belonging to the genus "Aconitum" (2); lethal dose: 3-6 mg or 2-15 g aconite root (1); former use as a homeopathic medicine, then banned because of intoxications (1); nowadays partly misused as a stimulant, with sometimes fatal consequences (1); also used in Traditional Chinese Medicine, where it is hydrolyzed to purer or nontoxic derivatives (3); accidental poisonings rare, due to the pungent taste (1); absorption via mucous membranes (1); latency at high doses: a few minutes (1) | **Aconitine:** belongs to the class of Na+ channel agonists (3); directly activates or inhibits closing of voltage-gated sodium channels (3); results in prolonged sodium influx which initially increases excitability (3); but later reverses and paralysis occurs (4); cardiotoxic and neurotoxic (3); also hypotensive and bradycardic effects as it activates the ventromedial nucleus of the hypothalamus (3); At onset: "sensory disturbances such as tingling, burning, numbness in the mouth and limbs" (2); spread to entire body (2); nausea with vomiting and diarrhea also possible (2); severe pain in various regions (2); cause of death: respiratory paralysis and cardiac arrhythmias or even ventricular fibrillation (2); therapy: toxin elimination and inhibition of further absorption (1); no antidote (1) | **(1)** Dekant, Vamvakas (2010), pp. 247f; **(2)** Aktories et al. (2013), p. 1070; **(3)** Chan (2009); **(4)** Arnold (2020); **(5)** Garde (2006) | **Symptoms:** vomiting, severe pain, trembling, sweating, collapse- plausible; **mechanism of action:** no; **explanation of substance:** poisonous alkaloid from aconite - plausible; still used today as rheumatism remedy - not described in literature; poison in root - plausible; purchase of plant possible - plausible as it is an ornamental plant in Europe; **latency period:** no; **dose:** no; **cause of death:** respiratory arrest - plausible; **route of administration:** oral - plausible; strength to hold gun and walk around - plausible due to central excitation; paralysis after central excitation - absent; improvement: first collapse due to paralysis, then respiratory distress and then death; cardiotoxicity - absent; **P:** 2; **D:** 2 | no |
|  |  |  | min: 06:46-07:37 | Mr. Waputo collapses dead; he was previously taken away by ambulance with suspected ruptured appendix |  |  |  |  |  |
|  |  |  | min: 08:47-09:12 | Forensic pathologist’s report: cause of death was respiratory arrest due to acute poisoning; toxicological examination still pending |  |  |  |  |  |
|  |  |  | min: 16:14-16:28 | Finding of food leftovers and tablets of Mr. Waputo -> could be possible route of administration |  |  |  |  |  |
|  |  |  | min: 16:49-17:15 | A person who is doing the alternative civilian service in the prison explains that all prisoners get vitamins and vaccinations; blood is taken because of the illicit drugs; Waputo had only taken his vitamins |  |  |  |  |  |
|  |  |  | min: 20:26-20:46 | The pathology has identified the poison: aconitine -> "an extremely toxic alkaloid extracted from a plant called aconite. However, it is also used pharmaceutically, e.g. in antirheumatic drugs"; he had probably been given 4 different medicines recently |  |  |  |  |  |
|  |  |  | min: 21:25-21:33 | Three vitamin preparations and one oral typhoid vaccination were administered. |  |  |  |  |  |
|  |  |  | min: 25:16 | Another inmate eats the cake which Mr. Waputo had eaten before he died and which is suspected to be poisoned |  |  |  |  |  |
|  |  |  | min: 42:20-42:31 | The cake was not poisoned, because he had already eaten it at noon and had survived |  |  |  |  |  |
|  |  |  | min: 46:07-46:19 | The tablets, which were supposed to be vitamin B, are anti-rheumatic drugs which are not yet approved. The active ingredient is aconitine -> illegal drug testing; aconitine was found in all blood samples taken |  |  |  |  |  |
|  |  |  | min: 48:33-48:48 | The aconitine content in the analgesic should be one hundred times less than the lethal dose |  |  |  |  |  |
|  |  |  | min: 52:23-52:31 | Statement that Waputo did not die from the drugs; the blood samples of the other prisoners showed a different degree of purity of aconitine than he was poisoned with |  |  |  |  |  |
|  |  |  | min: 53:17-53:23 | Presumption that the poison was obtained from the root of the blue monkshood, as traces of soil were found in the stomach of the victim |  |  |  |  |  |
|  |  |  | min: 1:07:10-1:07:13 | No monkshood has been sold in Hamburg in the last weeks |  |  |  |  |  |

| *Tatort* episode | First broadcast | Location | Time period | Action | Active ingredient/group of active ingredients | Mechanism of action/symptoms | Literature | Plausibility/Detailedness | Time comparison |
| --- | --- | --- | --- | --- | --- | --- | --- | --- | --- |
| *Sun and Storm (545) (1)* | 02.11.2003 | Hanover | min: 04:43.04:50 | Inspector Charlotte Lindholm receives a letter with a protein bar inside | **Trimethanoctulol phenyl carbon hydrite:** fictitious | No mechanism because fictitious | No literature because fictitious; **(1)** Jauch (2003) | **Symptoms:** Nausea, pain, cough, shortness of breath- fictitious; **Mechanism of action:** no; **Substance explanation:** poison from anti-corrosion agent, also used in mechanical engineering, shipping industry - fictitious; **Latent period:** no; **Dose:** no; **Cause of death:** respiratory paralysis - fictitious; **Route of administration:** oral- fictitious; **P:** cannot be rated because fictitious; **D:** 3 | no |
|  |  |  | min: 05:21-06:16 | Investigations reveal that the bar has been poisoned; "The poison originates from an anti-corrosion agent. Mechanical engineering, shipping industry. The effect can be very impressive."; The poison is called trimethanoctulol phenyl carbon hydrite |  |  |  |  |  |
|  |  |  | min: 07:49-08:04 | Mr. Surdrup coughs and has nausea |  |  |  |  |  |
|  |  |  | min: 08:50-09:22 | Mr. Surdrup is lying on the floor and has severe pain (stomach cramps); drinking seems to increase the pain |  |  |  |  |  |
|  |  |  | min: 10:18-10:32 | Mr. Surdrup is examined and the ambulance service is called because of severe poisoning |  |  |  |  |  |
|  |  |  | min: 17:56-19:17 | Mr. Surdrup is in the hospital; he is oriented and lucid; but is still coughing and having a little pain |  |  |  |  |  |
|  |  |  | min: 21:31-21:44 | The poison is injected into the bar with a syringe |  |  |  |  |  |
|  |  |  | min: 40:07-40:14 | In the stomach contents, the protein bar and traces of poison have been found |  |  |  |  |  |
|  |  |  | min: 43:25-44:00 | The anti-corrosion agent and a syringe are found |  |  |  |  |  |
|  |  |  | min: 46:43-46:50 | Mr. Surdrup still has a severe cough |  |  |  |  |  |
|  |  |  | min: 52:47-53:25 | The inspector says that Mr. Surdrup looks better, but the doctor says just the opposite: "The poison has already penetrated deep into the cells"; The doctor explains that Mr. Surdrup will die: "It starts with cough attacks, then at some point the respiratory paralysis begins. With his asthma, the deterioration is particularly rapid." |  |  |  |  |  |
|  |  |  | min: 54:30-55:18 | Mr. Surdrup suffers from shortness of breath |  |  |  |  |  |
|  |  |  | min: 1:03:29-1:04:20 | Mr. Surdrup passes away |  |  |  |  |  |

| *Tatort* episode | First broadcast | Location | Time period | Action | Active ingredient/group of active ingredients | Mechanism of action/symptoms | Literature | Plausibility/Detailedness | Time comparison |
| --- | --- | --- | --- | --- | --- | --- | --- | --- | --- |
| *When Women Eat Oysters (542) (2)* | 12.10.2003 | Munich | min: 06:36-07:12 | After eating oysters, Anna Stahlberg-Zeulig does not feel well and is taken to a room | **Synthetic poisons:** variety of different poisons that do not occur naturally but are produced artificially by humans (1); they can be produced directly or are created as by-products in the production process of other products (1) | No mechanism because no exact substance is mentioned | **(1)** Bahnsen (2016); **(2)** Emmerich (2003) | **Symptoms:** shortness of breath, unconsciousness- unclear which substance; **mechanism of action:** no; **substance explanation:** no; **latency:** no; **dose:** no; **cause of death:** no; **route of administration:** oral- possible; **P:** cannot be rated because of unknown substance; **D:** 6 | no |
|  |  |  | min: 07:50-08:09 | Mrs. Stahlberg-Zeulig lies in bed and breathes heavily; tries in vain to reach a telephone |  |  |  |  |  |
|  |  |  | min: 10:13-10:33 | Ms. Stahlberg-Zeulig's breathing is shallow and rapid |  |  |  |  |  |
|  |  |  | min: 12:13-12:21 | Mrs. Stahlberg-Zeulig lies unconscious on the floor |  |  |  |  |  |
|  |  |  | min: 13:45-14:00 | Mrs. Stahlberg-Zeulig's death is ascertained by the police |  |  |  |  |  |
|  |  |  | min: 14:16 | Mrs. Stahlberg-Zeulig had called the police before she died. She said that she had been poisoned |  |  |  |  |  |
|  |  |  | min: 19:00-19:12 | Pathology confirms that the victim was really poisoned; it was a synthetic poison; traces of poison were found on an oyster |  |  |  |  |  |

| *Tatort* episode | First broadcast | Location | Time period | Action | Active ingredient/group of active ingredients | Mechanism of action/symptoms | Literature | Plausibility/Detailedness | Time comparison |
| --- | --- | --- | --- | --- | --- | --- | --- | --- | --- |
| *Bienzle and the Sweet Death (505) (4)* | 14.07.2002 | Stuttgart | min: 09:18-09:26 | Eight-year-old Sascha expresses nausea and a twinge in the abdomen | **Digitalis:** Term for the plant genus of the Plantaginaceae family (also plantain family) (1); the active ingredients digitoxin (from Digitalis purpurea) and digoxin (from Digitalis lanata) contained therein belong to the class of the cardiac glycosides (1); both are the only cardiac glycosides in clinical use today (1); the term cardiac glycoside is outdated and should no longer be used (1); the more correct term is Na+/K+-ATPase inhibitor (1); **exhaust gases:** exact gas unknown; often carbon monoxide (CO), carbon dioxide (CO2), and/or oxides of nitrogen (NOx) (2) | **Digitalis:** Inhibition of Na+/K+-ATPase (3); increase of intracellular calcium concentration (3); results in a positive inotropic effect on the heart, as well as negative dromotropic effect (by stimulation of parasympathetic nervous system) (3); intoxication: characteristic yellow-green vision (3); various cardiac arrhythmias (up to cardiac arrest), nausea and vomiting, CNS dysfunctions (3); disorders of electrolyte metabolism with hypo- or hyperkalemia are possible (3) | **(1)** Aktories et al. (2013), p. 414; **(2)** Ziegler et al. (2014); **(3)** Seifert (2019), p. 212; **(4)** Agthe (2002) | **Symptoms:** Nausea, vomiting, abdominal pain, unconsciousness, yellow-green vision, potassium drop - plausible; **mechanism of action:** no; **substance explanation:** no; **latency:** no; **dose:** dose-dependent lethality - plausible; **cause of death:** cardiac arrest - plausible; **route of administration:** oral - plausible; possible suffocation by exhaust - plausible; Bienzle’s unconsciousness followed by immediate recovery without oxygen administration - implausible; **P:** 2; **D:** 2 | no |
|  |  |  | min: 10:22-11:22 | Sascha is very sweaty; he vomits quite a lot and several times; then he collapses and is unconscious; the emergency service is called |  |  |  |  |  |
|  |  |  | min: 14:30-14:38 | Sascha is in the emergency room and is being cared for; no gastric lavage is done but the vomit is sucked out |  |  |  |  |  |
|  |  |  | min: 15:16-15:35 | The doctor suspects a severe poisoning; it is not yet clear which poison it is but probably it must have been quite a high dose |  |  |  |  |  |
|  |  |  | min: 16:37-16:51 | Sascha has passed away |  |  |  |  |  |
|  |  |  | min: 18:38-18:42 | The doctor believes that it is a poison that acts on the heart |  |  |  |  |  |
|  |  |  | min: 31:30-31:37 | Before Sascha died he had said to his aunt that she looked yellow/green |  |  |  |  |  |
|  |  |  | min: 34:43-35:43 | The forensic pathologist suggests that the cause of death was cardiac paralysis; yellow/green vision is very typical of digitalis poisoning; "Everything fits perfectly together: the rapid vomiting, the drastic drop in potassium levels and the fact that the child lost consciousness so quickly. Digitalis antidote should have been given"; lethality depends on dose |  |  |  |  |  |
|  |  |  | min: 54:10-54:46 | Liquefied digitalis was detected in the pralines of Sascha's family; the pathologist believes that the tablets were crushed or ground in a mortar, then dissolved in water and finally injected into the pralines with a syringe |  |  |  |  |  |
|  |  |  | min: 59:58-1:00:35 | A syringe is used to draw up liquefied digitalis and inject it into pralines; then the boxes in the supermarket are exchanged with the poisoned boxes |  |  |  |  |  |
|  |  |  | min: 1:00:36-1:01:09 | A young girl is hospitalized with poisoning: the circulation is to be stabilized, electrolytes are to be given and an ECG has to be made; she has also eaten pralines |  |  |  |  |  |
|  |  |  | min: 1:12:57 | A man places a compressor in front of the unconscious Inspector Bienzle and wants him to die from the exhaust fumes |  |  |  |  |  |
|  |  |  | min: 1:13:31-1:13:34 | The door of the shed in which Bienzle is lying is locked; Sascha's father warns: "But he's suffocating. |  |  |  |  |  |
|  |  |  | min: 1:15:53-1:16:05 | Bienzle's colleague finds him and helps him escape |  |  |  |  |  |

| *Tatort* episode | First broadcast | Location | Time period | Action | Active ingredient/group of active ingredients | Mechanism of action/symptoms | Literature | Plausibility/Detailedness | Time comparison |
| --- | --- | --- | --- | --- | --- | --- | --- | --- | --- |
| *Quartet in Leipzig (458) (3)* | 26.11.2000 | Dresden/ Leipzig | min: 04:49-05:12 | A newspaper article reports on a death in an IC train: "Cardiac death"; Inspector Ballauf is called by his forensic pathologist who reports: "The man died from a high dose of botulinum toxin. A substance that is only used in clinical medicine." | **Botulinum toxin:** colloquial term Botox (1); one of the best known strongest toxins (1); dose of less than one microgram can be lethal (1); formed by bacterium Clostridium botulinum (1); occur everywhere and belong to the gas-forming rod bacteria (1); anaerobic bacteria- > grow and produce best in the absence of air and in a protein-containing environment (1); occur in spoiled food (e.g. canned food) (1); heat-labile toxin is destroyed by cooking the food (long and hot enough); recent studies report use in patients with depression (2) | **Botulinum toxin:** inhibition of acetylcholine release from presynaptic cells (1); latency period after consumption of contaminated food: 12 hours to 2 days (1); symptoms: "dry mouth, impaired vision, speech and swallowing, ptosis of eyelids, muscle weakness in neck and extremities" (1); high intoxication: death from cardiac arrest or respiratory paralysis after 2-10 days (1); according to study reports, there have been antidepressant effects after injection into the "anger muscles" such as procerus muscle (2) | **(1)** Dekant, Vamvakas (2010), pp. 253f; **(2)** Finzi et al. (2018); **(3)** Heidelbach (2000) | **Symptoms:** Respiratory arrest - plausible; **Mechanism of action:** no; **Substance explanation:** i.v. use in medicine for muscle and nerve diseases - plausible; **Latency:** 2-3 minutes - implausible; **Dose:** no; **Cause of death:** cardiac arrest - plausible; **Route of administration:** oral by injection into capsules - plausible; explanation of bacteria and contaminated food - missing; **P:** 2; **D:** 4 | No comparison with “Celebration”, because poisoning was not caused by botulinum toxin in that episode. |
|  |  |  | min: 06:21-06:48 | The forensic pathologist explains: "Such a high dose is only taken voluntarily if someone wants to commit suicide. (...) In his case, breathing must have stopped after 2-3 minutes, before the heart also stopped working"; only doctors normally get hold of this substance, as the toxin is used as a drug for muscle and nerve diseases; for therapy it is injected but not taken orally; latency period is a few minutes. |  |  |  |  |  |
|  |  |  | min: 07:04-07:17 | "The toxin was injected into the capsules that the dead man had with him"; the forensic pathologist shows the inspectors the capsules that the dead man had with him and in which the toxin has now been found; one capsule is already lethal |  |  |  |  |  |
|  |  |  | min: 1:16:14-1:16:35 | Inspector Ehrlicher receives an injection from Mr. Kleist; shortly afterwards he falls asleep |  |  |  |  |  |
|  |  |  | min: 1:18:38-1:20:08 | Inspector Ehrlicher is about to receive the anesthesia; he is already dazed; inspector Schenk, however, is able to prevent the anesthesia from being administered |  |  |  |  |  |

| *Tatort* episode | First broadcast | Location | Time period | Action | Active ingredient/group of active ingredients | Mechanism of action/symptoms | Literature | Plausibility/Detailedness | Time comparison |
| --- | --- | --- | --- | --- | --- | --- | --- | --- | --- |
| *Free Rider (447) (3)* | 16.07.2000 | Cologne | min: 34:37-34:49 | The brewmaster tastes a beer and looks puzzled, as it probably tastes suspicious | **Strychnine:** main alkaloid from seeds of Strychnos nux-vomica (Brazil nut tree) (1); origin: India (1); came to Germany in the 16th century as a rodenticide (1); source of accidental poisoning because still used today partly as a pesticide (1); odorless, white powder (2) | **Strychnine:** increase of neuronal excitability by blocking inhibition (1); selective, competitive antagonist of the inhibitory neurotransmitter glycine (1); results in massive excitation, muscular convulsions (1); symptoms: first stiffness, later similar symptoms as in tetanus (opisthotonus) (1); cause of death: respiratory paralysis (1) | **(1)** Hardman et al. (2001), p. 1894; **(2)** Walensi et al. (2021); **(3)** Fischer (2000) | **Symptoms:** pain, circulatory failure - implausible; improvement: stiffness and opisthotonus; curvature of spine, foaming at mouth - plausible due to opisthotonus and dysphagia caused by convulsions; **mechanism of action:** no; **substance explanation:** powder- plausible; **latency:** no; **dose:** no; **cause of death:** no; **route of administration:** oral via capsules - plausible; screaming because of pain before arrival of ambulance, but normal behavior soon after on the ambulance stretcher - implausible; **P:** 3; **D:** 5 | no |
|  |  |  | min: 35:40-36:26 | The brewmaster walks staggering, screaming in pain, holding his stomach, sweating; on the ambulance stretcher, he speaks completely normally again and no longer screams in pain |  |  |  |  |  |
|  |  |  | min: 37:51-37:55 | The doctor says it is strychnine poisoning; he is in critical condition but will probably survive |  |  |  |  |  |
|  |  |  | min: 56:44-57:10 | A vial labeled strychnine is shown. Mr. Pigulla fills a powder into a capsule; Mr. Pigulla tells Robert Serner that he has medicine in the car |  |  |  |  |  |
|  |  |  | min: 1:00:10 | A dead body is found (Robert Serner) |  |  |  |  |  |
|  |  |  | min: 1:01:08-1:01:27 | The forensic pathologist suspects strychnine poisoning; a curvature of the spine and foaming at the mouth are typical symptoms |  |  |  |  |  |
|  |  |  | min: 1:03:45-1:03:58 | Laboratory results confirm death by strychnine poisoning; it was the same toxic composition as that used on the brewmaster; however, now the dose was lethal |  |  |  |  |  |

| *Tatort* episode | First broadcast | Location | Time period | Action | Active ingredient/group of active ingredients | Mechanism of action/symptoms | Literature | Plausibility/Detailedness | Time comparison |
| --- | --- | --- | --- | --- | --- | --- | --- | --- | --- |
| *Bitter Almonds (437) (2)* | 05.03.2000 | Cologne | min: 02:56-03:22 | According to the forensic pathologist the corpse of Gerd Weisbach smells of bitter almonds; he says that only 50% of the population can smell bitter almonds due to genetic reasons; poisoning with potassium cyanide is assumed | **Potassium cyanide (KCN):** cyanide of prussic acid (HCN) (1); fast acting (1); uses: ship and room fumigation, soil sterilization, metallurization, electroplating, insecticide, rodenticide (1); also in almonds, apricots -> odor of bitter almond (1) | **KCN:** blocking cytochrome oxidases by forming a very stable complex with an iron (III) atom of this enzyme (1); interruption of the respiratory chain and interruption of metabolic energy production -> death of the cell (1); first symptom: hyperpnea, headache, then reddish skin discoloration due to arterialization of venous blood (1); further symptoms: dizziness, convulsions (1); cause of death: respiratory paralysis (1) | **(1)** Hardman et al. (2001). P. 1893; **(2)** Heidelbach (2000) | **Symptoms:** Hyperpnea, red skin, respiratory paralysis, convulsions - plausible; **mechanism of action:** inhibition of cellular respiration - plausible; **substance explanation:** bitter- almond odor - plausible; **latency:** few minutes at high dose - plausible; **dose:** 1 gram for lethal outcome - plausible; **cause of death:** internal asphyxia - plausible; **route of administration:** oral - plausible; latency on ingestion at min. 34:29: immediate onset of symptoms - implausible; **P:** 1; **D:** 1 | Comparison with "Licorice," " Who digs a pit for others," and "Vodka Bitter Lemon" with regard to cyanide presentation |
|  |  |  | min: 04:21-05:22 | The autopsy confirms the cause of death: HCN, Blausäure-> Zyankali; "The stuff is actually harmless but in combination with an acid, e.g. stomach acid, potassium cyanide becomes hydrocyanic acid. The stuff inhibits the cellular respiratory system, the blood can no longer absorb oxygen and internal asphyxiation occurs"; At high doses, death occurs within a few minutes; "People breathe faster, first turn red, later blue, gasp for air, grab their necks, fall down like a tree, asphyxiation spasms, maybe a few more gasps, exitus"; The poison was administered orally; no evidence of external violence. |  |  |  |  |  |
|  |  |  | min: 20:23-21:00 | In Weisbach's medicine cabinet, capsules (enzyme preparations that he had to take because of the pancreatic carcinoma) were found -> according to the forensic examination, potassium cyanide was in one of these capsules; "In the full dose, exactly in 100 capsules. When we found the pillbox, there were still 38 in it. Weisbach had to take 2 capsules daily. (...) Whoever poisoned the capsules, had to wait exactly 31 days." |  |  |  |  |  |
|  |  |  | min: 22:40-23:18 | Another body in the forensic pathology; the man suffered from ALS; after diagnosis, survival is about 5 years; then death follows from respiratory failure; there is a written statement and a videotape; this is the 5th case in 3 weeks in which all persons have died in the same way -> potassium cyanide |  |  |  |  |  |
|  |  |  | min: 29:09-29:26 | All patients who died from potassium cyanide did not respond to any treatment anymore. They died at home and had the same carer (Martin Lotz) |  |  |  |  |  |
|  |  |  | min: 34:29-34:43 | On the videotape, one of the victims can be seen; after setting down the glass, he begins to gasp within a few seconds; As nothing more can be seen, inspector Ballauf switches off the tape |  |  |  |  |  |
|  |  |  | min: 51:13-51:34 | "You will receive a capsule from me. The patient should dissolve the contents in a little water and drink it. The effect starts immediately. (...) The capsule contains 1 gram, which is more than enough." |  |  |  |  |  |
|  |  |  | min: 56:18-56:32 | Discovery of the body of Martin Lotz; he also has the smell of bitter almonds; initially no traces of external violence |  |  |  |  |  |
|  |  |  | min: 56:41-57:08 | 4 capsules (vitamin preparation) are found; presumption that they contained potassium cyanide |  |  |  |  |  |
|  |  |  | min: 59:20-59:46 | The forensic pathologist explains:" The dark red, swollen stomach lining is typical of hydrocyanic acid poisoning. The dose was not very high. The man must have had strong cramps"; Because of the smaller dose, death did not occur so quickly; discovery of a gelatin capsule, which presumably contained potassium cyanide. |  |  |  |  |  |
|  |  |  | min: 1:06:00-1:06:32 | Some potassium cyanide is found (in the safe of Mr. Schlegel), which -despite tight controls - was stolen from the production facility; moreover, a notebook of the customers was found |  |  |  |  |  |

| *Tatort* episode | First broadcast | Location | Time period | Action | Active ingredient/group of active ingredients | Mechanism of action/symptoms | Literature | Plausibility/Detailedness | Time comparison |
| --- | --- | --- | --- | --- | --- | --- | --- | --- | --- |
| *Fallen Angels (397) (1)* | 20.09.1998 | Munich | min: 03:25 | Discovery of a corpse (Wolfgang Heindel) in the sewage system | see episode 1018 | see episode 1018 | see episode 1018; **(1)** Freundner (1998) | **Symptoms:** euphoria, hallucinations - plausible due to lack of oxygen. It would be better to use typical symptoms like headache, dizziness, convulsions, paralysis, unconsciousness in the context of carbon monoxide poisoning; **Mechanism of action:** no; **Substance explanation:** causes: engine, furnace, smoke from open fire - plausible; **latency:** no; **dose:** no; **cause of death:** asphyxiation - plausible; **route of administration:** inhalation - plausible; **P:** 2; **D:** 5 | Comparison with "In the End You Go  Naked, "The Legal Case Reinhardt",  "Burn mark" and "Eight Years Later" with regard to carbon monoxide poisoning |
|  |  |  | min: 09:53-11:29 | A man (Reinhardt Bode) is found in a garbage dump -> he is believed to be dead; on the way to the hospital he dies |  |  |  |  |  |
|  |  |  | min: 17:03-17:10 | Cause of death determined by the forensic pathologist: carbon monoxide poisoning; possible causes could be a gasoline engine, a stove or smoke from an open fire |  |  |  |  |  |
|  |  |  | min: 17:12-17:17 | The forensic pathologist explains to the inspectors that euphoria and hallucinations occur when there is a lack of oxygen |  |  |  |  |  |
|  |  |  | min: 24:26-25:32 | Another body is found (Mathias Lang); the forensic pathologist does not yet know the exact cause of death, but suspects asphyxiation. |  |  |  |  |  |
|  |  |  | min: 27:38-27:45 | The autopsy reveals the exact cause of death: carbon monoxide poisoning |  |  |  |  |  |
|  |  |  | min: 40:45-41:05 | The re-examination of Wolfgang Heindel's body revealed that he also died of carbon monoxide poisoning; car exhaust fumes are often used as suicide but third-party involvement is also possible |  |  |  |  |  |
|  |  |  | min: 1:15:14-1:15:27 | Discovery of the oil furnace, which was used for the exhaust gas murders |  |  |  |  |  |
|  |  |  | min: 1:21:10-1:21:14 | Confirmation that smoke from an oil stove can be used for murder |  |  |  |  |  |

| *Tatort* episode | First broadcast | Location | Time period | Action | Active ingredient/group of active ingredients | Mechanism of action/symptoms | Literature | Plausibility/Detailedness | Time comparison |
| --- | --- | --- | --- | --- | --- | --- | --- | --- | --- |
| *Death plays along (366) (1)* | 27.07.1997 | Dresden/ Leipzig | min: 38:47-39:13 | Lothar Wagner makes a sauna infusion; then he immediately gets short of breath and grabs his neck; subsequently he collapses, is unconscious and dies | **Cyanogen nitrate: fictitious** | No mechanism because fictitious | No literature because fictitious; **(1)** Vogel (1997) | **Symptoms:** shortness of breath, circulatory collapse, cough, unconsciousness - fictitious; **Mechanism of action:** no; **Substance explanation:** no; **Latency:** symptoms appear immediately - very unlikely; **Dose:** no; **Cause of death:** asphyxiation - fictitious; **Route of administration:** oral, inhalation - fictitious; **P:** cannot be rated because fictitious; **D:** 3 | no |
|  |  |  | min: 40:19-40:28 | Inspector Cain says that Lothar was killed with cyanogen nitrate; "If cyanogen nitrate is put in the infusion water, the evaporation releases it, and then death by suffocation occurs." |  |  |  |  |  |
|  |  |  | min: 1:05:03-1:05:35 | Arno drinks from his water bottle; he immediately coughs, grabs his throat, falls to the ground and has difficulty breathing; Inspector Ehrlicher sticks his finger down his throat so that he has to vomit. |  |  |  |  |  |
|  |  |  | min: 1:06:49 | Arno is taken to the hospital by ambulance |  |  |  |  |  |
|  |  |  | min: 1:07:58-1:08:08 | "Cyanogen nitrate, probably injected. Here you can see the injection site at the neck of the bottle." |  |  |  |  |  |
|  |  |  | min: 1:09:15-1:10:05 | Discovery of a syringe; the analysis confirmed that it contained cyanogen nitrate |  |  |  |  |  |
|  |  |  | min: 1:11:19-1:13:06 | Arno is in the hospital and can speak normally again; he admits that he put the poison in Lothar's infusion bottle, but he did not want to kill him with it. |  |  |  |  |  |
|  |  |  | min: 1:18:50-1:19:40 | Andrea says that she killed her butterflies, which had hung in her apartment, with cyanogen nitrate; thereupon she shows the inspectors a suitcase in which she keeps everything, but the poison is missing |  |  |  |  |  |
|  |  |  | min: 1:24:51-1:24:58 | Karin tries to poison Mike with a syringe of cyanogen nitrate, but does not succeed |  |  |  |  |  |
|  |  |  | min: 1:26:33-1:27:01 | Karin stabs herself in the upper part of her body and immediately falls unconscious; she dies and is taken away |  |  |  |  |  |

| *Tatort* episode | First broadcast | Location | Time period | Action | Active ingredient/group of active ingredients | Mechanism of action/symptoms | Literature | Plausibility/Detailedness | Time comparison |
| --- | --- | --- | --- | --- | --- | --- | --- | --- | --- |
| *Deadly Gallop (364) (9)* | 29.06.1997 | Dresden/ Leipzig | min: 07:40 | Discovery of the body of Agnes Demrau | **Kilat:** fictitious; **Pervitin:** analeptic amine (1); 1-phenyl-2-methylaminopropane was developed in 1934 (1); similarity to adrenaline and ephedrine (1); dose-dependent onset of action (1); twice as effective as the similar benzedrine (1); used as a drug with the active ingredient methamphetamine 1938-1988 (2); widely used during World War II (2); still known today as e. g. “crytal meth”; **Valocordin:** drug with two possible active ingredients: doxylamine (a first-generation H1 receptor antagonist) or diazepam (a benzodiazepine) (3,4); used for sleep disorders (3); **Tilur:** drug with active ingredient acemetacin (5); COX inhibitor (6); indication: osteoarthritis, ankylosing spondylitis or inflammatory rheumatic diseases (6) | **Pervitin:** influences mental and physical components (1); increase in performance (1); effect on circulatory system from dose of 9-15 mg (1); from a certain dose increase in blood pressure -> cannot be explained by "constriction of renal circulation", since renal perfusion is increased by Pervitin (1); bronchodilation and decongestion of the mucous membranes of the respiratory tract (1); relaxation of the stomach muscles (1); reduction of appetite (1); performance enhancement is accompanied with a feeling of strength and self-confidence (2); reduction of pain sensation (2); psychosis and paranoia are possible (2); **Valocordin:** doxylamine: central depressant effect (7); diazepam: "anxiolytic, sedative-hypnotic, muscle relaxant and antiepileptic effects" (8); **Tilur:** inhibition of prostaglandin formation (6) | **(1)** Bonhoff (2013); **(2)** Pervitin (w.d.); **(3)** Gmbh (2022); **(4)** Editorial Gelbe Liste Pharmindex (2022); **(5)** Gmbh (w.d.); **(6)** Editorial Gelbe Liste Pharmindex (2016); **(7)** Kahle (2020); **(8)** Seifert (2019), p. 312; **(9)** Panzer (1997) | **Symptoms:** no; **mechanism of action:** Pervitin: excitatory effect on CNS - plausible; **substance explanation:** Kilat from southeast Asia, south Borneo, not an alkaloid - fictitious; Pervitin: adrenaline related - plausible; Valocordin: taken for nervous heart conditions - implausible; **latency:** no; **dose:** no; **cause of death:** no; **route of administration:** Pervitin: on sugar cube for a horse - plausible; statement at min: 1:26:14 (bottle with black label which should contain poison) - too inaccurate because it was not explicitly said before that it contained poison (it should be Tilur); **P:** without Kilat 5; **D:** 6 | Comparison with "Sons and Fathers," "Murder is the Best Medicine," "Legacy" in terms of  benzodiazepine depiction. |
|  |  |  | min: 12:07-12:18 | The forensic pathologist’s first hypothesis is poisoning; "I've never seen anything like that. As if she fell asleep while walking. From one second to another." It is still unclear which poison was causative. |  |  |  |  |  |
|  |  |  | min: 25:06-25:48 | The poison probably originates from South-East Asia-> more precisely from South Borneo; it is called Kilat (lightning); "I don't know if Kilat can be synthesized yet like the South American Curare, so it would be relatively easy to get. It is not an alkaloid either." |  |  |  |  |  |
|  |  |  | min: 25:54-26:14 | The sugar which Agnes gave her racehorse before the race contained an alertness aid, a stimulant with a strong excitatory effect on the central nervous system. “Pervitin is certainly familiar to you. Chemically it is related to adrenaline and here we go." |  |  |  |  |  |
|  |  |  | min: 40:58-41:45 | Mr. Kasunke explains that Pervitin is a stimulant or can be used for doping; he shows inspector Ehrlicher his small medicine cabinet, which also contains Pervitin. |  |  |  |  |  |
|  |  |  | min: 1:08:19-1:09:09 | Mrs. Brekelsen takes Valocordin out of her bag; she explains that she occasionally has "nervous heart complaints"; since the bottle is half empty, inspector Ehrlicher wants to have it examined in the laboratory; then she takes out a second bottle with a black label which also says Valocordin; then she says that the first bottle really contains only Valocordin. The second, full bottle contains Tilur, not Kilat. |  |  |  |  |  |
|  |  |  | min: 1:26:14-1:26:26 | Inspector Ehrlicher tells Mrs. Brekelsen that a half-full bottle of Valocordin with a black label was found in Mr. Högen's pocket |  |  |  |  |  |

| *Tatort* episode | First broadcast | Location | Time period | Action | Active ingredient/group of active ingredients | Mechanism of action/symptoms | Literature | Plausibility/Detailedness | Time comparison |
| --- | --- | --- | --- | --- | --- | --- | --- | --- | --- |
| *Bienzle and the Dream of Happiness (342) (5)* | 29.09.1996 | Stuttgart | min: 20:43-21:17 | An English worker (Edward) of a quarry coughs repeatedly | **Asbestos:** generic term for fibrous, naturally occurring minerals (2); subdivided into serpentine asbestos (curved fibres) and amphibole asbestos (needle-like structure) (1); highly resistant to mechanical stress, acids and heat (2); used in industry, construction work and engineering (2); preferred use of the dangerous blue asbestos (crocidolite (amphibole asbestos)) and white asbestos (chrysotile (serpentine asbestos)) (2); in general use since 1900; in Germany, increased use after World War II (2); maximum use was in 1980; thereafter the use decreased (2); in 1990, classification as carcinogenic and thus largely banned (2); asbestos-related diseases are often caused by the profession -> affects "roofers, bricklayers and facade builders, (...) civil engineering workers, (..) motor vehicle mechanics, (...) welders and foundry workers, (...) heating installers (...) insulators and power plant workers" (2); latency period of fibrogenic effect: 15-20 years, of carcinogenic effect: 25-40 years (1); **dibenzodioxin:** polychlorinated dibenzo-p-dioxin (PCDD) (3); enters the environment through numerous processes (e.(3); various mixtures with varying chlorine contents (3); chlorinated dioxins differ greatly in terms of toxicity (3); most important route of intake: consumption of fish and meat products (3); daily intake currently approx. 30 pg TEQ per person per day (3); human exposure has been declining for approx. 10 years due to reduction measures of dioxin formation (3); storage especially in adipose tissue and also in breast milk fat (3); Half-life in humans approx. 6-9 years (3); main excretion via feces (3); most potent representative: 2,3,7,8-tetrachlorodibenzodioxin (TCDD) (3) | **Asbestos:** route of exposure: inhalation of the thin fibers into the alveoli -> onward transport via lymph to the pleura and development of the carcinogenic effect (2); clinical pictures: cancers caused by asbestos: lung cancer, laryngeal cancer, malignant mesothelioma of the peritoneum, pleura, and pericardium, and pulmonary and pleural asbestosis (2); Early symptoms of laryngeal cancer: Dysphagia and hoarseness (2); prognosis better than lung cancer or mesothelioma (2); pulmonary asbestosis is fibrotic change, preferentially in lower sections (2); restrictive ventilatory disorder with significant reduction of lung expansion (2); symptoms: shortness of breath and cyanosis (2); later also cardiac symptoms due to right heart strain with hypertrophy (2); manifestation at the pleura leads only to minor restrictions of lung function and thus hardly to symptoms, except in the context of pleural effusion (2); Effect: mechanical irritation and damage of the bronchial system and lungs (1); phagocytosis of fibers by macrophages and transition to apoptosis or necrosis (1); activation of more macrophages and increase in phagocytosis (1); results in "cytokine-mediated chronic inflammatory and fibrosis response" (1); release of oxygen radicals, enhanced necrosis because of by sustained phagocytosis (1); carcinogenic effect predominantly due to "intracellular and extracellular formation of reactive oxygen species" (1); **dibenzodioxin:** varying acute toxicity (3); although there is no DNA binding and no genotoxicity, it is one of the most potent carcinogens (3); tumor-promoting effect through Ah receptor: high affinity for cytosolic receptor useful for regulating protein expression for carcinogenic hydrocarbons and other pollutants, making them available for excretion (3); toxic effects are also due to Ah-receptor interaction (3); main symptom: emaciation syndrome -> progressive weight loss (possible cause of death) (3); death within up to 8 weeks after single high-dose exposure (3); symptoms in acute poisoning: nausea/vomiting, upper respiratory tract irritation (3); with weeks of latency, chloracne appears (3); possible nerve damage, lipid metabolism disorders, liver damage (3) | **(1)** Marczynyki et al. (1999); **(2)** Seidel et al. (2007); **(3)** Aktories et al. (2013), pp. 1005f, 1055f; **(4)** Wikipedia authors (2021); **(5)** Schlotterbeck (1996) | **Symptoms:** Cough - possible because of the fibrogenic effect of asbestos, but atypical latency, or because of irritation of upper respiratory tract by dibenzodioxin; **mechanism of action:** no; **substance explanation:** both lead to severe, fatal disease after a few years - plausible; asbestos: siliceous, fibrous minerals - plausible; Seveso toxin as another name for dibenzodioxin - plausible (4); **latency period:** no; **dose:** no; **cause of death:** no; **route of administration:** inhalation; regular examination of those exposed - plausible (2); statement that all persons exposed to dibenzodioxin for a long time definitively will die – probable, but never 100% probability; **P:** 4; **D:** 5 | Comparison with "A Day Like Any Other" (1) with regard to the  dibenzodioxin presentation: (1) main route of exposure: animal food, here: waste disposal; (1) abortion as consequence, here only symptoms: cough, circulatory collapse -> in both episodes, symptoms are not discussed in detail; here with regard to asbestos, late effects are mentioned, as in (1) but not from dibenzodioxin; in both episodes, effects are missing |
|  |  |  | min: 24:22-25:16 | Evidence of illegal toxic waste disposal is destroyed -> blue barrels are dug up and taken away; all workers wear masks; Edward coughs again |  |  |  |  |  |
|  |  |  | min: 27:34-28:34 | The forensic pathologist confirms that the death of a person is caused by a blast (skull fracture and fracture of the 3rd cervical vertebra); he noticed that the victim must have recently been in contact with a lot of poison, which would have killed him in the next years |  |  |  |  |  |
|  |  |  | min: 35:57-36:09 | According to the toxicologists, the poisoning of the dead man must have occurred in the last weeks/months - when he (the victim was an English worker) was already in Germany (only half a year ago). |  |  |  |  |  |
|  |  |  | min: 39:30-39:42 | The investigators find the protective masks while searching the workers' belongings; they should not be touched without gloves |  |  |  |  |  |
|  |  |  | min: 47:38-48:36 | "Fibrous, silicate minerals, non-flammable in the test"-> thus, asbestos was detected; "Nothing more precise can be said about any further damage to health, but one thing is certain: even low concentrations lead to damage in the case of long-term exposure. Intensive examinations of those affected must follow"; in addition, the toxicologists found high doses of polychlorinated dibenzodioxins in the workers' clothing; "commonly called dioxin or Seveso poison". |  |  |  |  |  |
|  |  |  | min: 49:59-50:58 | Edward coughs again, then gets circulation problems and collapses |  |  |  |  |  |
|  |  |  | min: 51:15 | The blood pressure drops to 80 mmHg |  |  |  |  |  |
|  |  |  | min: 1:01:48-1:02:08 | The forensic pathologist tells Inspector Bienzle that all his samples show that the workers are contaminated. It all points to an illegal hazardous waste dump or something similar; anyone who is exposed to these substances for a longer period of time will die from them |  |  |  |  |  |
|  |  |  | min: 1:09:13-1:09:27 | Edward is in the hospital; he seems very weak; he does not want to tell anything about the poison |  |  |  |  |  |
|  |  |  | min: 1:12:01-1:12:50 | Edward coughs and can hardly speak |  |  |  |  |  |
|  |  |  | min: 1:14:14-1:16:00 | Edward passes away |  |  |  |  |  |

| *Tatort* episode | First broadcast | Location | Time period | Action | Active ingredient/group of active ingredients | Mechanism of action/symptoms | Literature | Plausibility/Detailedness | Time comparison |
| --- | --- | --- | --- | --- | --- | --- | --- | --- | --- |
| *An honorable house (302) (1)* | 08.01.1995 | Dresden/ Leipzig | min: 11:57-12:51 | The forensic pathologist says that in Ralf Steiner’s body no traces of illicit drug use, except for alcohol, can be detected; "The boy swallowed a heavy narcotic along with the wine"; the cause of death is drowning (in the bathtub); When he drowned he was already unconscious from the narcotic; the latency depends on the dose, in this case it amounts probably to about 40-50 minutes; the result of the drug screen is still pending | **Laposan 31:** fictitious | No mechanism because fictitious | No literature because fictitious; **(1)** Haffter (1995) | **Symptoms:** Nausea, vomiting, dizziness, unconsciousness - fictitious; **mechanism of action:** no; **substance explanation:** narcotic, sedative, used for depression; odorless, tasteless - fictitious; **latency:** 40-50 minutes - fictitious; **dose:** not lethal - inaccurate; **cause of death:** drowning - fictitious; heart attack after lethal dose - fictitious; **route of administration:** oral - fictitious; lack of information that Melanie must have taken only a small amount, as she has only mild symptoms; **P:** cannot be rated because fictitious; **D:** 2 | no |
|  |  |  | min: 50:23-51:07 | The forensic pathologist says that the narcotic is called "Laposan 31" and is quite new; it is used in hospitals; the dose was not lethal; field of application: sedation and depression; odorless and tasteless; it would cause a heart attack in lethal dose |  |  |  |  |  |
|  |  |  | min: 1:21:09-1:21:21 | Inspector Ehrlicher describes the scenario again: Mr. Steiner was to be poisoned with an overdose of an anesthetic ("Laposan 31") in a glass of wine. |  |  |  |  |  |
|  |  |  | min: 1:22:11-1:22:16 | The poison was injected with a syringe through the cork into the wine bottle |  |  |  |  |  |
|  |  |  | min: 1:22:40-1:22:46 | It is mentioned again that the dose was not lethal |  |  |  |  |  |
|  |  |  | min: 1:24:22-1:24:48 | Melanie and the victim drank the bottle of wine; then Melanie suffered from nausea, vomiting and dizziness -> clue for the killer that Steiner will not die from the dose either |  |  |  |  |  |
|  |  |  | min: 1:25:21-1:25:35 | When the murderer entered the apartment, Steiner was unconscious but not dead and therefore he was drowned in the bathtub |  |  |  |  |  |

| *Tatort* episode | First broadcast | Location | Time period | Action | Active ingredient/group of active ingredients | Mechanism of action/symptoms | Literature | Plausibility/Detailedness | Time comparison |
| --- | --- | --- | --- | --- | --- | --- | --- | --- | --- |
| *A Midsummer Night's Dream (278) (2)* | 25.07.1993 | Munich | min: 03:35-04:40 | Margot has many puncture marks on her elbow and is apparently on drugs; she is very absent and lethargic | **Heroin:** also called diacetylmorphine (1); strong agonist at opioid receptors (1); rapid development of tolerance  (1) | **Heroin:** effect via metabolites 6-monoacetylmorphine and morphine (1); are mainly smoked and snorted, in the past they were injected (1); very lipophilic and rapid CNS effect -> so-called kick -> high sensation of pleasure and extreme euphoria, followed by drowsiness and daydreaming (1); first-time use: nausea, vomiting and circulatory disturbances (1); chronic use: personality breakdown with concentration and memory disorders (1); psychotic episodes, gastrointestinal disorders, menstrual and potency disorders, skin rashes, angina pectoris and profuse sweating possible; severe overdose: miosis, convulsions, respiratory paralysis, unconsciousness and death (1) | **(1)** Dekant, Vamvakas (2010), p. 242; **(2)** Bannert (1993) | **Symptoms:** drowsiness, absence, silliness - plausible; after overdose, unconsciousness – plausible, but shortness of breath is absent; **mechanism of action:** no; **substance explanation:** no; **latency:** no; **dose:** no; **cause of death:** no; **route of administration:** injection - plausible; only the victim is confirmed to have taken heroin; the other people are only presumed to have taken heroin; **P:** 1; **D:** 6 | No comparison with "Salut Palu" with regard to the heroin presentation,  because in that episode it is unclear whether heroin was really used. |
|  |  |  | min: 11:24-11:56 | A man from a cult is injected a drug into his elbow. As a result, he becomes unconscious |  |  |  |  |  |
|  |  |  | min: 14:08-14:38 | The man deceases and the syringe lies next to his body |  |  |  |  |  |
|  |  |  | min: 15:58-16:47 | The inspector suspects that the victim was a drug addict |  |  |  |  |  |
|  |  |  | min: 30:35 | Statement that the victim died of heroin overdose |  |  |  |  |  |
|  |  |  | min: 31:40-32:48 | Several persons are heavily on drugs: are silly, sit absentmindedly on the floor; a wide variety of drugs being abused |  |  |  |  |  |
|  |  |  | min: 33:51-34:09 | Margot is again injected with a drug in the crook of her arm-> her breathing becomes calmer and she closes her eyes |  |  |  |  |  |
|  |  |  | min: 1:10:46-1:11:05 | A man called Bernd wants to overdose Margot, but does not do it |  |  |  |  |  |

| *Tatort* episode | First broadcast | Location | Time period | Action | Active ingredient/group of active ingredients | Mechanism of action/symptoms | Literature | Plausibility/Detailedness | Time comparison |
| --- | --- | --- | --- | --- | --- | --- | --- | --- | --- |
| *Salut Palu (201) (5)* | 24.01.1988 | Saarbrücken | min: 01:26-02:12 | A bag with syringes, ampoules and a tube is shown; a man draws up a still unknown substance and goes into a room where a girl/woman is lying unconscious in bed; she has numerous puncture marks in the left crook of her elbow; the man thinks she is dead and she is taken away by a boat | **Knockout drops:** imprecise term for a combination of various substances (1); often sleeping pills, tranquilizers, or party drugs (3); often overdosed (3); odorless and tasteless (3); often abused for sexual offenses or robberies (1); over 100 active ingredients are abused for this purpose (3); examples: barbiturates, benzodiazepines, antihistamines, anticholinergics, ketamine or neuroleptics, party drugs such as heroin or gamma-hydroxybutyrate (GHB) (3); heroin: also called diacetylmorphine (4); strong agonist at opioid receptors (4); rapid development of tolerance  (4) | **Knockout drops:** impairment of perception and consciousness up to amnesia (2); heroin: effect via metabolites 6-monoacetylmorphine and morphine (4); are mainly smoked and snorted, in the past, they were injected (4); very lipophilic and rapid CNS effect -> so-called "kick" -> high sensation of pleasure and extreme euphoria, followed by drowsiness and daydreaming (4); first-time use: nausea, vomiting and circulatory disturbances (4); chronic use: personality breakdown with concentration and memory disorders (4); psychotic episodes, gastrointestinal disorders, menstrual and potency disorders, skin rashes, angina pectoris and profuse sweating possible; severe overdose: miosis, convulsions, respiratory paralysis, unconsciousness and death (4) | **(1)** FNR-KO-Tropfen-Aerzteinformation.pdf (w.d.); **(2)** Verba (2007); **(3)** Wikipedia authors (2022); **(4)** Dekant, Vamvakas (2010), p. 242; **(5)** Blumenberg (1988) | **Symptoms:** unconsciousness, circulatory problems, nausea - plausible; **mechanism of action:** no; **substance explanation:** no; **latency:** no; **dose:** no; **cause of death:** no; **route of administration:** knockout drops: oral via drinks - plausible; heroin: injected - plausible, as often injected in the past; lethal substances used in the first scene are unknown – however, death is plausible, as a combination of unknown substances, in an unknown dosage is extremely dangerous; the term knock-out drops is not used - becomes clear only from action; knockout drops misused for sexual offenses - plausible; ambiguity about injected substance at min:1:12:47; Palu's condition: first unconscious and very dizzy, then immediately completely lucid - - implausible; **P:** 2; **D:** 5 | Comparison with "The King of the Gutter", "The last Oktoberfest " and "Murder is the Best Medicine " with regard to the presentation of knockout drops |
|  |  |  | min: 06:15-06:33 | inspector Max Palu shows Jacques Domberg pictures of girls who disappeared; something was put in their drinks, then they were probably taken to a fake doctor where they were given an injection and then they were taken away |  |  |  |  |  |
|  |  |  | min: 06:43-07:01 | Palu tells Domberg that his girlfriend Nina Farell is also a victim |  |  |  |  |  |
|  |  |  | min: 20:58 | A girl in a disco clinks glasses with a stranger |  |  |  |  |  |
|  |  |  | min: 21:23-21:35 | The man puts a few drops in the girl's drink and she drinks it afterwards |  |  |  |  |  |
|  |  |  | min: 22:50-23:10 | The man takes the girl outside; she expresses nausea; he wants to take her to a "doctor” |  |  |  |  |  |
|  |  |  | min: 23:56-25:01 | The man and a second man hold the girl; she is injected with something via the vein in the crook of her arm; then she falls unconscious |  |  |  |  |  |
|  |  |  | min: 30:52-31:03 | Report of dead woman in river; was dead before; cause of death likely heroin overdose; doctor found evidence of rape |  |  |  |  |  |
|  |  |  | min: 56:45-57:18 | Unconscious or dead girl is taken on a stretcher from a boat to stolen ambulance |  |  |  |  |  |
|  |  |  | min: 1:12:47 | Palu gets a shot in the right neck and is wished a good night |  |  |  |  |  |
|  |  |  | min: 1:14:36-1:15:20 | Palu first appears unconscious or dazed, then very clear |  |  |  |  |  |
|  |  |  | min: 1:18:04-1:18:22 | Nina, who now works as a prostitute, says: "They broke me". |  |  |  |  |  |

| *Tatort* episode | First broadcast | Location | Time period | Action | Active ingredient/group of active ingredients | Mechanism of action/symptoms | Literature | Plausibility/Detailedness | Time comparison |
| --- | --- | --- | --- | --- | --- | --- | --- | --- | --- |
| *The Man on the High Seat (84) (3)* | 29.01.1978 | Mainz | min: 1:04:44-1:05:33 | Enzo Turiddu staggers on deck of a motor ship; he can hardly walk; then he staggers into the wheelhouse and drives off; after that he falls down the stairs | **Exhaust gases:** exact gas unknown; often carbon monoxide (CO), carbon dioxide (CO2), and/or nitrogen oxides (NOx) (2); **carbon monoxide (CO):** tasteless, odorless gas (1); source: incomplete combustion processes of organic matter (1); automobile is largest source (1); many suicides and accidents annually (1); blood level (Hb-CO -> carboxyhemoglobin) in smokers 5.9% (1) | **CO:** complex formation with hemoglobin due to high affinity for iron (II)-containing porphyrins and hemoglobin- > blocks oxygen binding site (1); CO binding to hemoglobin also reversible, but much stronger than that of oxygen. Thus, the site is blocked and oxygen transport is minimized (1); due to high affinity (250-fold stronger), even low concentrations (approx. 500 ml/m3) in the respiratory air are sufficient to block half of the heme (1); toxic effect depends on Hb-CO level (1); symptoms due to oxygen deficiency (1); target organs: brain and myocardium (1); symptoms depend on Hb-CO level: visual impairment, mild headache, nausea, vomiting, dizziness, syncope, convulsions, coma, respiratory failure (1); death from Hb-CO concentration of 60-70% (1) | **(1)** Hardman et al. (2001), pp. 1880ff; **(2)** Ziegler et al. (2014); **(3)** Neureuther (1978) | **Symptoms:** inability to walk, hallucination, unconsciousness - if CO, then plausible because of dizziness; **mechanism of action:** no; **substance explanation:** no; **latency:** no; **dose:** no; **cause of death:** no; **route of administration:** inhalation - plausible; exhaust manipulation - plausible; lack of odor perception due to smoke from cigarettes etc. – plausible; lack of information that CO is odorless; improvement: scene in forensic medicine with brief explanations of all points; **P:** cannot be rated because no exact substance was named; **D:** 6 | Comparison with "In the End You Go Naked", "The Legal Case Reinhardt", "Burn mark", "Fallen Angels" and "Eight Years Later" with  regard to carbon monoxide poisoning is not possible, because it is not sure whether it is CO |
|  |  |  | min: 1:05:51-1:05:59 | Turiddu lies unconscious on deck |  |  |  |  |  |
|  |  |  | min: 1:07:50-1:07:55 | The body of Turiddu is taken away |  |  |  |  |  |
|  |  |  | min: 1:08:14-1:09:11 | Discovery of a second body (estate agent Helga Schumann) on the ship; probably, the engine of the ship ran in order to recharge the battery |  |  |  |  |  |
|  |  |  | min: 1:09:38-1:10:09 | In the engine room, it becomes clear that the exhaust has been manipulated; "The exhaust fumes have been piped directly into the cabin"; assumption that Turiddu did it himself (which later turns out to be wrong) |  |  |  |  |  |
|  |  |  | min: 1:10:43-1:11:11 | Presumption that the two people on board did not notice the smell of the diesel engine because they were smoking and drinking; "Besides, it always smells a bit like diesel on a barge like this. Or she thought that it was nothing unsual. But anyway, it's already giving me a headache. Apparently she was the first to die. The Italian seems to have had some kind of hallucination shortly before his death. (...) He dragged himself to the helm and gave full throttle." |  |  |  |  |  |
|  |  |  | min: 1:15:50-1:16:03 | Radio report about the deaths on the ship: the police assumes that the exhaust fumes are the cause of the deaths |  |  |  |  |  |

| *Tatort* episode | First broadcast | Location | Time period | Action | Active ingredient/group of active ingredients | Mechanism of action/symptoms | Literature | Plausibility/Detailedness | Time comparison |
| --- | --- | --- | --- | --- | --- | --- | --- | --- | --- |
| *The Girl from the House on the Opposite Side of the Street (82) (1)* | 04.12.1977 | Essen | min: 1:22:46-1:22:54 | Karl-Heinz looks at different poisons and substances | **Notin-Gamma-Corbin:** fictitious | No mechanism because fictitious | No literature because fictitious; **(1)** Gies (1977) | **Symptoms:** abdominal pain, staggering, unconsciousness - fictitious; **Mechanism of action:** no; **Substance explanation:** powder - fictitious; **Latency:** no; **Dose:** no; **Cause of death:** no; **Route of administration:** oral - fictitious; **P:** cannot be rated because fictitious; **D:** 6 | no |
|  |  |  | min: 1:25:32-1:25:48 | Karl-Heinz looks at various substances in the garage; then he takes "Notin-Gamma-Corbin", which has a "toxic" symbol |  |  |  |  |  |
|  |  |  | min: 1:28:22-1:28:29 | Karl-Heinz pours the poison (powder) into a coke |  |  |  |  |  |
|  |  |  | min: 1:29:22-1:29:52 | Karl-Heinz pours more of the poison into his coke and also pours it directly into his mouth; then he drinks the coke |  |  |  |  |  |
|  |  |  | min: 1:30:28-1:30:53 | Karl-Heinz makes some notes and then takes some more of the poison; then he runs away |  |  |  |  |  |
|  |  |  | min: 1:30:58-1:32:23 | Karl-Heinz holds his stomach and walks hunched over; then he begins to stagger; he seems to have severe abdominal pain; he curls up on the floor; then he becomes unconscious |  |  |  |  |  |
|  |  |  | min: 1:34:08-1:34:15 | Inspector Haferkamp reports that the boy is dead |  |  |  |  |  |

| *Tatort* episode | First broadcast | Location | Time period | Action | Active ingredient/group of active ingredients | Mechanism of action/symptoms | Literature | Plausibility/Detailedness | Time comparison |
| --- | --- | --- | --- | --- | --- | --- | --- | --- | --- |
| *Harm set, harm get (76) (3)* | 19.06.1977 | Saarbrücken | min: 18:50-19:05 | A man (Gollnick) has allegedly committed suicide, but his wife does not believe it; Inspector Schäfermann says: "Potassium cyanide is a fast-acting poison. There is no evidence that a visitor was in the house and urged her husband. They were with their relatives." | see episode 437 | see episode 437 | see episode 437; **(2)** Aktories et al. (2013), p. 79; **(3)** Gräwert (1977) | **Symptoms:** no; **Mechanism of action:** no; **Substance explanation:** bitter almond odor - plausible; **Latency:** acts immediately - imprecise but fast is correct; **Dose:** no; **Cause of death:** no; **Route of administration:** oral - plausible; Description of sustained release capsules - plausible (2); **P:** 2; **D:** 5 | Comparison with "Licorice", "Bitter Almonds" and  "Vodka Bitter Lemon" with regard to  potassium cyanide presentation |
|  |  |  | min: 34:22-34:37 | Inspector Schäfermann reports that Mrs. Gollnick is convinced her husband was murdered; it is known that Gollnick was alone in the house and there is no evidence of a struggle |  |  |  |  |  |
|  |  |  | min: 1:03:38-1:05:38 | Laboratory employee explains to the inspector the principle of delayed onset of action of drugs: A capsule actually consists of two capsules -> the first shell dissolves first and the active ingredient is released (with sleeping pills you will then fall asleep). Later, the second, smaller capsule dissolves (if there is e.g. caffeine in it, you wake up again). Hence, by this principle, it is possible to administer poisons which have a delayed effect -> e.g. potassium cyanide; capsule remains would not be detectable; capsule would stay in the stomach for several hours; this time is depending on the digestion process. |  |  |  |  |  |
|  |  |  | min: 1:08:45 | Mr. Sannwald takes tablets |  |  |  |  |  |
|  |  |  | min: 1:14:27-1:14:32 | Inspector Schäfermann says that Mr. Sannwald has probably swallowed poison; he is being searched for by helicopter |  |  |  |  |  |
|  |  |  | min: 1:19:35-1:19:45 | The inspector has the suspicion that Mrs. Glogau has poisoned Mr. Sannwald; he could die at any moment |  |  |  |  |  |
|  |  |  | min: 1:21:05-1:21:18 | Mr. Sannwald had taken his medication about 45 minutes to 1 hour before |  |  |  |  |  |
|  |  |  | min: 1:21:44-1:22:05 | The inspector hypothesizes that Mr. Gollnick was poisoned with a potassium cyanide capsule; this suspicion can only be confirmed by gastric lavage and capsule finding with Mr. Sannwald |  |  |  |  |  |
|  |  |  | min: 1:22:30-1:23:03 | Test for potassium cyanide in the capsule is positive; bitter almond odor |  |  |  |  |  |

| *Tatort* episode | First broadcast | Location | Time period | Action | Active ingredient/group of active ingredients | Mechanism of action/symptoms | Literature | Plausibility/Detailedness | Time comparison |
| --- | --- | --- | --- | --- | --- | --- | --- | --- | --- |
| *Late Vintage (75) (1)* | 22.05.1977 | Essen | min: 1:30:01-1:30:04 | Mr. Waarst takes a tablet from the cupboard | No substance is mentioned | No mechanism, because no substance is mentioned | No literature because no substance is named; **(1)** Staudte (1977) | **Symptoms:** Respiratory distress, circulatory collapse - unclear because of which substance; **Mechanism of action:** no; **Substance explanation:** no; **Latency:** no; **Dose:** no; **Cause of death:** no; Route of administration: oral - plausible; **P:** cannot be rated as no substance was mentioned; **D:** 6 | no |
|  |  |  | min: 1:31:40-1:32:06 | Mr. Waarst can hardly breathe and walk down the stairs; then he collapses; you can see the box which contained the tablet -> it just says "poison" on it; "The poison is lethal." |  |  |  |  |  |

| *Tatort* episode | First broadcast | Location | Time period | Action | Active ingredient/group of active ingredients | Mechanism of action/symptoms | Literature | Plausibility/Detailedness | Time comparison |
| --- | --- | --- | --- | --- | --- | --- | --- | --- | --- |
| *Vodka Bitter Lemon (50) (1)* | 13.04.1975 | Essen | min: 06:57-07:13 | Mr. Koenen prepares a lemon vodka for him and the young woman Irene Lersch; he puts some ice cubes in a glass | see episode 437 | see episode 437 | see episode 437; **(1)** Wirth (1975) | **Symptoms:** red spots - plausible; **Mechanism of action:** no; **Substance explanation:** bitter almond odor - plausible; use in electroplating - plausible; **Latency:** no; **Dose:** no; **Cause of death:** no; **Route of administration:** oral - plausible; **P:** 1; **D:** 5 | Comparison with "Licorice," "Bitter Almonds," and  "Harm set, harm get" with regard to potassium cyanide presentation |
|  |  |  | min: 08:45-09:05 | Irene Lersch repeatedly takes a sip of her drink |  |  |  |  |  |
|  |  |  | min: 09:36-10:30 | Mr. Koenen finds Irene Lersch lying unconscious on the floor; he feels for the pulse and finds that it is not present; then he smells the empty glass; he wants to call 911, but finally does not do so |  |  |  |  |  |
|  |  |  | min: 12:49-13:11 | Discovery of the body of Irene Lersch; the forensic pathologist suspects poisoning; "Most likely potassium cyanide. The red spots on the body and the smell of bitter almond are typical characteristics"; presumably oral intake. |  |  |  |  |  |
|  |  |  | min: 21:05-21:14 | Inspector Haferkamp asks Mr. Koenen if he works with toxic substances in his company (cyanide compounds such as potassium cyanide); Mr. Koenen answers in the negative, but is not sure and wants to get some information |  |  |  |  |  |
|  |  |  | min: 40:58-41:17 | "Cyanide compounds are usually needed for electroplating, silver plating, gold plating, in the photo lab – the developer solution also contains potassium cyanide." |  |  |  |  |  |
|  |  |  | min: 54:00-54:08 | Mrs. Koenen says that she likes to paint and forge gold |  |  |  |  |  |
|  |  |  | min: 1:04:26-1:05:17 | 5 kg of potassium cyanide is stored in the goldsmith's workshop where Mrs. Koenen used to work. |  |  |  |  |  |
|  |  |  | min: 1:16:46-1:16:50 | Inspector Haferkamp is told that a pearl was frozen in an ice cube; then he suspects that the poison was also in an ice cube |  |  |  |  |  |
|  |  |  | min: 1:20:59-1:23:00 | Inspector Haferkampf explains that Mrs. Koenen had put potassium cyanide in the water which she afterward put it in the freezer of her husband's minibar -> but accidentally Irene Lersch was poisoned and not Mr. Koenen |  |  |  |  |  |

| *Tatort* episode | First broadcast | Location | Time period | Action | Active ingredient/group of active ingredients | Mechanism of action/symptoms | Literature | Plausibility/Detailedness | Time comparison |
| --- | --- | --- | --- | --- | --- | --- | --- | --- | --- |
| *Eight Years Later (39) (1)* | 28.04.1974 | Essen | min: 38:05-38:16 | Inspector Haferkamp finds a pill in Mrs. Pallenburg's bag and takes it out of the bag. | see episode 1018 | see episode 1018 | see episode 1018; **(1)** Becker (1974) | **Symptoms:** shortness of breath, nausea circulatory failure, unconsciousness - plausible; **mechanism of action:** no; **explanation of substance:** formation by incomplete combustion - plausible; odorless and tasteless - plausible; CO is sum formula - plausible; **latency:** "in the shortest time" perhaps a few minutes -> duration of climbing stair s- plausible; **dose:** no; **cause of death:** asphyxiation - plausible; **route of administration:** inhalation - plausible; warning signs to indicate gas exposure - plausible; statement that it is life-threatening to go upstairs without oxygen mask indicates dangerousness to the layman; Mr. Brossberg shows no symptoms, statement at min: 58:13 – implausible because he was not on a lower level and because wind would only create some mitigation; ambiguity about pills being put in the drink; scenario with manipulated coke heater - plausible; candles going out - plausible due to oxygen displacement; **P:** 2; **D:** 2 | Comparison with "In the End You Go Naked," "The Legal Case Reinhardt", "Burn mark", and "Fallen Angels" in  terms of carbon monoxide poisoning |
|  |  |  | min: 54:41-56:31 | There is a warning sign which says: "Caution, beware of gas. Report to the control station before entering the facility”. Mr. Brossberg and inspector Haferkamp are exactly in this area; the employees of the blast furnace carry oxygen cylinders; one of the workers guesses that the two are "tired of life" and arranges for the fire department to be called; Haferkamp becomes short of breath on the stairs, then slumps, but tries several times in vain to get up; then he lies on the floor for a moment and is conscious, and then he falls unconscious; Mr. Brossberg, on the other hand, shows no symptoms at all (perhaps some shortness of breath, but this may also result from the chase) |  |  |  |  |  |
|  |  |  | min: 56:45-57:15 | Haferkamp is in hospital and still looks a little weak; he is told: "You suffer from CO poisoning (...) Carbon monoxide is produced by incomplete combustion of coal or coke. These blast furnaces are all not completely tight. The wind was unfavorable."; "A few minutes longer and you would have been dead."; The doctor explains that CO is tasteless and odorless; "At sufficient concentration, it leads to death by asphyxiation in a very short time." |  |  |  |  |  |
|  |  |  | min: 58:13-59:35 | Brossberg probably suffers from the same poisoning, only in a weaker form; "Maybe because he didn't go up as high as you did and because the air current was more favorable for him down there."; However, Mr. Brossberg was actually in a higher position than Haferkamp, then came down to him and left; nausea as a symptom is mentioned. |  |  |  |  |  |
|  |  |  | min: 1:17:40-1:17:59 | Mrs. Pallenburg takes some pills out of a box and puts them into inspector Haferkamp's drink; he notices what she does |  |  |  |  |  |
|  |  |  | min: 1:18:51-1:18:59 | Haferkamp pretends to drink from his poisoned beer, but in fact he pours it out |  |  |  |  |  |
|  |  |  | min: 1:19:35 | Haferkamp pretends to fall asleep |  |  |  |  |  |
|  |  |  | min: 1:19:51-1:20:24 | Mrs. Pallenburg closes all the doors and manipulates the coke heater so that the gas can flow into the living room where the inspector is lying |  |  |  |  |  |
|  |  |  | min: 1:20:44-1:20:56 | Smoke is pouring into the room |  |  |  |  |  |
|  |  |  | min: 1:21:42-1:22:08 | Mrs. Pallenburg opens the ventilation in the living room so that the smoke with the gas flows in |  |  |  |  |  |
|  |  |  | min: 1:23:54-1:23:58 | The candles in living room go out |  |  |  |  |  |
|  |  |  | min: 1:24:35 | Inspector Haferkamp escapes the gas in time |  |  |  |  |  |

Numbers in parentheses refer to references listed in column 8. Complete references are provided in the reference list.
