# Supplementary material for: Poisoning cases in the German crime series Tatort (crime scene) from 1974 to 2022
Source: Naunyn Schmiedebergs Arch Pharmacol. 2022 Aug 16;395(11):1419–40. doi: 10.1007/s00210-022-02281-9 (PMC9568490; doi:10.1007/s00210-022-02281-9)
Supplement: Supplementary file 2 — Supplementary file2 (DOCX 28 KB) [file 210_2022_2281_MOESM2_ESM.docx]

**Supplementary Source Data**

**Case of poisoning in Tatort from 1974 to 2022**

**Rachel Ellerbeck – Roland Seifert**

**Source data to figure 2:**

| **Substance categories:** | **after 2000, adjusted** | **before 2000, adjusted** | **total*** |
| --- | --- | --- | --- |
| Drugs | 66% | 15% | 51% |
| Narcotics | 16% | 23% | 18% |
| Plant toxins | 28% | 0% | 20% |
| Synthetic toxins | 19% | 8% | 16% |
| Bacterial toxins | 6% | 0% | 4% |
| Animal toxins | 3% | 0% | 2% |
| Gases | 9% | 15% | 11% |
| Environmental pollutants | 13% | 31% | 18% |
| Rodenticides | 3% | 0% | 2% |
| Solvents | 6% | 0% | 4% |
| Metals | 6% | 0% | 4% |
| Fictitious | 19% | 31% | 22% |
| Others | 13% | 8% | 11% |

**Source data to figure 3:**

| **Substance titeling:** | **after 2000, adjusted** | **before 2000, adjusted** | **total*** |
| --- | --- | --- | --- |
| Active substance/toxin | 113% | 38% | 91% |
| Plant name | 6% | 0% | 4% |
| Active-substance group | 25% | 8% | 20% |
| Colloquial name | 69% | 38% | 60% |
| Trade name | 0% | 15% | 4% |
| Fictitious name | 22% | 31% | 24% |
| No name | 3% | 8% | 4% |
| Others | 9% | 0% | 7% |

**Source data to figure 4:**

| **Poisoning presentation:** | **after 2000, adjusted** | **before 2000, adjusted** | **total*** |
| --- | --- | --- | --- |
| Retrospective view | 50% | 54% | 51% |
| With death | 53% | 62% | 56% |
| With survival | 50% | 62% | 53% |
| Mentioned in passing | 34% | 23% | 31% |
| Others | 3% | 0% | 2% |

**Source data to figure 5:**

| **Application:** | **after 2000, adjusted** | **before 2000, adjusted** | **total*** |
| --- | --- | --- | --- |
| Oral | 84% | 54% | 76% |
| Inhalative | 16% | 46% | 24% |
| Injected/pecked/ pricked/i.v. | 25% | 23% | 24% |
| Dermal | 6% | 0% | 4% |
| Snuffed | 3% | 0% | 2% |
| Unclear | 13% | 23% | 16% |

**Source data to figure 6:**

| **Substance presentation:** | **after 2000, adjusted** | **before 2000 adjusted** | **total*** |
| --- | --- | --- | --- |
| Cardboard medicine box | 13% | 0% | 9% |
| Plant | 9% | 0% | 7% |
| Powder | 9% | 8% | 9% |
| Tablet | 25% | 15% | 22% |
| Box | 16% | 23% | 18% |
| Syringe | 13% | 23% | 16% |
| Others | 38% | 62% | 44% |
| None | 50% | 0% | 36% |

**Source data to figure 7:**

| **Etiology:** | **after 2000, adjusted** | **before 2000, adjusted** | **total*** |
| --- | --- | --- | --- |
| Accidental | 6% | 15% | 9% |
| Suicidal | 16% | 23% | 18% |
| Abusive | 22% | 15% | 20% |
| Poison delivery | 97% | 69% | 89% |

**Source data to figure 8:**

| **Repeated substances** | **after 2000, adjusted** | **before 2000, adjusted** | **total, adjusted** |
| --- | --- | --- | --- |
| Potassium cyanide | 6% | 15% | 9% |
| Cannabis | 6% | 0% | 4% |
| Ecstasy | 6% | 0% | 4% |
| Rabies virus | 6% | 0% | 4% |
| Carbon monoxide | 9% | 15% | 11% |
| Benzodiazepines | 6% | 0% | 4% |
| Barbiturates | 13% | 0% | 9% |
| Knockout drops | 9% | 8% | 9% |
| Botulinum toxin | 6% | 0% | 4% |
| Exogenous insulins | 6% | 0% | 4% |
| GHB | 6% | 0% | 4% |
| Asbestos | 0% | 15% | 4% |
| Dibenzodioxin | 3% | 8% | 4% |
| Heroin | 0% | 15% | 4% |

**Source data to figure 9:**

| **Episode rating** | **after 2000, adjusted** | **before 2000, adjusted** | ***total, adjusted** |
| --- | --- | --- | --- |
| **Best episodes** | 31% | 8% | 24% |
| **Worst episodes** | 9% | 31% | 16% |

**Source data to figure 10:**

| **Missing information** | **after 2000, adjusted** | **before 2000, adjusted** | **total*** |
| --- | --- | --- | --- |
| **Mechanism of action** | 88% | 92% | 89% |
| **Symptoms** | 13% | 15% | 13% |

**Source data to figure 11:**

| **Substance categories** | ***Tatort*** | **reality, intentional*** | **reality, accidental*** | **reality, total of 2011, 2021*** |
| --- | --- | --- | --- | --- |
| Drugs | 28% | 88% | 24% | 41% |
| Cleaning and care products | 2% | 1% | 16% | 12% |
| Plants | 11% | 1% | 6% | 11% |
| Food, beverages and narcotics | 9% | 4% | 4% | 2% |

**Source data to figure 12:**

| **Poisoning outcome** | ***Tatort*** | **Reality*** |
| --- | --- | --- |
| Cure/survival | 28% | 85% |
| Death | 55% | 1% |

**Source data to figure 13:**

| **Application** | ***Tatort*** | **Reality*** |
| --- | --- | --- |
| Oral | 51% | 86% |
| Inhalative | 17% | 7% |
| Dermal | 3% | 4% |
| Ocular | 0% | 1% |
| i.v. | 17% | 1% |

**Source data to figure 14:**

| **Etiology** | ***Tatort*** | **Reality*** |
| --- | --- | --- |
| Accidental | 6% | 63% |
| Suicidal | 13% | 26% |
| Abusive | 15% | 5% |
| Poison delivery | 66% | 1% |

**Source data to figure 15:**

|  | **Total** | **Man** | **Woman** |
| --- | --- | --- | --- |
| **Offenders** | 86 | 65 | 21 |
| **Victim** | 85 | 59 | 26 |

**Source data to figure 16:**

| **Man** | **Woman** |
| --- | --- |
| 47% | 53% |

**Source data to figure 17:**

|  | **Total** | **Man** | **Woman** | **Unknown** |
| --- | --- | --- | --- | --- |
| **Offenders** | 135 | 58 | 71 | 6 |
| **Victim** | 236 | 90 | 80 | 66 |
